# Integrated Analysis of the Lung Microbiome and Metabolome Reveals Associations Between Amino Acid Metabolism and Pulmonary Fibrosis in a Bleomycin-Induced Mouse Model

**DOI:** 10.3390/ijms27135895

**Published:** 2026-06-30

**Authors:** Chunjie Xu, Siying Qin, Peiyi Sun, Yao Meng, Congran Li, Xiukun Wang, Xuefu You, Guoqing Li, Xinyi Yang

**Affiliations:** 1 Beijing Key Laboratory of Technology and Application for Anti-Infective New Drugs Research and Development, Institute of Medicinal Biotechnology, Chinese Academy of Medical Sciences & Peking Union Medical College, Beijing 100050, China; 2 Hebei Key Laboratory of Pathogenic Mechanisms and Diagnosis & Treatment Technologies for Lung Microbiome, Beijing 100050, China; 3 State Key Laboratory of Respiratory Health and Multimorbidity, Institute of Medicinal Biotechnology, Chinese Academy of Medical Sciences & Peking Union Medical College, Beijing 100050, China; 4 Division for Medicinal Microorganism-Related Strains, CAMS Collection Center of Pathogenic Microorganisms, Beijing 100050, China; 5 State Key Laboratory of Bioactive Substances and Function of Natural Medicines, Institute of Materia Medica, Chinese Academy of Medical Sciences & Peking Union Medical College, Beijing 100050, China

**Keywords:** pulmonary fibrosis, bleomycin, lung microbiota, *Klebsiella*, metabolome

## Abstract

Idiopathic pulmonary fibrosis (IPF) is a chronic and progressive lung disease with limited therapeutic options. To investigate the roles of the pulmonary microbiota and metabolism in fibrosis, we established a bleomycin (BLM)-induced mouse model at 14- and 28-day timepoints and performed integrated 16S rRNA gene amplicon sequencing and untargeted metabolomic analyses. Histological and Western blot analyses confirmed significant fibrotic changes and the upregulation of fibrotic markers. Microbiome profiling revealed marked dysbiosis after BLM exposure, characterized by reduced microbial diversity and enrichment of *Klebsiella*. LC-MS–based metabolomic analysis identified substantial perturbations in the lung tissue metabolome, particularly in lipid metabolism, amino acid metabolism, and energy pathways. Correlation analysis indicated a strong positive association between the abundance of *Klebsiella* and the levels of specific dipeptides, including Ala-Hyp-Gly, Asp-His, and Asp-Asn. The accumulation of these dipeptides may reflect increased collagen degradation and turnover in fibrotic lungs. Collectively, our findings demonstrate that BLM-induced pulmonary fibrosis is accompanied by coordinated alterations in the lung microbiome and metabolome. Notably, microbial dysbiosis, particularly the expansion of *Klebsiella*, may be associated with alterations in amino acid metabolism and fibrotic progression.

## 1. Introduction

Idiopathic pulmonary fibrosis (IPF) is a chronic, progressive interstitial lung disease with high mortality and limited therapeutic options. The disease is characterized by alveolar epithelial cell injury, aberrant fibroblast proliferation, excessive extracellular matrix deposition, and the eventual structural destruction of lung tissue, leading to respiratory failure [1]. Its global incidence is rising, with a higher prevalence in males and a typical diagnosis around age 65. The prognosis remains poor, with a median survival of only 3–5 years post-diagnosis and a mortality rate exceeding that of many cancers [2]. Although approved antifibrotic agents, pirfenidone and nintedanib, can slow disease progression, they cannot reverse established fibrosis. Recent research on pharmacological treatment for IPF has led to some advances, with multiple drug candidates entering various stages of clinical trials. However, comprehensive understanding of IPF pathogenesis is still lacking [3], underscoring the urgent need to identify factors that drive its onset and progression.

Animal models are indispensable for elucidating disease mechanisms and evaluating therapies. The bleomycin (BLM)-induced mouse model, which triggers DNA damage and oxidative stress, is the most widely used. Delivered via intratracheal, intranasal, or intraperitoneal routes, BLM recapitulates key features of IPF, including epithelial injury, inflammation, collagen deposition, and reduced lung compliance. Despite limitations, such as its acute onset, potential for spontaneous resolution, and limited subpleural involvement, the single-dose intratracheal model remains standard due to its simplicity and reproducibility [4,5,6].

Historically considered sterile, the lung is now recognized to host a low-biomass but diverse microbial community, crucial for maintaining immune homeostasis, metabolic regulation, and epithelial integrity [7]. Dysbiosis, or microbial imbalance, has been implicated in numerous pulmonary diseases, including IPF, where reduced microbial diversity correlates with disease progression [8]. In IPF patients, bronchoalveolar lavage fluid (BALF) shows an increased abundance of *Streptococcus* and *Staphylococcus*, which is associated with poorer outcomes [9]. During acute exacerbations of IPF (AE-IPF), the bacterial load in the lungs increases markedly [10]. Intriguingly, the macrolide antibiotic azithromycin shows potential clinical benefits in IPF management [11,12], further supporting a role for microbes in disease pathogenesis.

In BLM-treated mice, microbial dysbiosis precedes peak lung injury and persists through the fibrotic phase. Germ-free mice exhibit reduced mortality after BLM exposure [13], implicating the microbiota in disease severity. Our previous work identified a marked enrichment of *Klebsiella quasipneumoniae* in fibrotic lungs, linked to sustained activation of reactive oxygen species (ROS) signaling, suggesting specific pathogenic taxa may exacerbate fibrosis [14].

Metabolic reprogramming is an emerging hallmark of fibrosis. Metabolomic studies of IPF lungs reveal dysregulated pathways, including downregulated sphingolipid and upregulated arginine metabolism [15]. Similarly, BLM-induced fibrosis in mice enriches amino acid–related pathways [16], indicating a conserved metabolic shift. Targeting these alterations is a promising therapeutic strategy. For instance, inhibition of stearoyl-CoA desaturase 1 (SCD1), downregulated in IPF lungs, induces endoplasmic reticulum stress and promotes fibrosis in mice [17]. Similarly, glutamine metabolism supports collagen production in fibroblasts. Its deprivation or inhibition suppresses TGF-β1–induced fibrotic markers [18,19,20].

Despite growing knowledge of microbial or metabolic changes in isolation, their functional interplay in pulmonary fibrosis remains poorly understood. Integrating microbiome and metabolomic data could provide crucial insights into disease mechanisms. Here, we establish a BLM-induced pulmonary fibrosis model in mice and perform integrated 16S rRNA gene amplicon sequencing and untargeted metabolomic profiling. We systematically characterize pulmonary microbial dysbiosis and explore its association with host metabolic reprogramming. This dual-omics approach aims to identify microbe-associated metabolic pathways driving fibrosis, providing novel theoretical groundwork for targeted IPF therapies.

## 2. Results

### 2.1. BLM Induces Pulmonary Fibrosis in Mice

**Figure 1:**
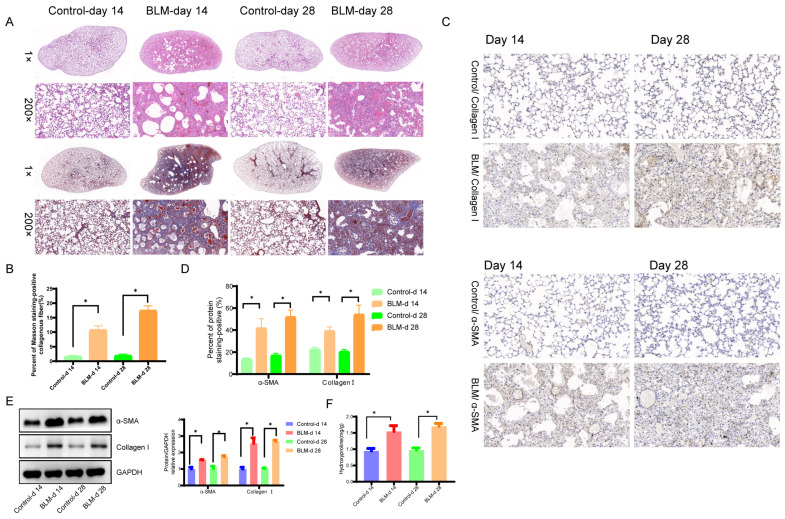
BLM-induced pulmonary fibrosis in mice. (**A**) Representative H&E and Masson’s trichrome-stained lung sections at day 14 and 28. Scale bar, 50 µm. (**B**) Quantification of fibrotic area from Masson’s trichrome staining. (**C**,**D**) IHC was performed, and the levels of α-SMA and collagen I were determined in the lungs of mice. (**E**) Western blot analysis of collagen I and α-SMA at day 14 and 28. (**F**) Hydroxyproline detection in mice. Data are mean ± SD (*n* = 3). * *p* < 0.05 vs. control.

To evaluate the fibrotic effects of bleomycin (BLM), mice were administered a single intratracheal dose (5 mg/kg in 50 µL saline) and lung tissues were harvested at day-14 and −28 post-exposure. Hematoxylin and eosin (H&E) staining revealed normal alveolar architecture in control mice, whereas BLM-treated lungs exhibited disrupted alveolar structures, thickened septa, and pronounced inflammatory cell infiltration at both time points (Figure 1A,B). Masson’s trichrome staining confirmed substantial collagen deposition (blue) in alveolar and perivascular regions of BLM-treated mice, and both time and BLM treatment were statistically significant (*p* < 0.05), indicating progressive fibrosis (Figure 1A,B). Immunohistochemistry for collagen I further demonstrated enhanced and patchy deposition in the interalveolar septa and peribronchial areas following BLM exposure, and both time and BLM treatment were statistically significant (*p* < 0.05) (Figure 1C,D). Consistent with histopathology, Western blot and hydroxyproline analysis showed a significant increase in the protein levels of fibrotic markers collagen I and α-SMA in BLM-treated lungs, and BLM treatment is shown to play a major role (*p* < 0.05) (Figure 1E,F). Collectively, these data demonstrate that BLM administration successfully induces structural damage, inflammation, and fibrotic remodeling in mouse lungs.

### 2.2. BLM Induces Significant Changes in the Lung Microbiota of Mice

**Figure 2:**
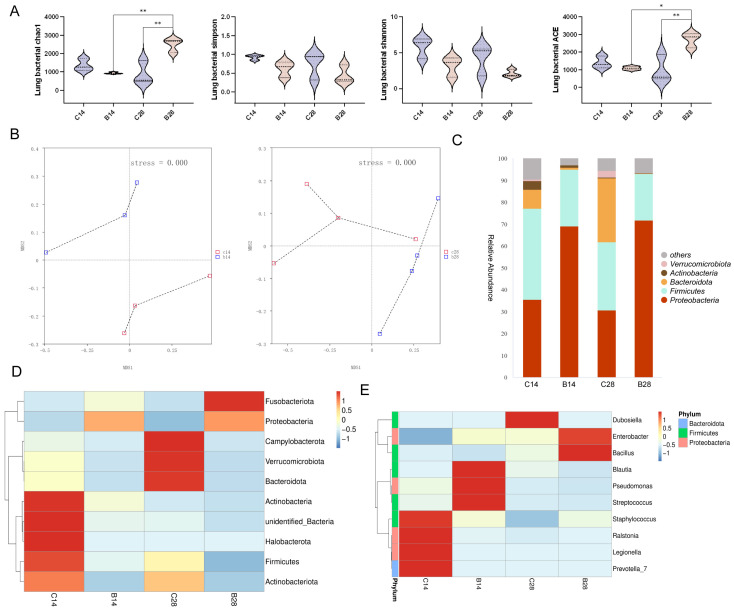
BLM alters the composition of lung microbiota. (**A**) Alpha diversity indices. (**B**) NMDS plot of microbial community structure. (**C**) Relative abundance of bacterial phyla. (**D**) Heatmap of the top 10 phyla. (**E**) Heatmap of the top 10 genera. * *p* < 0.05 ** *p* < 0.01 vs. control group, *n* = 3 mice per group.

We performed 16S rRNA gene amplicon sequencing to assess the impact of BLM on the lung microbiome. Alpha diversity indices (Chao1, ACE) were significantly lower in the day-28 BLM group compared to the day-28 control and day-14 BLM groups (*p* < 0.05; Figure 2A), indicating a progressive decline in microbial richness over time. Non-metric multidimensional scaling (NMDS) further indicated substantial shifts in microbial community structure following BLM administration, with clear separation between BLM-treated and control samples at days 14 and 28 (Figure 2B), and the PERMANOVA analysis showed that there was statistical significance at 28 days (*p* < 0.05). Taxonomic profiling at the phylum level demonstrated consistent alterations in the BLM-treated groups, characterized by a significant increase in the relative abundance of *Proteobacteria* and a concurrent decrease in *Bacteroidetes* compared with controls (Figure 2C). A heatmap of the top ten most abundant phyla corroborated these compositional changes (Figure 2D). At the genus level, heatmap analysis highlighted a pronounced enrichment of *Enterobacter* in BLM-treated animals relative to controls (Figure 2E). These data indicate that BLM exposure progressively reduces lung microbial diversity and drives significant reorganization of microbial community composition.

**Figure 3:**
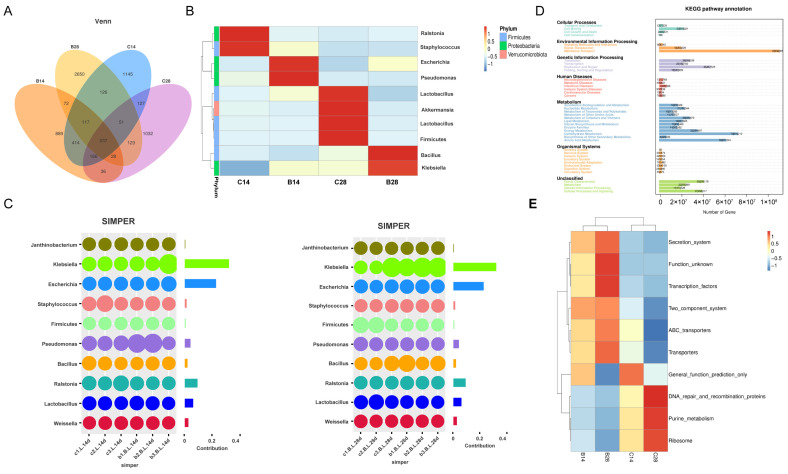
Microbial community shifts and predicted functional changes. (**A**) Venn diagram of shared and unique bacterial genera. (**B**) Heatmap of the top 10 species. (**C**) SIMPER analysis showing key contributing species. (**D**,**E**) KEGG pathway enrichment analysis of predicted metagenomic functions at (**D**) level 2 and (**E**) level 3. *n* = 3–5 mice per group.

Venn analysis of operational taxonomic units (OTUs) identified a core microbiota of 237 OTUs shared among all groups. BLM-treated groups (B14, B28) contained more group-specific OTUs than controls, with B28 showing the highest number of unique OTUs (*n* = 2650; Figure 3A). In contrast, control groups shared a larger proportion of OTUs and contained fewer unique taxa (C14: 1145; C28: 1032), reflecting greater community stability over time (Figure 3A). These results demonstrate that BLM administration substantially restructures the lung microbiota, with divergence from controls increasing progressively.

Among the ten most abundant genera, *Klebsiella* was significantly enriched in BLM-treated mice at both day 14 and day 28 relative to controls, with its abundance further elevated at day 28 (Figure 3B; *p*< 0.05), indicating progressive expansion during fibrotic progression. Similarity Percentage (SIMPER) analysis identified *Klebsiella* as the primary contributor to community-level differences between BLM and control groups, accounting for 27.9% (day 14) and 37.6% (day 28) of the observed dissimilarity (Figure 3C; *p* < 0.05).

Functional potential of the microbiota was inferred using PICRUSt based on 16S rRNA data (Figure 3D). KEGG pathway enrichment analysis revealed that, at level 2, categories related to membrane transport, carbohydrate metabolism, and amino acid metabolism were predominant (Figure 3E). At level 3, BLM-treated mice showed reduced predicted activity in pathways associated with DNA repair and recombination proteins, purine metabolism, and ribosome biogenesis compared with controls (Figure 3E).

Collectively, these results demonstrate that BLM induces progressive restructuring of the lung microbiota, marked by reduced diversity, expansion of *Klebsiella*, and altered predicted metabolic function, particularly in amino acid metabolism.

### 2.3. BLM Exposure Disrupts Lung Metabolism

**Figure 4:**
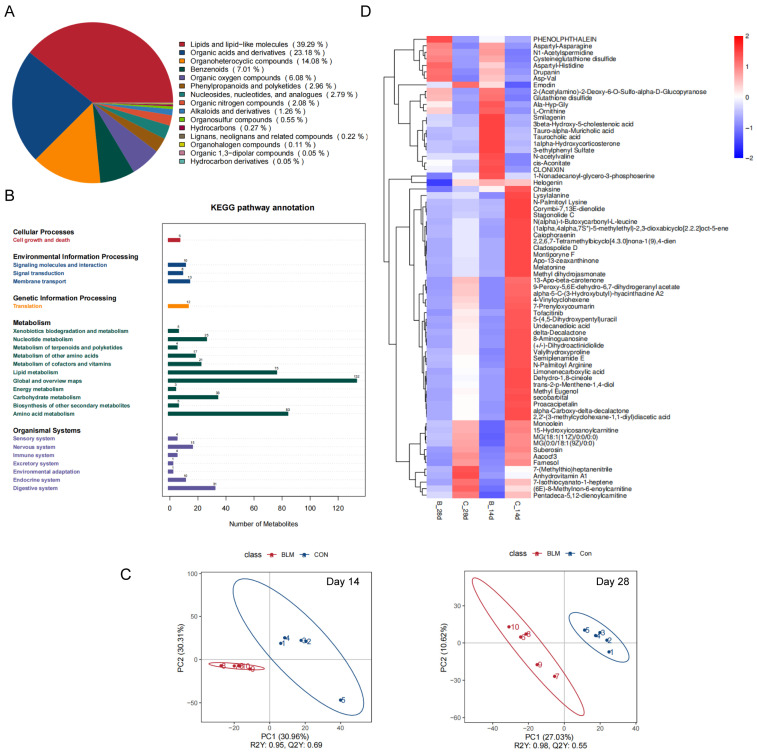
BLM-induced alterations in the lung metabolome. (**A**) Classification of significantly altered metabolites. (**B**) KEGG pathway enrichment (level 2) of differential metabolites. (**C**) PLS-DA score plots. (**D**) Heatmap of all differential metabolites. *n* = 3–5 mice per group.

To evaluate the metabolic consequences of BLM exposure on lung tissue, we performed liquid chromatography–tandem mass spectrometry (LC-MS/MS) to profile metabolite changes. Among the detected metabolites (Figure 4A), lipids and lipid-like molecules constituted the largest class (39.29%), followed by organic acids and derivatives (23.18%). Enrichment analysis of KEGG pathways (level 2) indicated that the altered metabolites were predominantly linked to “global and overview maps” and “amino acid metabolism” (Figure 4B). Partial least squares discriminant analysis (PLS-DA) revealed a clustering trend between the control and BLM-treated groups. The PLS-DA model parameters were R^2^ = 0.95, Q^2^ = 0.69 at day 14 and R^2^ = 0.98, Q^2^ = 0.55 at day 28, reflecting acceptable model fit and predictive performance. At day 14, principal components 1 and 2 (PC1 and PC2) accounted for 30.96% and 30.31% of the total variance, respectively. By day 28, PC1 and PC2 explained 27.03% and 10.62% of the variance, reflecting a time-dependent shift in metabolic profiles after BLM administration (Figure 4C). Differential metabolites were further filtered based on fold-change thresholds. The total numbers of screened metabolites are summarized in Figure 4D.

**Figure 5:**
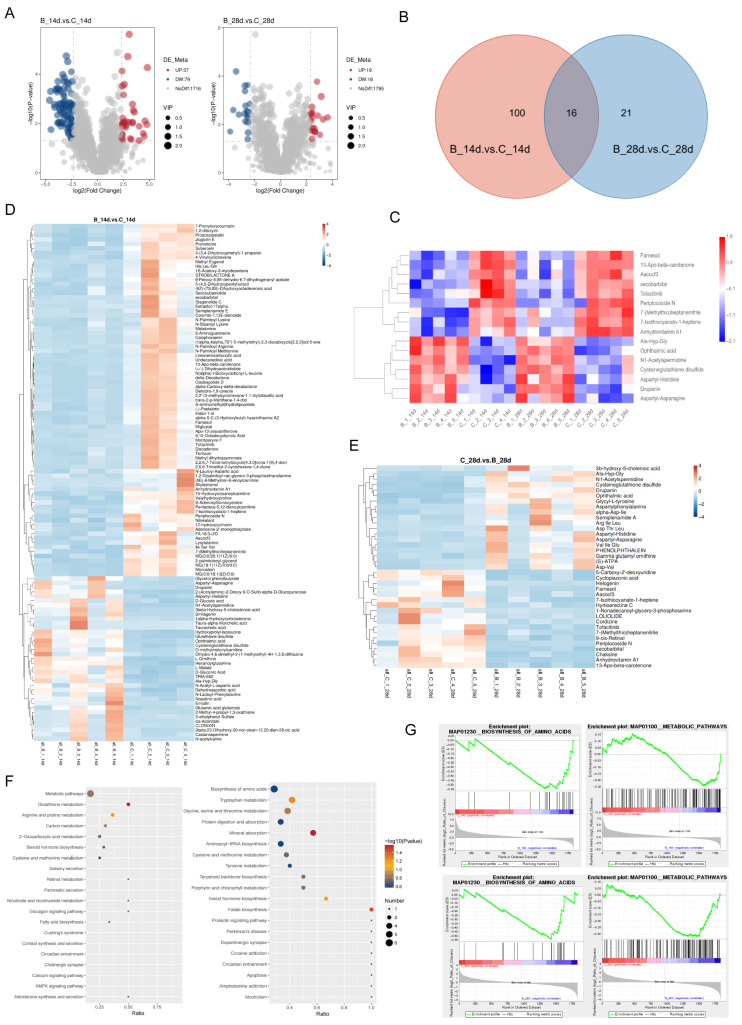
Analysis of differential metabolites. (**A**) Volcano plots of metabolites at days 14 and 28. (**B**) Venn diagram of differential metabolites. (**C**) Heatmap of 16 common differential metabolites. (**D**,**E**) Heatmaps of differential metabolites at (**B**) day 14 and (**C**) day 28. (**F**) KEGG pathway enrichment (level 3). (**G**) Gene Set Enrichment Analysis (GSEA). *n* = 3–5 mice per group.

Volcano plot analysis (Figure 5A) identified 116 significantly altered metabolites (FDR < 0.05) at day 14 (37 upregulated, 79 downregulated) and 37 at day 28 (19 upregulated, 18 downregulated). Sixteen metabolites were consistently altered across all experimental groups (*p* < 0.05, Figure 5B/C). KEGG pathway enrichment analysis (level 3) revealed that metabolites altered at day 14 were mainly associated with metabolic pathways, glutathione metabolism, and arginine and proline metabolism (Figure 5D). At day 28, enriched pathways included amino acid biosynthesis, tryptophan metabolism, and glycine, serine, and threonine metabolism (Figure 5E). Notably, these amino acid–related pathways overlapped with functional predictions derived from lung microbiota data (Figure 5F/G), suggesting a potential connection between microbial dysbiosis and host amino acid metabolic remodeling during BLM-induced pulmonary fibrosis.

### 2.4. Altered Pulmonary Microbiota and Metabolites Influence Fibrosis Progression

**Figure 6:**
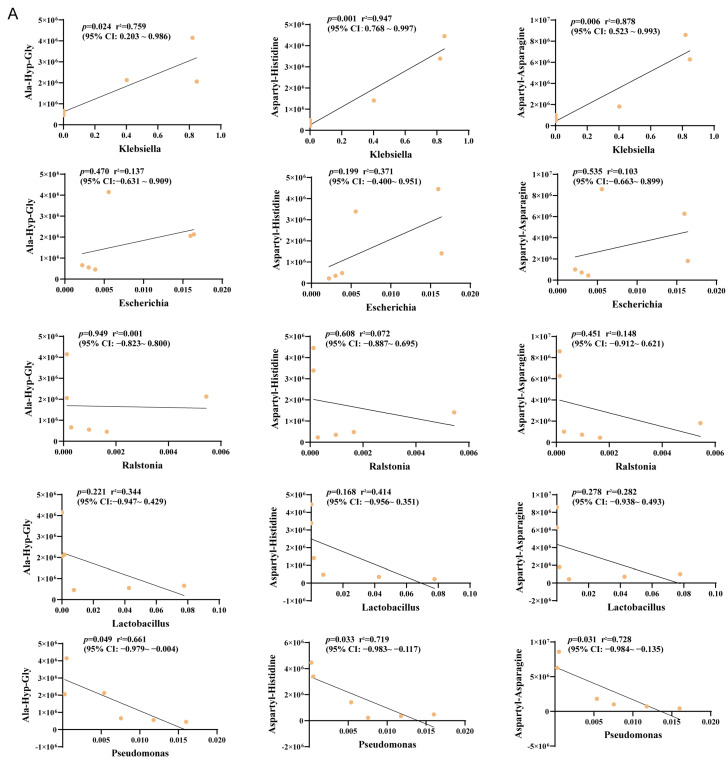
Correlation between lung microbiota and metabolome. (**A**) Scatter plots showing Pearson correlations between *Klebsiella* (or other bacterial species, including *Escherichia coli*, *Ralstonia pickettii*, *Lactobacillus murinus*, *Pseudomonas azotoformans*) abundance and selected dipeptides. *n* = 3–5 mice per group.

Our data demonstrate that BLM administration induces significant changes in both the pulmonary microbiota and the lung metabolome of mice. To explore potential interactions between these datasets, we performed Pearson correlation analysis using significantly altered microbial taxa and metabolites identified in BLM-treated versus control animals. Notably, *Klebsiella*, which showed a marked increase in relative abundance post-BLM, exhibited strong positive correlations with several dipeptides that were enriched at day 28. These included Ala-Hyp-Gly (r^2^ = 0.759, *p* < 0.05), Asp-His (r^2^ = 0.947, *p* < 0.05), and Asp-Asn (r^2^ = 0.878, *p* < 0.05) (Figure 6A). However, there was no difference at day 14 between microbial taxa and metabolites. Collectively, these data only demonstrate a statistically significant correlation between elevated abundance of *Klebsiella* and increased levels of three dipeptides. Such correlation cannot confirm direct functional linkage, microbial dipeptide utilization or metabolic regulation effect; relevant biological mechanism requires further independent functional experimental verification in future studies.

## 3. Discussion

Idiopathic pulmonary fibrosis (IPF) progression is characterized by a shift from chronic inflammation to fibrotic remodeling, driven by complex interactions between pro-inflammatory and pro-fibrotic mediators [21]. Our previous work demonstrated that bleomycin (BLM) exposure induces pulmonary microbiota dysbiosis and fibrosis, with *Klebsiella* expansion implicated in elevating ROS levels and the key fibrotic mediator TGF-β1 [14]. To further delineate how lung microbial imbalance contributes to BLM-induced fibrosis, this study adopted an integrated amino acid metabolomic perspective. We found that microbiota dysbiosis is associated with fibrosis alongside reprogrammed amino acid metabolism. The identified differential metabolites were enriched predominantly within amino acid metabolic pathway, suggesting that pulmonary microbiota composition and amino acid metabolite levels are co-associated with fibrotic severity in the bleomycin-induced mouse model. From a multi-omics viewpoint, lung microbiota dysbiosis may facilitate fibrotic progression by altering host amino acid metabolism.

16S rRNA gene amplicon sequencing has been widely employed to investigate host–microbe interactions in disease [22]. Sequencing of the V3–V4 region here revealed persistent lung microbiota dysbiosis throughout disease progression. The dominant phyla in healthy lungs, *Bacteroidetes*, *Firmicutes*, *Proteobacteria*, and *Actinobacteria*, align with earlier reports [23]. Previous studies have shown that although the respiratory tract microbial communities fluctuate in response to changes in pH, temperature, and oxygen levels, they overall remain relatively stable under healthy conditions. In the onset and progression of pulmonary fibrosis, however, microbial dysbiosis not only precedes pathological damage but also persists throughout the entire disease course [13,24]. Clinical evidence indicates that in patients with idiopathic pulmonary fibrosis (IPF), the alpha diversity of the lung microbiota is reduced compared with healthy individuals, while changes are also observed in species number and relative abundance (beta diversity) [25,26]. Consistent with the clinical observations in IPF patients, we observed reduced alpha diversity and altered beta diversity in BLM-treated mice. The dynamic fluctuations at phylum, genus and species levels suggest that pathological changes may be driven by the competitive expansion of specific taxa, such as *K. quasipneumoniae*, and loss of commensals [7].

Changes in the pulmonary microbiota are closely linked to alterations in lung metabolites. For instance, PM2.5 exposure has been shown to perturb both lung amino acid metabolites and microbiota composition [27]. Our untargeted LC–MS metabolomics identified lipids/lipid-like molecules (39.29%) and organic acids/derivatives (23.18%) as the most altered metabolite classes after BLM injury, echoing earlier findings [28]. Notably, *Klebsiella* was significantly enriched in BLM-treated mice, and KEGG enrichment analysis indicated that metabolite changes were strongly associated with amino acid metabolism. Amino acids serve not only as protein precursors but also as signaling molecules and redox regulators [29]. In lung pathologies such as COPD and IPF, metabolites including creatine, proline and hydroxyproline are often elevated, reflecting aberrant amino acid metabolism [30,31,32].

In our model, day 14 differential metabolites were linked to metabolic pathways, glutathione (GSH) metabolism, and arginine/proline metabolism, whereas day 28 metabolites were enriched in amino acid biosynthesis, tryptophan metabolism, and glycine/serine/threonine metabolism. GSH is a critical intracellular antioxidant. Its dysregulation promotes fibrosis via ROS accumulation and TGF-β upregulation [14]. We previously reported that microbiota dysbiosis can induce mitophagy and energy deficits, aligning with the metabolic disturbances observed at day 14. At day 28, altered serine and threonine levels are relevant given that TGF-β receptors possess serine/threonine kinase activity, and arginine–serine motifs occur near TGF-β bioactive regions—both essential for TGF-β-mediated fibrotic signalling [14,33].

Strikingly, altered pulmonary metabolites correlated strongly with specific microbiota shifts. *Klebsiella* abundance positively correlated with several dipeptides, including Ala-Hyp-Gly (r^2^ = 0.872, *p* < 0.05), Asp-His (r^2^ = 0.947, *p*< 0.05) and Asp-Asn (r^2^ = 0.939, *p* < 0.05). These correlations suggest active crosstalk between the lung microbiota and host amino acid metabolism, pointing to a role for microbial-metabolite interactions in BLM-induced fibrosis.

This study identifies reduced pulmonary microbial biomass as a relevant factor in IPF progression. By integrating 16S rRNA gene amplicon sequencing with untargeted metabolomics, we provide novel insights into IPF pathogenesis. The dynamic nature of the lung microbiome underscores its potential as a disease modifier. Our findings that BLM alters both microbiota structure and amino acid metabolic pathways suggest that interventions targeting the microbiome—such as selective antibiotic regimens—may offer new avenues for modulating metabolic dysregulation and mitigating fibrosis [34]. These results highlight the lung microbiome and its metabolic output as promising targets for understanding and intervening in IPF.

This study has several limitations. First, the metabolomics sample size was small, which may affect the robustness of multivariate analyses such as PLS-DA. PLS-DA results should be interpreted as exploratory clustering trends rather than definitive evidence. Larger sample sizes and full model validation are needed in future studies. Lastly, initial correlations were calculated on preselected differential metabolites/taxa, yet all correlative findings cannot establish causal relationships between Klebsiella expansion and amino acid metabolic changes.

## 4. Materials and Methods

### 4.1. Animals and Treatments

Twenty male C57BL/6J mice (weighing 20–22 g), purchased from SPF Biotechnology Co., Ltd. (Beijing, China), were randomly assigned to four groups (*n* = 5 per group): day-14 control, day-14 BLM, day-28 control, and day-28 BLM. All animals had free access to food and water. Mice were housed in a temperature-controlled room (24 ± 1 °C) under a 12:12-h light/dark cycle with ad libitum access to food and water. For anesthesia, mice received an intraperitoneal injection of tribromoethanol (250 mg/kg; Sigma, St. Louis, MO, USA). After a one-day acclimatization period, mice in the BLM groups received a single intratracheal instillation of BLM (Nippon Kayaku, Tokyo, Japan) at a dose of 5 mg/kg in 50 µL sterile saline. Control mice received an equal volume of sterile saline only. Mice were euthanized on day 14 or day 28 post-instillation, and lung tissues were harvested for analysis. The left lung (which had been clamped to prevent lavage fluid entry, *n* = 3–5 per group) was carefully removed. The left lung was then divided into three approximately equal sections using a sterile razor blade: Section 1 (approximately 30 mg): Immediately snap-frozen in liquid nitrogen and stored at −80 °C for metabolomics analysis. Section 2 (approximately 30 mg): Snap-frozen in liquid nitrogen and stored at −80 °C for Western blot and hydroxyproline assay. Section 3 (the remaining portion, approximately 20–30 mg): Fixed in 4% paraformaldehyde for 24 h at 4 °C, then embedded in paraffin for histology (Masson’s trichrome and IHC). For the right lung (which had been lavaged), the tissue was snap-frozen in liquid nitrogen and stored at −80 °C as a backup. No analyses were performed on the right lung tissue in this study to avoid confounding from lavage procedure-related changes. The study protocol abided by the principle of reduction, replacement, and refinement described in the National Institute of Health Guide for the Care and Use of Laboratory Animals.

### 4.2. Histopathological and Immunohistochemical Staining

Lung tissues were fixed in 4% paraformaldehyde, embedded in paraffin, and sectioned. Hematoxylin and eosin (H&E) staining and Masson’s trichrome staining were performed to assess general morphology and collagen deposition, respectively. For immunohistochemistry, sections were stained with a primary antibody against Collagen I and α-smooth muscle actin (α-SMA) to evaluate fibrotic activity. Masson’s trichrome staining was expressed as the percentage of collagen-positive area relative to total tissue area. For immunohistochemistry targeting Collagen I and α-SMA, we used the positive pixel detection function in ImageJ with fixed intensity thresholds to calculate the percentage of positively stained area.

All staining procedures were conducted by Servicebio Biotechnology Co., Ltd. (Beijing, China).

### 4.3. Western Blot and Hydroxyproline

Total protein was extracted from lung tissues using RIPA lysis buffer supplemented with protease and phosphatase inhibitors. Protein concentration was determined using a BCA Protein Assay Kit (Thermo Fisher Scientific, Waltham, MA, USA). Equal amounts of protein (20 µg) were separated by SDS-PAGE and transferred onto polyvinylidene difluoride (PVDF) membranes (Millipore, Billerica, MA, USA). Membranes were blocked with 5% non-fat milk in TBST (Tris-buffered saline with 0.1% Tween-20) for 1 h at room temperature and then incubated overnight at 4 °C with primary antibodies against Collagen I (ab260043, 1:1000, Abcam, Cambridge, MA, USA) and α-SMA (ab7817, 1:1000, Abcam, MA, USA). After washing, membranes were incubated with HRP-conjugated secondary antibodies for 1 h at room temperature. Protein bands were visualized using an enhanced chemiluminescence (ECL) detection system (Bio-Rad, Hercules, CA, USA) and quantified with ImageJ 1.8.0.345 software.

Hydroxyproline content was measured using a commercial kit (Nanjing Jiancheng Bioengineering Institute, Nanjing, China). Lung tissues were hydrolyzed under alkaline conditions, then adjusted to pH 6.0–6.8 and brought to 10 mL with double-distilled water. Activated carbon was added to remove chromophores. After centrifugation (3500 rpm, 10 min), the supernatant was sequentially incubated with Reagent I (10 min, RT), Reagent II (5 min, RT), and Reagent III (15 min, 60 °C). Following a final centrifugation, absorbance was measured at 550 nm.

### 4.4. 16S rRNA Gene Amplicon Sequencing for the Lung Microbiome

Following euthanasia, the lungs were exposed via a midline thoracotomy. The trachea was cannulated with a 24-gauge catheter. For each mouse, the left lung was clamped at the main bronchus using a hemostat. To collect bronchoalveolar lavage fluid (BALF), the right lung was lavaged three times with 0.5 mL of sterile, ice-cold PBS (total volume 1.5 mL) through the tracheal catheter. The recovered BALF (typically 1.2–1.4 mL per mouse) was centrifuged at 500× *g* for 10 min at 4 °C. The cell pellet was discarded, and the supernatant was stored at −80 °C for subsequent DNA extraction and 16S rRNA gene sequencing. Alveolar lavage fluid was collected aseptically. Genomic DNA was extracted using a CTAB/SDS-based method (proteinase K at 56 °C for 1 h, then 10% SDS at 65 °C for 30 min). The V3–V4 region of the bacterial 16S rRNA gene was amplified with specific primers (341F: CCTAYGGGRBGCASCAG; 806R: GGACTACNNGGGTATCTAAT), and sequencing libraries were constructed using the Ion Plus Fragment Library Kit (Thermo Fisher Scientific, Massachusetts, USA). High-throughput sequencing was performed on an Ion S5^TM^ XL platform (Thermo Fisher Scientific, USA). Negative controls were included in all PCR reactions. Sequence data were processed through a quality-control pipeline to obtain high-fidelity reads. UPARSE (v7.0.1001) clustered effective tags into OTUs at 97% identity. Representative sequences were annotated using Mothur with SILVA138.1 database (threshold 0.8–1.0). MUSCLE (v3.8.31) performed multiple sequence alignment for phylogenetic analysis. Data were normalized to the minimum sample size for downstream alpha and beta diversity analyses. Microbial community function was predicted using Phylogenetic Investigation of Communities by Reconstruction of Unobserved States (PICRUSt 1.1.4). Data are available at NCBI with accession number PRJNA1479564.

### 4.5. Lung Metabolomics Analysis

Lung tissues were immediately frozen in liquid nitrogen after collection. Subsequently, ~50 mg of lung tissue was homogenized in 80% methanol containing 0.1% formic acid. Following centrifugation, the supernatant was dried and reconstituted in 10% methanol with internal standards. Metabolites were separated on an ACQUITY UPLC HSS T3 column using a water-acetonitrile gradient (0.1% formic acid) at a flow rate of 0.3 mL/min. Mass spectrometry was performed on a Q Exactive HF-X Orbitrap equipped with HESI operating in both positive and negative modes. Full-scan mass spectra (*m*/*z* 100–1500, resolution 120,000) were acquired, followed by data-dependent acquisition (DDA) MS/MS (top 10 precursors, normalized collision energy 30 eV, resolution 15,000). Metabolomic profiling of the functional potential of the microbiota was inferred using PICRUSt based on 16S rRNA data, as performed by Novogene Co., Ltd. (Beijing, China) using liquid chromatography–mass spectrometry (LC-MS). Raw data were processed with Compound Discoverer 3.3 software for peak alignment, detection, and quantification. Metabolites were annotated using the KEGG, HMDB, and LIPID MAPS databases. Partial least squares-discriminant analysis (PLS-DA) and hierarchical clustering were used to examine group differences. Differential metabolites were visualized in volcano plots and heatmaps, and pathway enrichment analysis was conducted to interpret the metabolic changes. Data are available at MetaboLights with accession number REQ20260601220193.

### 4.6. Statistical Analysis

All analyses were performed using SPSS 19.0. Data conforming to a normal distribution are presented as mean ± standard deviation (SD). Our primary analytical aim was pairwise comparison among four discrete experimental groups; thus, two-way ANOVA followed by Tukey’s HSD post hoc test was used for multi-group comparisons; two-group comparisons adopted Student’s *t*-test.

For microbiome and untargeted metabolomics differential analyses, the Benjamini–Hochberg false discovery rate (FDR) method was used to adjust *p*-values for multiple testing. Taxa or metabolites with FDR < 0.05 were considered statistically significant. Alpha and beta diversity differences were assessed using Student’s *t*-test, Wilcoxon rank-sum test, Tukey’s HSD test, or Kruskal–Wallis rank-sum test as appropriate, with FDR correction applied for multiple diversity metrics.

KEGG pathway enrichment analyses for both microbiome-predicted functions and metabolomics data were performed using the Benjamini–Hochberg method, with FDR < 0.05 indicating significant enrichment. Pearson correlation analysis was used to explore associations between microbial taxa and metabolites. A *p*-value (or FDR) < 0.05 was considered statistically significant.

## Data Availability

The original contributions presented in this study are included in the article/Supplementary Material. Further inquiries can be directed to the corresponding authors.

## Supplementary Materials

The following supporting information can be downloaded at: https://www.mdpi.com/article/10.3390/ijms27135895/s1.

## References

[1] Richeldi L., Collard H.R., Jones M.G. (2017). Idiopathic pulmonary fibrosis. Lancet.

[2] Lederer D.J., Martinez F.J. (2018). Idiopathic Pulmonary Fibrosis. N. Engl. J. Med..

[3] Bonella F., Spagnolo P., Ryerson C. (2023). Current and Future Treatment Landscape for Idiopathic Pulmonary Fibrosis. Drugs.

[4] Moeller A., Ask K., Warburton D., Gauldie J., Kolb M. (2008). The bleomycin animal model: A useful tool to investigate treatment options for idiopathic pulmonary fibrosis?. Int. J. Biochem. Cell Biol..

[5] Moss B.J., Ryter S.W., Rosas I.O. (2022). Pathogenic Mechanisms Underlying Idiopathic Pulmonary Fibrosis. Annu. Rev. Pathol..

[6] Mouratis M.A., Aidinis V. (2011). Modeling pulmonary fibrosis with bleomycin. Curr. Opin. Pulm. Med..

[7] Cuthbertson L., Walker A.W., Oliver A.E., Rogers G.B., Rivett D.W., Hampton T.H., Ashare A., Elborn J.S., De Soyza A., Carroll M.P. (2020). Lung function and microbiota diversity in cystic fibrosis. Microbiome.

[8] Natalini J.G., Singh S. (2023). Segal LN: The dynamic lung microbiome in health and disease. Nat. Rev. Microbiol..

[9] Han M.K., Zhou Y., Murray S., Tayob N., Noth I., Lama V.N., Moore B.B., White E.S., Flaherty K.R., Huffnagle G.B. (2014). Lung microbiome and disease progression in idiopathic pulmonary fibrosis: An analysis of the COMET study. Lancet Respir. Med..

[10] Spagnolo P., Molyneaux P.L., Bernardinello N., Cocconcelli E., Biondini D., Fracasso F., Tiné M., Saetta M., Maher T.M., Balestro E. (2019). The Role of the Lung’s Microbiome in the Pathogenesis and Progression of Idiopathic Pulmonary Fibrosis. Int. J. Mol. Sci..

[11] Kawamura K., Ichikado K., Yasuda Y., Anan K., Suga M. (2017). Azithromycin for idiopathic acute exacerbation of idiopathic pulmonary fibrosis: A retrospective single-center study. BMC Pulm. Med..

[12] Macaluso C., Maritano Furcada J., Alzaher O., Chaube R., Chua F., Wells A.U., Maher T.M., George P.M., Renzoni E.A., Molyneaux P.L. (2019). The potential impact of azithromycin in idiopathic pulmonary fibrosis. Eur. Respir. J..

[13] O’Dwyer D.N., Ashley S.L., Gurczynski S.J., Xia M., Wilke C., Falkowski N.R., Norman K.C., Arnold K.B., Huffnagle G.B., Salisbury M.L. (2019). Lung Microbiota Contribute to Pulmonary Inflammation and Disease Progression in Pulmonary Fibrosis. Am. J. Respir. Crit. Care Med..

[14] Xu C., Sun P., Jiang Q., Meng Y., Dong L., Wang X., Hu X., Li C., Li G., Zheng R. (2025). Tissue-resident Klebsiella quasipneumoniae contributes to progression of idiopathic pulmonary fibrosis by triggering macrophages mitophagy in mice. Cell Death Discov..

[15] Zhao Y.D., Yin L., Archer S., Lu C., Zhao G., Yao Y., Wu L., Hsin M., Waddell T.K., Keshavjee S. (2017). Metabolic heterogeneity of idiopathic pulmonary fibrosis: A metabolomic study. BMJ Open Respir. Res..

[16] Washimkar K.R., Tomar M.S., Kulkarni C., Verma S., Shrivastava A., Chattopadhyay N., Mugale M.N. (2023). Longitudinal assessment of bleomycin-induced pulmonary fibrosis by evaluating TGF-β1/Smad2, Nrf2 signaling and metabolomic analysis in mice. Life Sci..

[17] Romero F., Hong X., Shah D., Kallen C.B., Rosas I., Guo Z., Schriner D., Barta J., Shaghaghi H., Hoek J.B. (2018). Lipid Synthesis Is Required to Resolve Endoplasmic Reticulum Stress and Limit Fibrotic Responses in the Lung. Am. J. Respir. Cell Mol. Biol..

[18] Ge J., Cui H., Xie N., Banerjee S., Guo S., Dubey S., Barnes S., Liu G. (2018). Glutaminolysis Promotes Collagen Translation and Stability via α-Ketoglutarate-mediated mTOR Activation and Proline Hydroxylation. Am. J. Respir. Cell Mol. Biol..

[19] Bernard K., Logsdon N.J., Benavides G.A., Sanders Y., Zhang J., Darley-Usmar V.M., Thannickal V.J. (2018). Glutaminolysis is required for transforming growth factor-β1-induced myofibroblast differentiation and activation. J. Biol. Chem..

[20] Hamanaka R.B., O’Leary E.M., Witt L.J., Tian Y., Gökalp G.A., Meliton A.Y., Dulin N.O., Mutlu G.M. (2019). Glutamine Metabolism Is Required for Collagen Protein Synthesis in Lung Fibroblasts. Am. J. Respir. Cell Mol. Biol..

[21] Ou L., Zhang P., Huang Z., Cheng Y., Miao Q., Niu R., Hu Y., Chen Y. (2023). Targeting STING-mediated pro-inflammatory and pro-fibrotic effects of alveolar macrophages and fibroblasts blunts silicosis caused by silica particles. J. Hazard. Mater..

[22] Goraya M.U., Li R., Mannan A., Gu L., Deng H., Wang G. (2022). Human circulating bacteria and dysbiosis in non-infectious diseases. Front. Cell Infect. Microbiol..

[23] Moffatt M.F., Cookson W.O. (2017). The lung microbiome in health and disease. Clin. Med..

[24] Dickson R.P., Erb-Downward J.R., Freeman C.M., McCloskey L., Beck J.M., Huffnagle G.B., Curtis J.L. (2015). Spatial Variation in the Healthy Human Lung Microbiome and the Adapted Island Model of Lung Biogeography. Ann. Am. Thorac. Soc..

[25] Dickson R.P., Erb-Downward J.R., Huffnagle G.B. (2014). Towards an ecology of the lung: New conceptual models of pulmonary microbiology and pneumonia pathogenesis. Lancet Respir. Med..

[26] Invernizzi R., Wu B.G., Barnett J., Ghai P., Kingston S., Hewitt R.J., Feary J., Li Y., Chua F., Wu Z. (2021). The Respiratory Microbiome in Chronic Hypersensitivity Pneumonitis Is Distinct from That of Idiopathic Pulmonary Fibrosis. Am. J. Respir. Crit. Care Med..

[27] Li J., Hu Y., Liu L., Wang Q., Zeng J., Chen C. (2020). PM2.5 exposure perturbs lung microbiome and its metabolic profile in mice. Sci. Total Environ..

[28] Pang J., Qi X., Luo Y., Li X., Shu T., Li B., Song M., Liu Y., Wei D., Chen J. (2021). Multi-omics study of silicosis reveals the potential therapeutic targets PGD(2) and TXA(2). Theranostics.

[29] Bruhat A., Chérasse Y., Chaveroux C., Maurin A.C., Jousse C., Fafournoux P. (2009). Amino acids as regulators of gene expression in mammals: Molecular mechanisms. Biofactors.

[30] Meng Y., Bo Z., Feng X., Yang X., Handford P.A. (2024). The Notch Signaling Pathway: Mechanistic Insights in Health and Disease. Engineering.

[31] Bargagli E., Refini R.M., d’Alessandro M., Bergantini L., Cameli P., Vantaggiato L., Bini L., Landi C. (2020). Metabolic Dysregulation in Idiopathic Pulmonary Fibrosis. Int. J. Mol. Sci..

[32] Dong L., Dong W., Ren Y., Xu C., Wang X., Sun P., Meng Y., Li C., Li G., Jiang J. (2026). Machine Learning-Enabled Insights: Dihydromyricetin’s Novel Role in Inhibiting the TGF-β/ALK5 Signaling Cascade for the Treatment of Pulmonary Fibrosis. Engineering.

[33] Luo K. (2017). Signaling Cross Talk between TGF-β/Smad and Other Signaling Pathways. Cold Spring Harb. Perspect. Biol..

[34] Yang X., Li C., Wang X., Zheng Z., Sun P., Xu C., Chen L., Jiang J., Normark S., Henriques-Normark B. (2024). An Update on the Clinical Pipelines of New Antibacterial Drugs Developed in China. Engineering.

