# Supplementary material for: Integrated Analysis of the Lung Microbiome and Metabolome Reveals Associations Between Amino Acid Metabolism and Pulmonary Fibrosis in a Bleomycin-Induced Mouse Model
Source: Int J Mol Sci. 2026 Jun 30;27(13):5895. doi: 10.3390/ijms27135895 (PMC13362081; doi:10.3390/ijms27135895)
Supplement: Supplementary file 1 [file ijms-27-05895-s001.zip › result/1.MetQuant-QC/1-MetQuant-QC-readme.pdf]

MetQuant-QC Readme

|-- 1.MetQuant-QC【代谢物定性定量结果目录】

| |-- meta_intensity_{all}.{xls,xlsx} 【所有代谢物定性定量表】

| |-- Class_stat_{all}.{xls} 【代谢物的一级分类统计表】

| |-- Pie_Chart_{all}.{png,pdf} 【代谢物一级分类饼图】

| |-- sam_qc_infor_{all}.xls 【样本信息及总 PCA 图中样本编号信息】

| |-- Samples_QC_{all}-PCA[.3D].{png,pdf} 【QC 及所有代谢样本的总 PCA 图】

| |-- Samples_QC_pcaloading_{all}.{png,pdf} 【QC 及所有代谢样本总 PCA 图的载荷图】

| |-- Samples_{all}-PCA[.3D].{png,pdf} 【所有代谢样本的总 PCA 图】

| |-- Samples_pcaloading_{all}.{png,pdf} 【所有代谢样本总 PCA 图的载荷图】

| |-- **【TIC】**

| |-- {all} 【不同离子模式TIC图目录】

| | |-- *TIC.pdf 【QC 及样本的 TIC 图】

| |-- **【Correlation】**

| |-- cor_pearson_{all}.{png,pdf,xls} 【QC 样本相关性分析结果】

meta_intensity_{all}.{xls,xlsx } 【所有代谢物定性定量表】

第一列： Compound_ID，代谢物 ID（该 ID 是为了方便检索及后续分析而随机添加的编号， 无实际意义）；

第二、三列： Name、ChineseName，代谢物的中英文名称（代谢物中文描述为机翻仅供参考）；

第四列：IonMode，采集模式，P表示正模式采集，N表示负模式采集；

第五列： Formula，代谢物的分子式；

第六列： Molecular Weight，分子量；

第七列：m/z，质荷比；

第八列：MassError，同一物质母离子实测值和理论值的偏差；

第九列：Adduct，加和离子形式；

第十列：RT[min]，保留时间；

第十一列： Score，定性打分值；

第十二列： Level 1，样本中的代谢物与数据库在MS1、MS2和RT都匹配；Level 2，样本中的代谢物与数据库MS1和MS2都匹配；Level3，样本中的代谢物与数据库MS1匹配；

第十三列：Column，色谱柱类型；

第十四列~第十九列：ClassI & ClassI (Chinese)、ClassII & ClassII (Chinese)、ClassIII & ClassIII (Chinese)，代谢物三级分类的中英文信息；

第二十列：CAS，物质CAS号；

第二十一列~第 二十五列：HMDB_ID、KEGG_ID、Lipidmaps_ID、PubChemID以及KEGG_MapID，分别为HMDB、KEGG、Lipidmaps、PubChemID 数据库编号以及KEGG数据库通路编号；

第二十六列~第二十七列：SMILES、InChIKey，源于PubChem数据库中，SMILES是用单行文本表达化合物的结构，InChIKey表示固定长度为25个字符的分子表示形式；

第二十八列~：代谢物在各个样本中的相对定量信息（峰面积值）；

Class_stat_{all}.xls

第一列：代谢物数量

第二列：代谢物一级分类信息

sam_qc_infor_{all}.xls

第一列： Sample name ，样本名称；

第二列： Batch，上机批次；

第三列： Group name，组别名称；

第四列： ID num，样本名称顺序（总样本 PCA 图对应的编号）；

cor_pearson_{all}.{png,pdf,xls}

cor_pearson_{all}.xls QC 样本两两相关性|r| 的值

cor_pearson_{all}.{png,pdf }

横坐标、纵坐标分别为 QC 样本， QC 样本两两相关性 |r| 的值越接近于 1，表明 QC 的相关性越好，说明整个检测过程稳定性越好。

Samples_QC_{all}-PCA[.3D].{png,pdf}

实验样本+QC 样本的 PCA 分析：横坐标 PC1 和纵坐标 PC2 分别表示排名第一和第二的主成分的得分，不同颜色的散点表示不同实验分组的样本，椭圆为 95%的置信区间，QC 越聚集说明检测过程稳定性越好。

Samples_QC_pcaloading_{all}.{png,pdf}

样本比较对 PCA loading 分析载荷图：载荷图（loading plot）的横坐标代表每个物质在第一主成分上的载荷大小（cosα），纵坐标代表每个物质在第二主成分上的载荷大小（cosβ)。载荷图本质上描述的是构成第一主成份和第二主成份的线性方程的系数，载荷的绝对值越大，对于主成份的影响就越大。

Samples_{all}-PCA[.3D].{png,pdf}

实验样本 PCA 分析：横坐标 PC1 和纵坐标 PC2 分别表示排名第一和第二的主成分的得分，不同颜色的散点表示不同实验分组的样本，椭圆为 95%的置信区间，除 QC 样本外，考察实验样本组内聚集及组间的离散情况。

***TIC.pdf**

为 QC 样本、实验样本的 TIC 图
