# Supplementary material for: Integrated Analysis of the Lung Microbiome and Metabolome Reveals Associations Between Amino Acid Metabolism and Pulmonary Fibrosis in a Bleomycin-Induced Mouse Model
Source: Int J Mol Sci. 2026 Jun 30;27(13):5895. doi: 10.3390/ijms27135895 (PMC13362081; doi:10.3390/ijms27135895)
Supplement: Supplementary file 1 [file ijms-27-05895-s001.zip › result/2.MetAnnotation/2-MetAnnotation-readme.pdf]

MetAnnotation Readme

|-- 2. MetAnnotation 【代谢物注释结果目录】

| -- **KEGG** 【基于 KEGG 数据库注释代谢物通路结果】

| |--meta_{all}_kegg_anno_category.xls 【KEGG 通路注释结果】

| |--meta_{all}.KEGG.Anno.{png,pdf} 【KEGG 注释结果统计图】

| |---meta_{all}.KEGG.anno.{xls,xlsx} 【代谢物 KEGG 注释结果列表】

| -- **HMDB** 【基于 HMDB 数据库注释代谢物分类结果】

| |--meta_{all}_hmdb_anno_category.xls 【HMDB 分类注释结果】

| |--meta_{all}.HMDB.Anno.{png,pdf} 【HMDB 分类注释结果统计图】

| |-- meta_{all}.HMDB.anno.{xls,xlsx} 【代谢物 HMDB 注释结果列表】

| -- **Lipidmaps** 【基于 Lipidmaps 数据库注释代谢物分类结果】

| |--meta_{all}_lipidmaps_anno_category.xls【Lipidmaps 分类注释结果】

| |--meta_{all}.Lipidmaps.Anno.{png,pdf} 【Lipidmaps 分类注释结果统计图】

| |-- meta_{all}.Lipidmaps.anno.{xls,xlsx} 【代谢物 Lipidmaps 注释结果列表】

| -- **HMDB_KEGG_Lipidmaps** 【基于 KEGG、HMDB、Lipidmaps 数据库注释整合结果】

| -- meta_intensity _{all}_hmdb_kegg_lipidmaps.{xls,xlsx} 【总 HMDB、KEGG、Lipimaps 数据库注释整合结果】

KEGG

meta_{all}_kegg_anno_category.xls

第一列： KO_Pathway_Level1，KEGG 数据库相应 pathway 的第一层级名称；

第二列： KO_Pathway_Level2，KEGG 数据库相应 pathway 的第二层级名称；

第三列： Meta_Num，对应第二层级中注释到的代谢物数目；

第四列： Metabolites，代谢物 ID

meta_{all}.KEGG.Anno. {png,pdf}

横坐标代表代谢物数目，纵坐标代表注释到的 KEGG 通路；该图展示的是 pathway 一级分类各二级分类对应的代谢物数目。

meta_{all}_kegg_anno.{xls,xlsx}

第一列： Compound_ID，代谢物 ID；

第二列： Name，代谢物英文名称；

第三列： Kegg_ID，注释到的 kegg 数据库中对应的 ID；

第四列： Kegg_name，代谢物在 kegg 数据库中对应的名称；

第五列： formula，代谢物分子式；

第六列： Kegg_map，代谢通路对应的 mapID 和名称；

HMDB

meta_{all}_hmdb_anno_category.xls

第一列： SuperClass，HMDB 中的二级分类，如： 有机酸、脂类等类别；

第二列： Metabolites_nums，该分类注释到的代谢物数目；

第三列： Metabolites，代谢物 ID；

第四列： Metabolite_Descriptions，代谢物在 HMDB 数据库中的描述。

meta_{all}.HMDB.Anno.{png,pdf}

横坐标代表代谢物的数目，纵坐标代表注释到的 HMDB 分类；该图展示 HMDB 中二级分类(SuperClass)对应的代谢物数目。

meta_{all}_hmdb_anno.{xls,xlsx}

第一列： Compound_ID，代谢物 ID；

第二列： Name，代谢物英文名称；

第三列： Formula，代谢物分子式；

第四列： HMDB_ID，代谢物在 HMDB 数据库中对应的 ID；

第五列： tax_Kingdom，第一层级分类；

第六列： tax_SuperClass，第二层级分类；

第七列： tax_Class，第三层级分类；

第八列： tax_SubClass，第四级分类；

第九列： tax_DirectParent，化合物的化学类别；

第十列： Source，化合物来源；

Lipidmaps

meta_{all}_lipidmaps_anno_category.xls

第一列： CATEGORY，lipidmaps 分类信息（八大类脂质）；

第二列： MAIN_CLASS，八大类下的主层级分类；

第三列： Metabolites_nums，MAIN_CLASS 层级中注释到的代谢物数目；

第四列： Metabolites，代谢物 ID；

meta_{all}.Lipidmaps.Anno.{png,pdf}

横坐标代表代谢物数目，纵坐标代表注释到的 LIPID MAPS 脂质分类； 该图展示的是 LIPID MAPS 中 8 大脂质分类（Category）下的主层级分类（Main_Class）对应的（脂质）代谢物数目。

meta_{all}_lipidmaps_anno.{xls,xlsx}

第一列： Compound_ID，代谢物 ID；

第二列： Name，代谢物英文名称；

第三列： Formula，代谢物分子式；

第四列： Lipidmaps_ID，Lipidmaps 数据库中对应的 ID；

第五列： COMMON_NAME，常用名；

第六列： SYSTEMATIC_NAME，系统命名；

第七列： SYNONYMS，化合物同义名称；

第八列：CATEGORY，分类信息（八大类脂质）；

第九列： MAIN_CLASS，八大类下的主层级分类；

第十列： SUB_CLASS，主层级分类下的子类；

meta_intensity _{all}_hmdb_kegg_lipidmaps.{xls,xlsx}

第一列： Compound_ID，代谢物ID；

第二、三列：
Name、ChineseName，代谢物的中英文名称（代谢物中文描述为机翻仅供参考）；

第四列：IonMode，采集模式，P表示正模式采集，N表示负模式采集；

第五列： Formula，代谢物的分子式；

第六列： Molecular Weight，分子量；

第七列：m/z，质荷比；

第八列：MassError，同一物质母离子实测值和理论值的偏差；

第九列：Adduct，加和离子形式；
第十列：RT[min]，保留时间；
第十一列： Score，定性打分值；
第十二列： Level，鉴定等级；Level
1，样本中的代谢物与数据库在MS1、MS2和RT都匹配；Level
2，样本中的代谢物与数据库MS1和MS2都匹配；Level 3，样本中的代谢物与数据库MS1匹配；

第十三列：Column，色谱柱类型；
第十四列~第十九列：ClassI & ClassI (Chinese)、ClassII & ClassII (Chinese)、ClassIII & ClassIII (Chinese)，代谢物三级分类的中英文信息；
第二十列：CAS，物质CAS号；
第二十一列~第二十四：HMDB_ID、SuperClass(HMDB)、Class(HMDB)、SubClass(HMDB)为代谢物的HMDB数据库中对应的ID以及三级分类信息

第二十五列~第二十七列： Other_name(Kegg_name)、KEGG_ID、KEGG_pathway分别名，KEGG数据中物质的别名，代谢物在KEGG数据库中的ID以及代谢通路ID;

第二十八列~第三十一列：Lipidmaps_ID、CATEGORY(Lipidmaps)、MAIN_CLASS(Lipidmaps)、SUB_CLASS(Lipidmaps)分别代谢物在Lipidmaps数据库中的ID以及其三级分类；

第三十二列：PubChemID，代谢物在PubChem数据库的ID

第三十三列、第三十四列：SMILES、InChIKey，源于PubChem数据库中，SMILES是用单行文本表达化合物的结构，InChIKey表示固定长度为25个字符的分子表示形式；
第三十五列~：代谢物在各个样本中的相对定量信息（峰面积值）；
