# Supplementary material for: Integrated Analysis of the Lung Microbiome and Metabolome Reveals Associations Between Amino Acid Metabolism and Pulmonary Fibrosis in a Bleomycin-Induced Mouse Model
Source: Int J Mol Sci. 2026 Jun 30;27(13):5895. doi: 10.3390/ijms27135895 (PMC13362081; doi:10.3390/ijms27135895)
Supplement: Supplementary file 1 [file ijms-27-05895-s001.zip › result/2.MetAnnotation/HMDB/meta_all.HMDB.Anno.pdf]

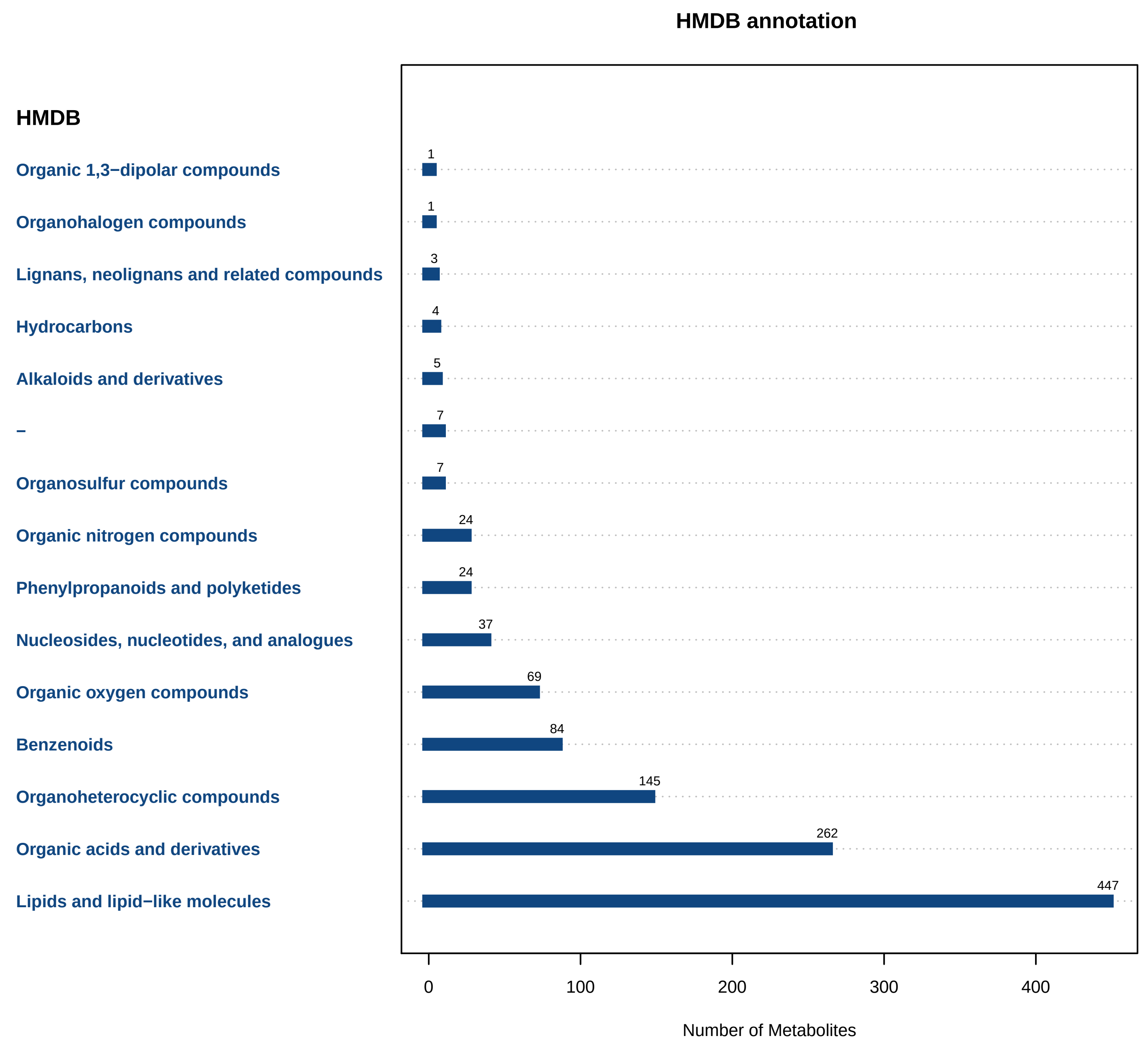
HMDB annotation
HMDB
Organic 1,3−dipolar compounds
Organohalogen compounds
Lignans, neolignans and related compounds
Hydrocarbons
Alkaloids and derivatives
–
Organosulfur compounds
Organic nitrogen compounds
Phenylpropanoids and polyketides
Nucleosides, nucleotides, and analogues
Organic oxygen compounds
Benzenoids
Organoheterocyclic compounds
Organic acids and derivatives
Lipids and lipid−like molecules
1
1
3
4
5
7
7
24
24
37
69
84
145
262
447
0
100
200
300
400
Number of Metabolites
