# Supplementary material for: Integrated Analysis of the Lung Microbiome and Metabolome Reveals Associations Between Amino Acid Metabolism and Pulmonary Fibrosis in a Bleomycin-Induced Mouse Model
Source: Int J Mol Sci. 2026 Jun 30;27(13):5895. doi: 10.3390/ijms27135895 (PMC13362081; doi:10.3390/ijms27135895)
Supplement: Supplementary file 1 [file ijms-27-05895-s001.zip › result/2.MetAnnotation/Lipidmaps/meta_all.Lipidmaps.Anno.pdf]

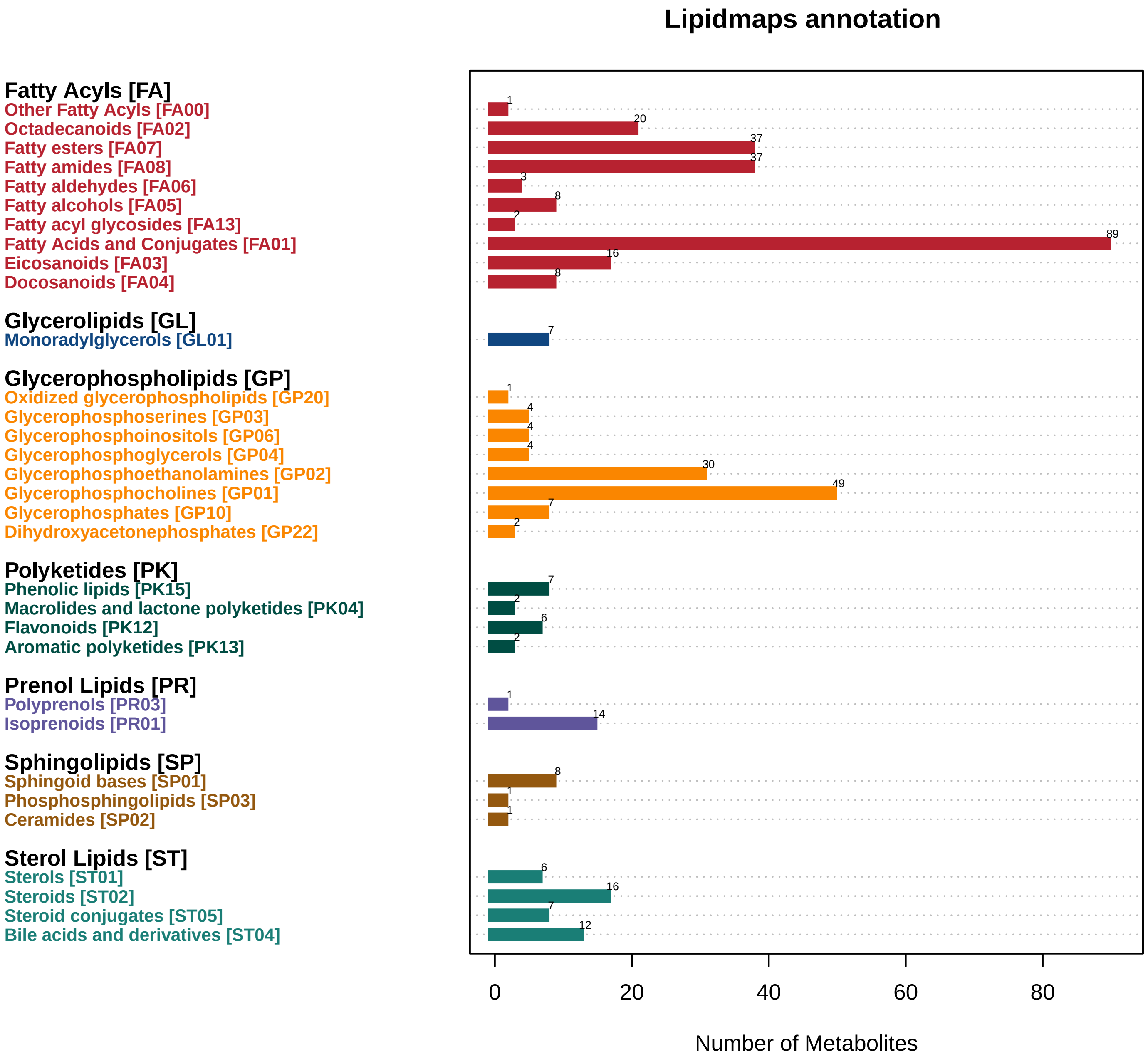
Lipidmaps annotation
Fatty Acyls [FA]
Other Fatty Acyls [FA00]
Octadecanoids [FA02]
Fatty esters [FA07]
Fatty amides [FA08]
Fatty aldehydes [FA06]
Fatty alcohols [FA05]
Fatty acyl glycosides [FA13]
Fatty Acids and Conjugates [FA01]
Eicosanoids [FA03]
Docosanoids [FA04]
Glycerolipids [GL]
Monoradylglycerols [GL01]
Glycerophospholipids [GP]
Oxidized glycerophospholipids [GP20]
Glycerophosphoserines [GP03]
Glycerophosphoinositols [GP06]
Glycerophosphoglycerols [GP04]
Glycerophosphoethanolamines [GP02]
Glycerophosphocholines [GP01]
Glycerophosphates [GP10]
Dihydroxyacetonephosphates [GP22]
Polyketides [PK]
Phenolic lipids [PK15]
Macrolides and lactone polyketides [PK04]
Flavonoids [PK12]
Aromatic polyketides [PK13]
Prenol Lipids [PR]
Polyprenols [PR03]
Isoprenoids [PR01]
Sphingolipids [SP]
Sphingoid bases [SP01]
Phosphosphingolipids [SP03]
Ceramides [SP02]
Sterol Lipids [ST]
Sterols [ST01]
Steroids [ST02]
Steroid conjugates [ST05]
Bile acids and derivatives [ST04]
1
20
37
37
3
8
2
89
16
8
7
1
4
4
4
30
49
7
2
7
2
6
2
1
14
8
1
1
6
16
7
12
0
20
40
60
80
Number of Metabolites
