# Supplementary material for: Integrated Analysis of the Lung Microbiome and Metabolome Reveals Associations Between Amino Acid Metabolism and Pulmonary Fibrosis in a Bleomycin-Induced Mouse Model
Source: Int J Mol Sci. 2026 Jun 30;27(13):5895. doi: 10.3390/ijms27135895 (PMC13362081; doi:10.3390/ijms27135895)
Supplement: Supplementary file 1 [file ijms-27-05895-s001.zip › result/3.MetDiffScreening/3-MetDiffScreening-readme.pdf]

MetDiffScreening Readme

|-- 3.MetDiffScreening 【差异代谢物筛选结果目录】

| -- *.vs.* 【样本比较对目录】

| |-- *.vs.*_{all}.{xls,xlsx} 【样本比较对定量分析结果】

| |-- *.vs.*_{all}_Diff_order.{xls,xlsx} 【样本比较对差异代谢物分析】

| |-- *.vs.*_{all}_diff.anno.{xls,xlsx} 【样本比较对差异代谢物注释结果】

| |-- *.vs.*_{all}-PCA[.3D].{png,pdf} 【样本比较对 PCA 分析】

| |-- *.vs.*_{all}-PCA-pcaloading.{png,pdf} 【样本比较对 PCA loading 分析】

| |-- *.vs.*_{all}-PLSDA-{score,valid}.{png,pdf}【比较对 PLSDA 分析】

| |-- *.vs.*_{all}-PLSDA-loading.{png,pdf}【比较对 PLSDA loading 分析】

| |-- *.vs.*_{all}_stem.{png,pdf}【比较对火柴杆图分析】

| |-- *.vs.*_{all}.xls.volcano.{png,pdf} 【样本比较对火山图分析】

***.vs.*_{all}.{xls,xlsx}**

第一列： Compound_ID，代谢物 ID（该 ID 是为了方便检索及后续分析而随机添加的编号， 无实际意义）；

第二、三列：Name、ChineseName，代谢物的中英文名称（代谢物中文描述为机翻仅供参考）；

第四列：IonMode，采集模式，P表示正模式采集，N表示负模式采集；

第五列： Formula，代谢物的分子式；

第六列： Molecular Weight，分子量；

第七列：m/z，质荷比；

第八列：MassError，同一物质母离子实测值和理论值的偏差；

第九列：Adduct，加和离子形式；

第十列：RT[min]，保留时间；

第十一列： Score，定性打分值；

第十二列： Level，鉴定等级；Level 1，样本中的代谢物与数据库在MS1、MS2和RT都匹配；Level 2，样本中的代谢物与数据库MS1和MS2都匹配；Level 3，样本中的代谢物与数据库MS1匹配；

第十三列：Column，色谱柱类型；

第十四列~第十九列：ClassI & ClassI (Chinese)、ClassII & ClassII (Chinese)、ClassIII & ClassIII (Chinese)，代谢物三级分类的中英文信息；

第二十列：CAS，物质CAS号；

第二十一列~第二十五列：HMDB_ID、KEGG_ID、Lipidmaps_ID、PubChemID以及KEGG_MapID，分别为HMDB、KEGG、Lipidmaps、PubChemID 数据库编号以及KEGG数据库通路编号；

第二十六列~第二十七列：SMILES、InChIKey，源于PubChem数据库中，SMILES是用单行文本表达化合物的结构，InChIKey表示固定长度为25个字符的分子表示形式；

第二十八列-倒数第六列：不同样品定量值；

倒数第五列： FC，比较对差异倍数；

倒数第四列： log2FC，比较对差异倍数的 log2 值；

倒数第三列： Pvalue，比较对显著性 p-value；

倒数第二列： ROC，受试者工作特征曲线面积 AUC 值；

倒数第一列： VIP，变量重要性投影, 来反映每一个样品定量值对差异的贡献程度，一般设置 VIP>1；

***.vs.*_{all}_Diff_order.{xls,xlsx}**

第一列： Compound_ID，代谢物 ID（该 ID 是为了方便检索及后续分析而随机添加的编号， 无实际意义）；

第二、三列：Name、ChineseName，代谢物的中英文名称（代谢物中文描述为机翻仅供参考）；

第四列：IonMode，采集模式，P表示正模式采集，N表示负模式采集；

第五列： Formula，代谢物的分子式；
第六列： Molecular Weight，分子量；
第七列：m/z，质荷比；
第八列：MassError，同一物质母离子实测值和理论值的偏差；
第九列：Adduct，加和离子形式；
第十列：RT[min]，保留时间；
第十一列： Score，定性打分值；
第十二列： Level，鉴定等级；Level 1，样本中的代谢物与数据库在MS1、MS2和RT都匹配；Level 2，样本中的代谢物与数据库MS1和MS2都匹配；Level 3，样本中的代谢物与数据库MS1匹配；
第十三列：Column，色谱柱类型；
第十四列~第十九列：ClassI & ClassI (Chinese)、ClassII & ClassII (Chinese)、ClassIII & ClassIII (Chinese)，代谢物三级分类的中英文信息；
第二十列：CAS，物质CAS号；
第二十一列~第二十五列：HMDB_ID、KEGG_ID、Lipidmaps_ID、PubChemID以及KEGG_MapID，分别为HMDB、KEGG、Lipidmaps、PubChemID 数据库编号以及KEGG数据库通路编号；
第二十六列~第二十七列：SMILES、InChIKey，源于PubChem数据库中，SMILES是用单行文本表达化合物的结构，InChIKey表示固定长度为25个字符的分子表示形式；

第二十八列： FC，比较对差异倍数；

第二十九列： log2FC，比较对差异倍数的 log2 值；

第三十列： Pvalue，比较对显著性 p-value；

第三十一列： ROC，受试者工作特征曲线面积 AUC 值；

第三十二列： VIP，变量重要性投影, 来反映每一个样品定量值对差异的贡献程度， 一般设置 VIP>1；

第三十三列： Up.Down，上调（up）或下调（down）；

第三十四列~倒数第一列：不同样品定量值；

***.vs.*_{all}_diff.anno.{xls,xlsx}**

第一列~第 二十列：同*.vs.*_ {all}_Diff_order.{xls,xlsx}表中的第一列~第 二十列

第二十一列~第二十四：HMDB_ID、SuperClass(HMDB)、Class(HMDB)、SubClass(HMDB)为代谢物的HMDB数据库中对应的ID以及三级分类信息

第二十五列~第二十七列： Other_name(Kegg_name)、KEGG_ID、KEGG_pathway分别名，KEGG数据中物质的别名，代谢物在KEGG数据库中的ID以及代谢通路ID;

第二十八列~第三十一列：Lipidmaps_ID、CATEGORY(Lipidmaps)、MAIN_CLASS(Lipidmaps)、SUB_CLASS(Lipidmaps)分别代谢物在Lipidmaps数据库中的ID以及其三级分类；

第三十二列：PubChemID，代谢物在PubChem数据库的ID

第三十三列、第三十四列：SMILES、InChIKey，源于PubChem数据库中，SMILES是用单行文本表达化合物的结

构，InChIKey表示固定长度为25个字符的分子表示形式；
第三十五列：FC，比较对差异倍数；
第三十六列：log2FC，比较对差异倍数的 log2 值；

第三十七列：Pvalue，比较对显著性 p-value；
第三十八列：ROC，受试者工作特征曲线面积 AUC 值；
第三十九列：VIP，变量重要性投影, 来反映每一个样品定量值对差异的贡献程度，一般设置 VIP>1；
第四十列：Up.Down，上调（up）或下调（down）；
第四十一列~倒数第一列：不同样品定量值；

***.vs.*_{all}-PCA[.3D].{png,pdf}**

样本比较对 PCA 分析[3D]图：通过 PCA 的方法，观察两组样本间的总体分布趋势。横坐标 PC1 和纵坐标 PC2 分别表示排名第一和第二的主成分的得分，不同颜色的散点表示不同实验分组的样本，椭圆为 95%的置信区间（生物学重复数目小于 4 时，无法展示 95%的置信椭圆）。

***.vs.*_{all}-pcaloading.{png,pdf}**

样本比较对 PCA loading 分析载荷图：载荷图（loading plot）的横坐标代表每个物质在第一主成分上的载荷大小（cosα）, 纵坐标代表每个物质在第二主成分上的载荷大小（cosβ）。载荷图本质上描述的是构成第一主成份和第二主成份的线性方程的系数，载荷的绝对值越大，对于主成份的影响就越大。

***.vs.*_{all}-PLSDA-score.{png,pdf}**

比较对 PLS-DA 得分散点图：运用偏最小二乘法回归建立代谢物表达量与样品类别之间的关系模型，来实现对样品类别的预测。横坐标为样本在第一主成分上的得分；纵坐标为样本在第二主成分上的得分；R2Y 表示模型的解释率，Q2Y 用于评价 PLS-DA 模型的预测能力，且 R2Y 大于 Q2Y 时表示模型建立良好。

***.vs.*_{all}-PLSDA-valid.{png,pdf}**

比较对 PLS-DA 排序检验图：对模型进行排序检验，用以判别模型质量好坏及模型是否“过拟合”。横坐标代表随机分组的 Y 与原始分组 Y 的相关性，纵坐标代表 R2 和 Q2 的得分；图中一个点表示一次检验。

***.vs.*_{all}-PLSDA- loading.{png,pdf}**

样本比较对 PCA loading 分析载荷图：载荷图（loading plot）的横坐标代表每个物质在第一主成分上的载荷大小（cosα）, 纵坐标代表每个物质在第二主成分上的载荷大小（cosβ）。载荷图本质上描述的是构成第一主成份和第二主成份的线性方程的系数，载荷的绝对值越大，对于主成份的影响就越大。

***.vs.*_{all}_stem.{png,pdf}**

样本比较对火柴杆图：火柴杆图的横坐标代表 log2FC 值，纵坐标代表代谢物，图中点的大小代表 VIP 值，红色表示显著上调，蓝色表示显著下调。

***.vs.*_{all}.xls.volcano.{png,pdf}**

样本比较对火山图：根据比较对间的差异代谢物的 VIP 值、P 值、FC 值绘制火山图，可直观显示差异代谢物的整体分布情况。横坐标表示代谢物在不同分组中的表达倍数变化(log2FC)，纵坐标表示差异显著性水平(-log10(p-value))，图中每个点代表一个代谢物，点的大小代表 VIP 值，显著上调的代谢物用红色点表示，显著下调的代谢物用绿色点表示。
