# Supplementary material for: Integrated Analysis of the Lung Microbiome and Metabolome Reveals Associations Between Amino Acid Metabolism and Pulmonary Fibrosis in a Bleomycin-Induced Mouse Model
Source: Int J Mol Sci. 2026 Jun 30;27(13):5895. doi: 10.3390/ijms27135895 (PMC13362081; doi:10.3390/ijms27135895)
Supplement: Supplementary file 1 [file ijms-27-05895-s001.zip › result/4.MetDiffAnalysis/4-MetDiffAnalysis-readme.pdf]

MetDiffAnalysis Readme

|-- 4.MetDiffAnalysis 【差异代谢物分析结果目录】
| -- Box_diff【总差异代谢物箱线图目录】
| |-- {all}【不同离子模式箱线图目录】
| | |--*_{all}_Box_plot.{png,pdf} 【不同组间差异代谢物箱线图】
| -- Violin_diff【总差异代谢物小提琴图目录】
| |-- {all}【不同离子模式小提琴图目录】
| | |--*_{all}_Violin_plot.{png,pdf} 【不同组间差异代谢小提琴图】
| -- Heatmap_diff 【总差异代谢物热图目录。注：仅一个比较对无此目录和对应结果】
| |--Diff_Heatmap_{all}{_cluster,cluster_detail}.{png,pdf}【总差异代谢物聚类热图】
| |--Diff_Heatmap_ {all}{,_detail}.{png,pdf}【总差异代谢物不聚类热图】
| |--Diff_Heatmap_Name_{all}.{xls,xlsx} 【总差异代谢物热图分析列表】
| |--Diff_Heatmap_ {all}.heatmap_scale.{xls,xlsx}【总差异代谢物热图 scale 转换后列表】
| -- Kmeans_diff 【总差异代谢物 Kmeans 分析目录】
| |-- {all}【不同离子模式 Kmeans 分析目录】
| | |--Diff_kmeans_subcluster_{all}.{xls,xlsx}【总差异代谢物 Kmeans 分析列表】
| | |--Diff_kmeans_subcluster【总差异代谢物 Kmeans 分析目录】
| | | |--subcluster_*.{xls}【Kmeans 分析中每个 subcluster 分析表格】
| | | |--kmeans_subcluster_plots.{png,pdf}【Kmeans 分析结果图】
| -- Venn_diff【基于比较对的差异代谢物 venn 图目录】
| |--Diff_Venn_{all}.{png,svg} 【差异代谢物 venn 图】
| |--Diff_Venn_{Intersection, Union}_{all}_addinfo.{xls,xlsx} 【差异代谢物 venn 图列表】
| -- *.vs.* 【比较的样本对目录】
| |-- *.vs.*_{all}.corr.{xls,xlsx} 【差异代谢物相关性列表】
| |-- *.vs.*_{all}_Pvalue.{xls,xlsx} 【基于相关性分析的 p 值列表】
| |-- *.vs.*_{all}_zscore.{xls,xlsx} 【差异代谢物 zscore 分析列表】
| |-- *.vs.*_{all}_cluster_heatmap[,_detail].{png,pdf} 【差异代谢物聚类热图】
| |-- *.vs.*_{all}_heatmap[._detail].{png,pdf} 【差异代谢物不聚类热图】
| |-- *.vs.*_{all}_corr.{png,pdf} 【差异代谢物相关性分析图】
| |-- *.vs.*_{all}_zscore.{png,pdf} 【差异代谢物 z-score 图】
| |-- *.vs. *_{all}_chord. {png,pdf} 【差异代谢物和弦图】
| |-- ROC_{all} 【差异代谢物 ROC 曲线图目录】
| | |-- *_{all}_ROC.pdf 【差异代谢物 ROC 曲线图】

Diff_Venn_{Intersection, Union}_{all}_addinfo.{xls,xlsx}

第一列： Compound_ID，代谢 ID;
第二列： Name，代谢物名称;
*.vs. *列：该比较对包含该代谢物情况，1 代表包含，0 代表不包含;
*.vs. *_FC 列：该比较对中该代谢物的 FC 值;
*.vs. *_log2FC 列：该比较对中该代谢物的 FC 值取对数;
*.vs. *_Pvalue 列：该比较对中该代谢物的 P 值;

*.vs. *_ROC 列：该比较对中该代谢物受试者工作特征曲线面积 AUC 值；
*.vs. *_VIP 列：该比较对中该代谢物的 VIP 值；
*.vs. *_Up.Down 列：该比较对中该代谢物的差异上下调情况；

Diff_Heatmap_ Name_{all}.{xls,xlsx}

第一列： Compound_ID，代谢 ID；
第二列： Name，代谢物名称；
第三列-倒数第一列： 不同组别样本对应的代谢物均值；

Diff_Heatmap_{all}.heatmap_scale.{xls,xlsx}

第一列：代谢物名称；
第二列-倒数第一列： 代谢物均值经 scale 转换后的值；

Diff_kmeans_subcluster_{all}.{xls,xlsx}

第一列： subcluster 表示每个簇
第二列： Compound_ID，代谢 ID
第三列： Name，代谢物名称；
第四列-倒数第一列：不同组别样本对应的代谢物均值；

subcluster_*.{xls}

Kmeans 分析中每个 subcluster 分析表格；

***.vs.*_{all}_corr.{xls,xlsx}**

代谢物相关性分析表格；

***.vs.*_{all}_Pvalue.{xls,xlsx}**

基于代谢物相关性分析的 P 值；

***.vs.*_{all}_zscore.{xls,xlsx}**

代谢物 zscore 值表格；

***_{all}_Box_plot.{png,pdf}**

不同组间差异代谢物箱线图：对各比较对得到的差异代谢物取并集后，展示每一个代谢物在不同的样本组间的箱线图分析。

***_{all}_Violin_plot.{png,pdf}**

不同组间差异代谢物小提琴图：对各比较对得到的差异代谢物取并集后， 展示每一个代谢物在不同的样本组间的小提琴图分析。

Diff_Venn_{all}.{png,svg}

差异代谢物 venn 图：不同比较对之间共有和特有差异代谢物数量

Diff_Heatmap_{all}{cluster,cluster_detail}.{png,pdf}

总差异代谢物聚类热图：对各比较对之间的差异代谢物进行层次聚类分析，将差异代谢物相对定量值进行归一化转换并聚类。横向为代谢物的聚类，纵向为样本分组，聚类枝越短代表相似性越高。

Diff_Heatmap_{all}{,detail}.{png,pdf}

总差异代谢物不聚类热图：将差异代谢物相对定量值进行归一化转换。横向为代谢物的聚类，纵向为样本分组。

kmeans_subcluster_plots.{png,pdf}

Kmeans 分析结果图：横坐标表示样品组别名称，纵坐标为代谢物相对含量取对数中心化校正后的值。Cluster 代表相同变化趋势的代谢物为一个簇。 *metabolite(s)代表

该簇的代谢物的数目。每个子图中的灰色线条表示一个 cluster 中的代谢物在不同实验条件下相对校正后的值，蓝色线条表示这个 cluster 中的所有代谢物在不同实验条件下相对校正后的平均值。

***.vs.*_{all}_cluster_heatmap[,_detail].{png,pdf}**

差异代谢物聚类热图： 对两组样本获得的差异代谢物进行层次聚类分析， 得出同一比较对两组之间和组内代谢表达模式的差异情况。横向为代谢物的聚类， 纵向为样本类型， 聚类枝越短代表相似性越高。

***.vs.*_{all}_heatmap[,_detail].{png,pdf}**

差异代谢物不聚类热图：将两组样本获得的差异代谢物相对定量值进行归一化转换，得出同一比较对两组之间和组内代谢表达模式的差异情况。横向为代谢物的聚类，纵向为样本类型。

***.vs.*_{all}_corr.{png,pdf}**

差异代谢物相关性分析图：通过计算所有差异代谢物两两之间的皮尔逊相关系数，选取显著性水平 P-value 值从小到大排序的 Top20 的差异代谢物进行展示，可以查看代谢物与代谢物变化趋势的一致性。相关性最高为 1，为完全的正相关（红色），相关性最低为-1，为完全的负相关（蓝色），没有颜色的部分表示 P-value＞0.05。

***.vs.*_{all}_zscore.{png,pdf}**

差异代谢物 z-score 图： z-score （标准分数） 是基于代谢物的相对含量转换而来的值，用于衡量同一水平面上代谢物的相对含量的高低。横坐标为 z-score 值，纵坐标为差异代谢物，每个圆圈代表一个样本。图中只展示了 Top30（按 p-value 值从小到大排序）的代谢物 Z-score 值。 Z-score 超出 4 或-4 的样本无法展示。

***.vs. *_{all}_chord. {png,pdf}**

差异代谢物和弦图： 根据差异代谢物两两之间的皮尔逊相关系数，选取显著性水平 P-value 值从小到大排序的 Top20 的差异代谢物进行和弦图展示，每个点代表一个代谢物，点的大小代表代谢物 FC 值取对数值的大小，点的颜色代表代谢物的分类（NA 表示无分类信息） ，点之间的连线颜色代表代谢物间的相关性，蓝色代表负相关，红色代表正相关。

***_{all}_ROC.pdf**

差异代谢物 ROC 曲线图：ROC 曲线又叫受试者工作特征曲线或感受性曲线，根据一系列不同的二分类方式（分界值或决定域）绘制的曲线， 差异代谢物的 ROC 曲线可用来评判潜在的生物标记物。横坐标为假阳性率（1-特异度） ，纵坐标为真阳性率（灵敏度）。
