# Supplementary figures and images for: Integrated Analysis of the Lung Microbiome and Metabolome Reveals Associations Between Amino Acid Metabolism and Pulmonary Fibrosis in a Bleomycin-Induced Mouse Model (part 2 of 2)
Source: Int J Mol Sci. 2026 Jun 30;27(13):5895. doi: 10.3390/ijms27135895 (PMC13362081; doi:10.3390/ijms27135895)

B\_14d.vs.C\_14d

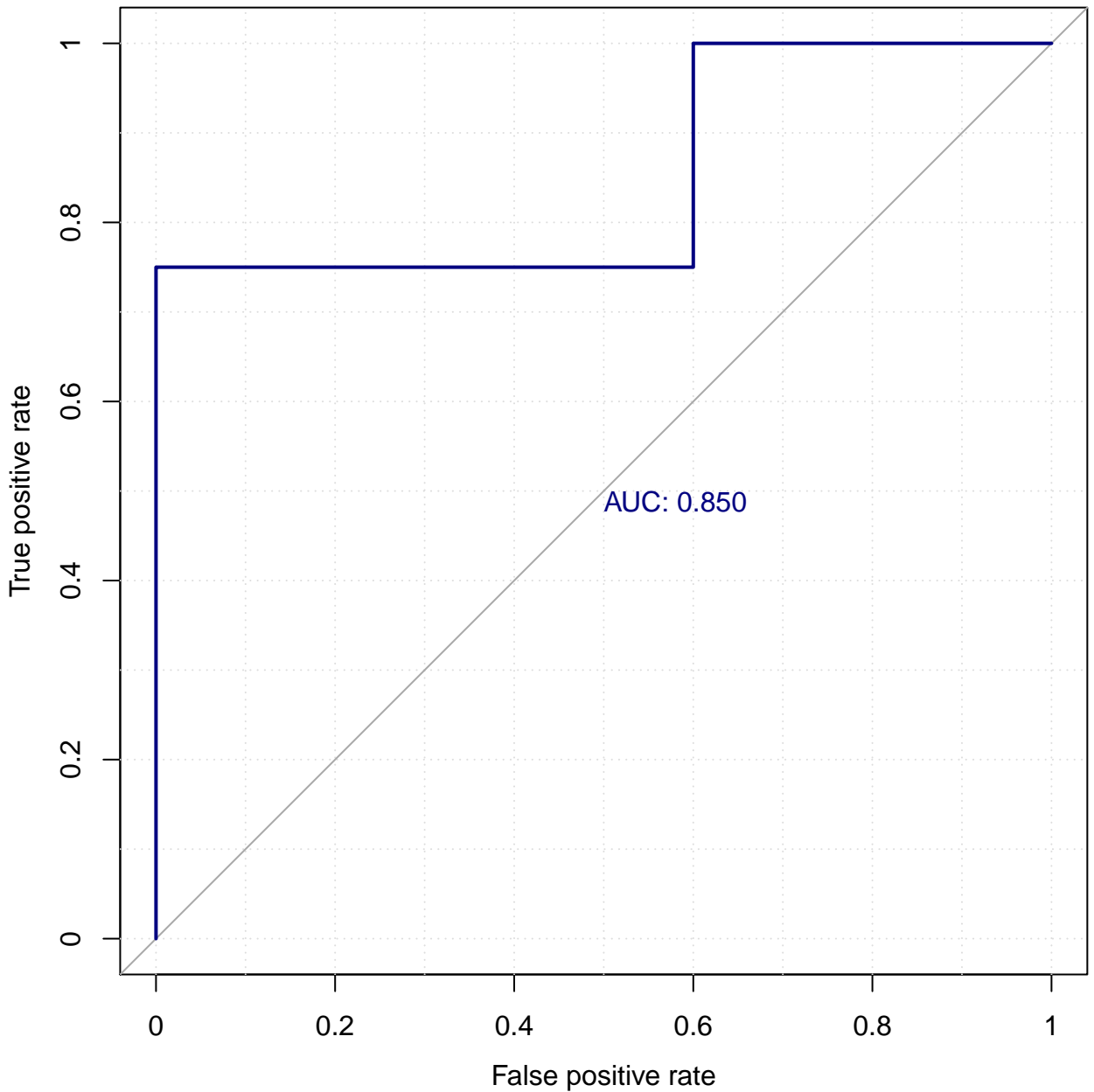

Supplement: Supplementary file 1 [file ijms-27-05895-s001.zip › result/4.MetDiffAnalysis/B_14d.vs.C_14d/ROC_all/Com_505_neg_ROC.pdf]

B\_14d.vs.C\_14d

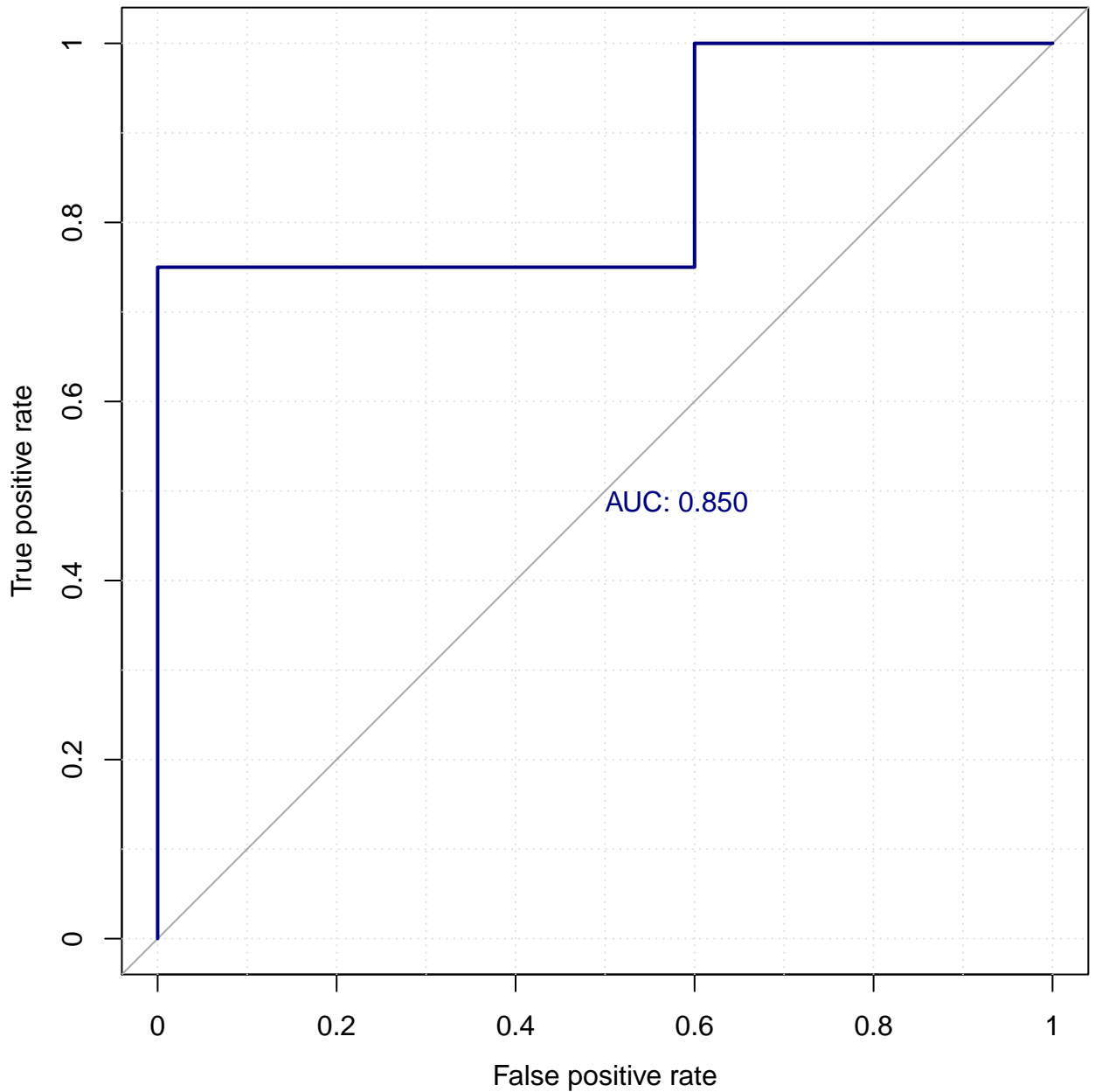

Supplement: Supplementary file 1 [file ijms-27-05895-s001.zip › result/4.MetDiffAnalysis/B_14d.vs.C_14d/ROC_all/Com_506_neg_ROC.pdf]

B\_14d.vs.C\_14d

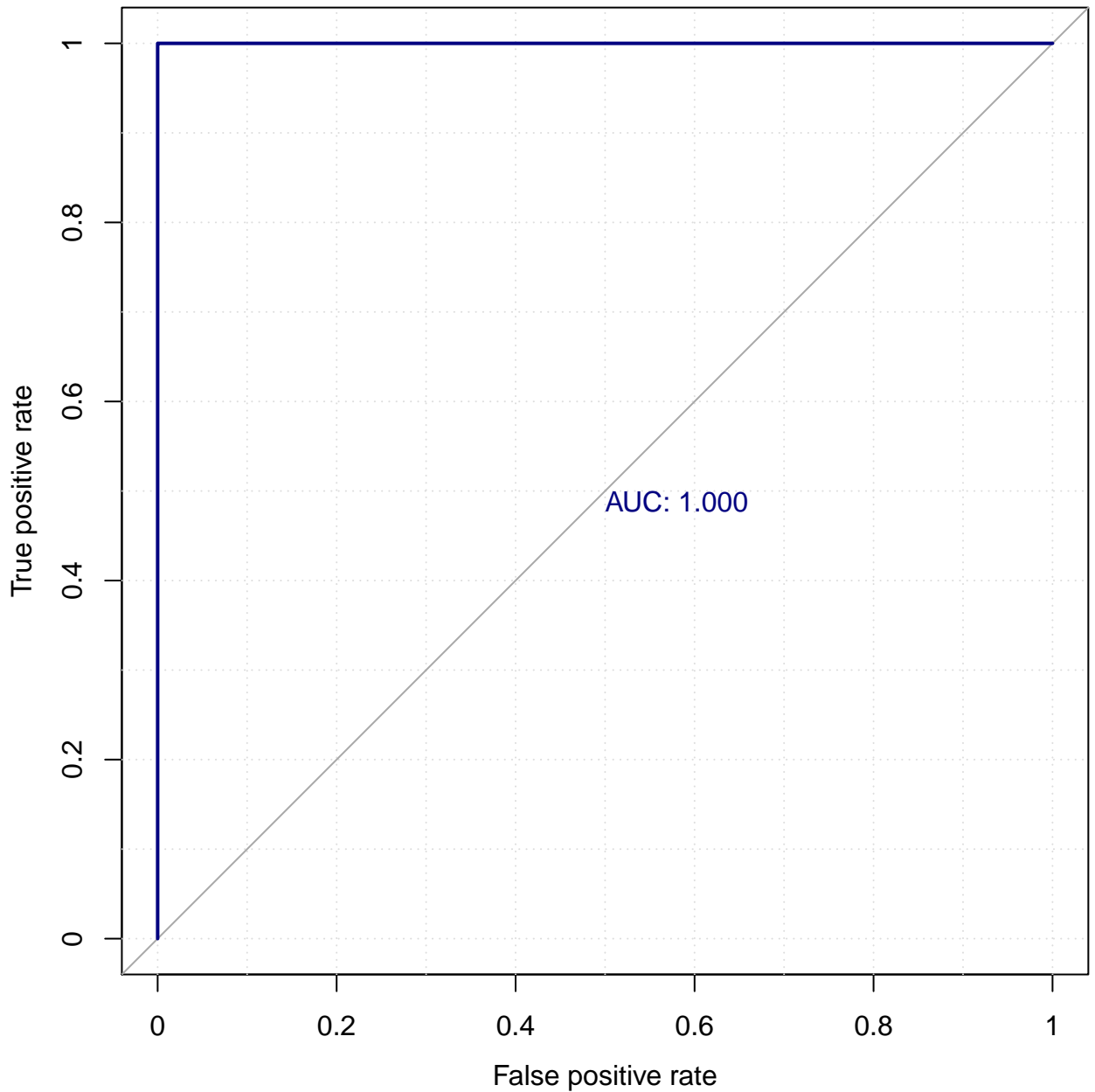

Supplement: Supplementary file 1 [file ijms-27-05895-s001.zip › result/4.MetDiffAnalysis/B_14d.vs.C_14d/ROC_all/Com_50_pos_ROC.pdf]
