# Supplementary figures and images for: Integrated Analysis of the Lung Microbiome and Metabolome Reveals Associations Between Amino Acid Metabolism and Pulmonary Fibrosis in a Bleomycin-Induced Mouse Model (part 1 of 2)
Source: Int J Mol Sci. 2026 Jun 30;27(13):5895. doi: 10.3390/ijms27135895 (PMC13362081; doi:10.3390/ijms27135895)

Pearson correlation between all QC samples

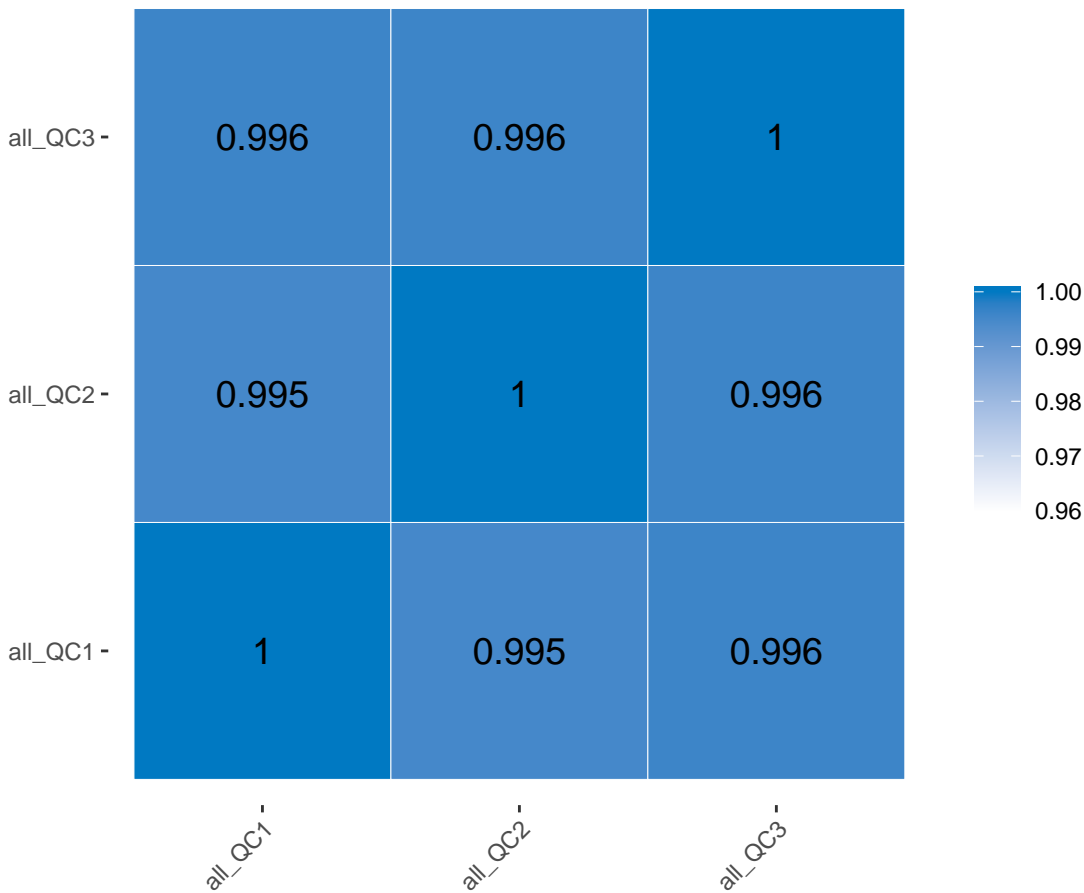

Supplement: Supplementary file 1 [file ijms-27-05895-s001.zip › result/1.MetQuant-QC/Correlation/cor_pearson_all.pdf]

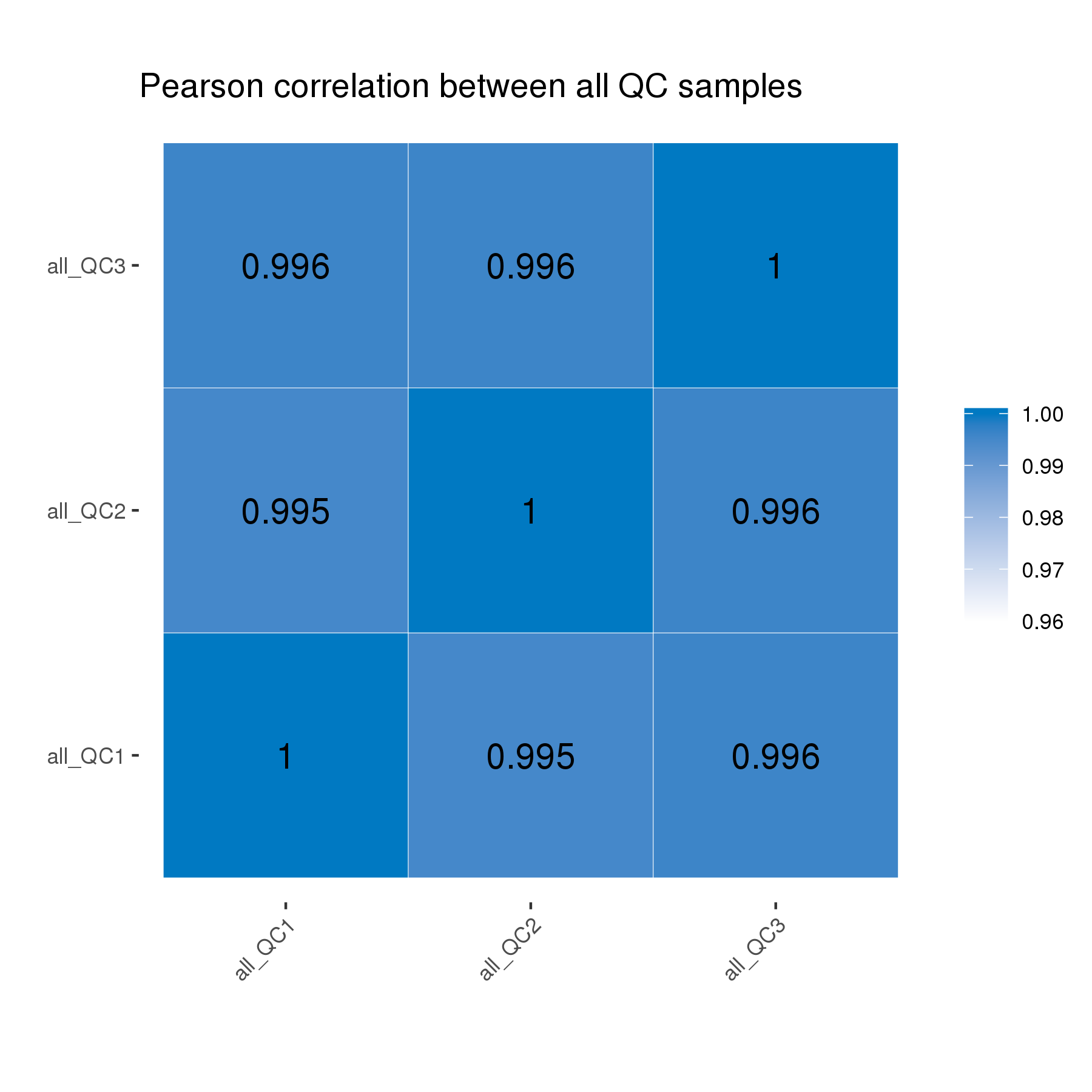

Supplement: Supplementary file 1 [file ijms-27-05895-s001.zip › result/1.MetQuant-QC/Correlation/cor_pearson_all.png]

# ClassI

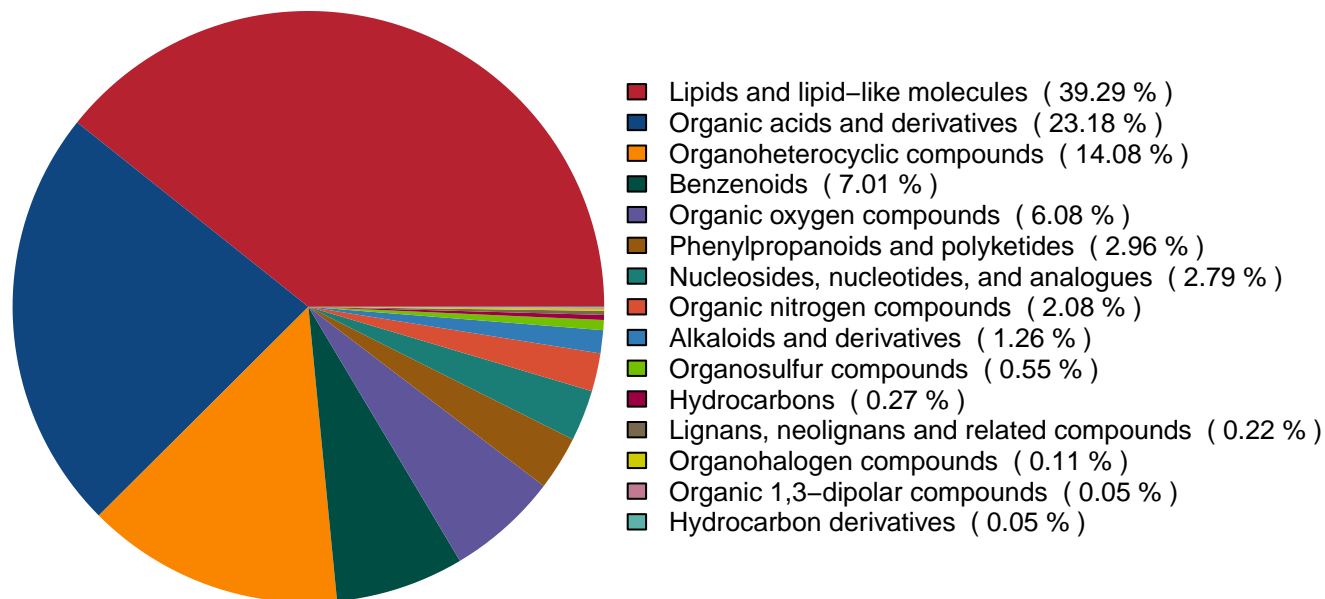

Supplement: Supplementary file 1 [file ijms-27-05895-s001.zip › result/1.MetQuant-QC/Pie_Chart_all.pdf]

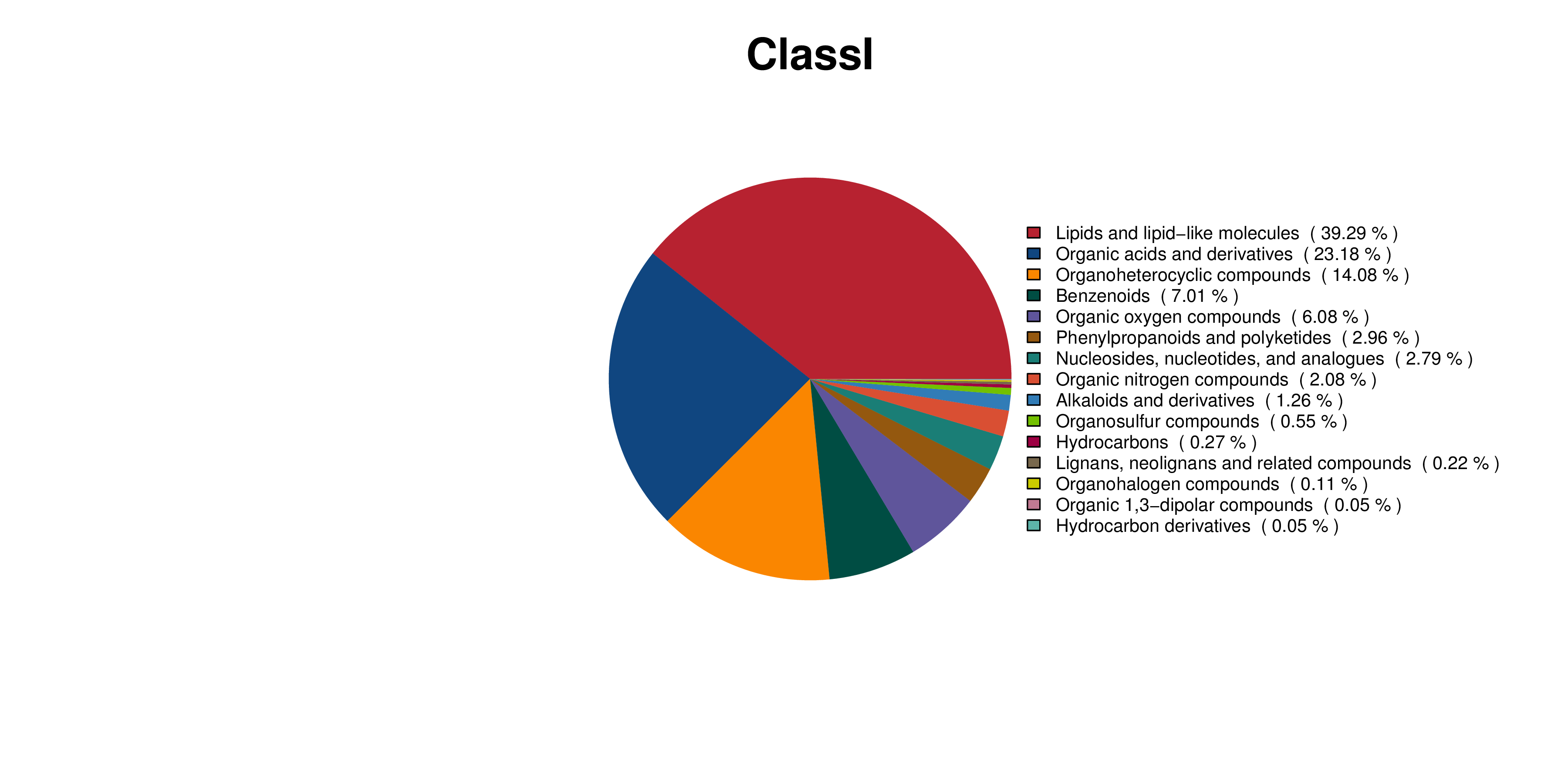

Supplement: Supplementary file 1 [file ijms-27-05895-s001.zip › result/1.MetQuant-QC/Pie_Chart_all.png]

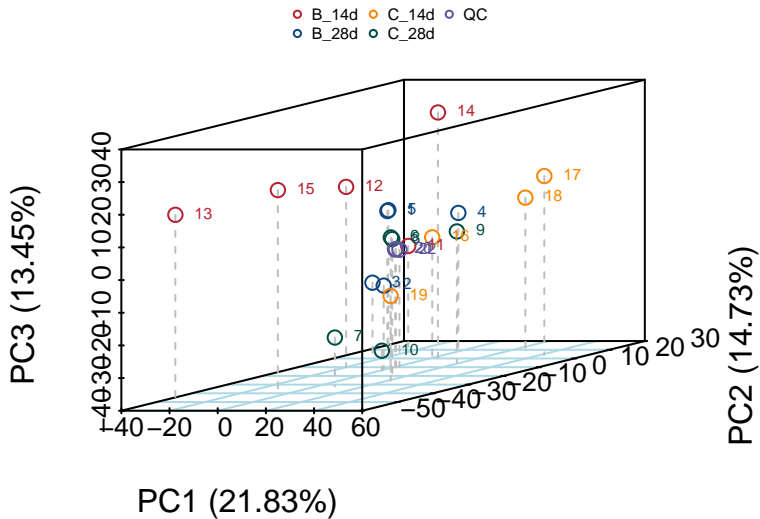

Supplement: Supplementary file 1 [file ijms-27-05895-s001.zip › result/1.MetQuant-QC/Samples_QC_all-PCA.3D.pdf]

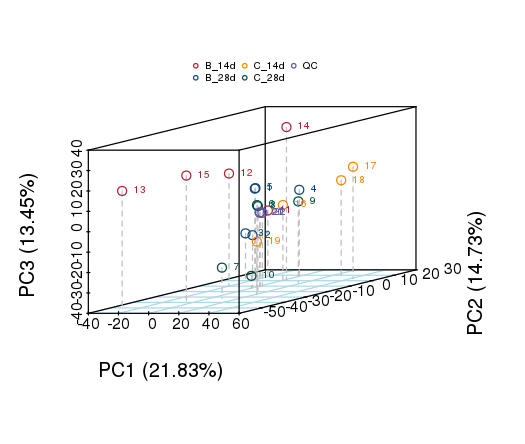

Supplement: Supplementary file 1 [file ijms-27-05895-s001.zip › result/1.MetQuant-QC/Samples_QC_all-PCA.3D.png]

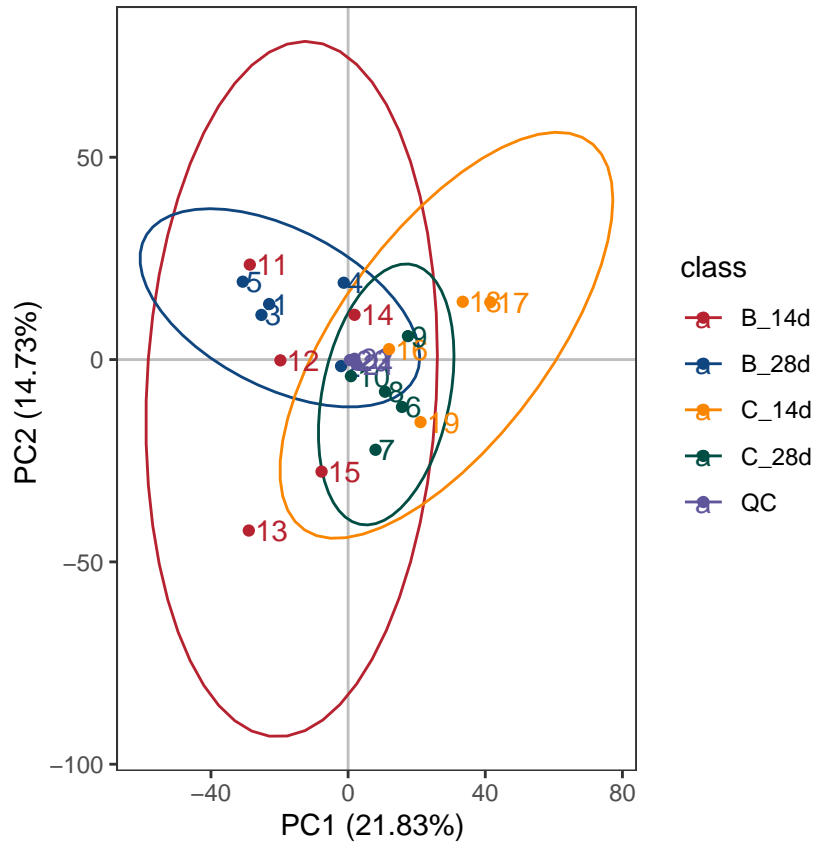

Supplement: Supplementary file 1 [file ijms-27-05895-s001.zip › result/1.MetQuant-QC/Samples_QC_all-PCA.pdf]

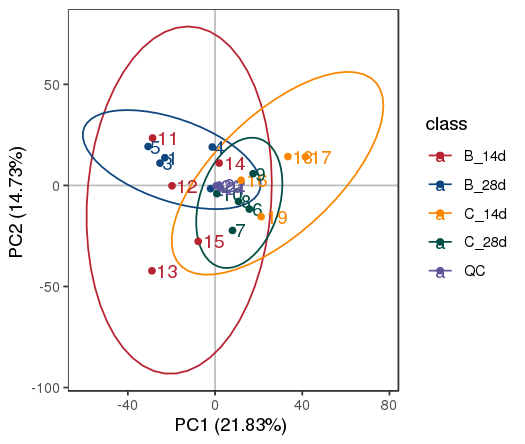

Supplement: Supplementary file 1 [file ijms-27-05895-s001.zip › result/1.MetQuant-QC/Samples_QC_all-PCA.png]

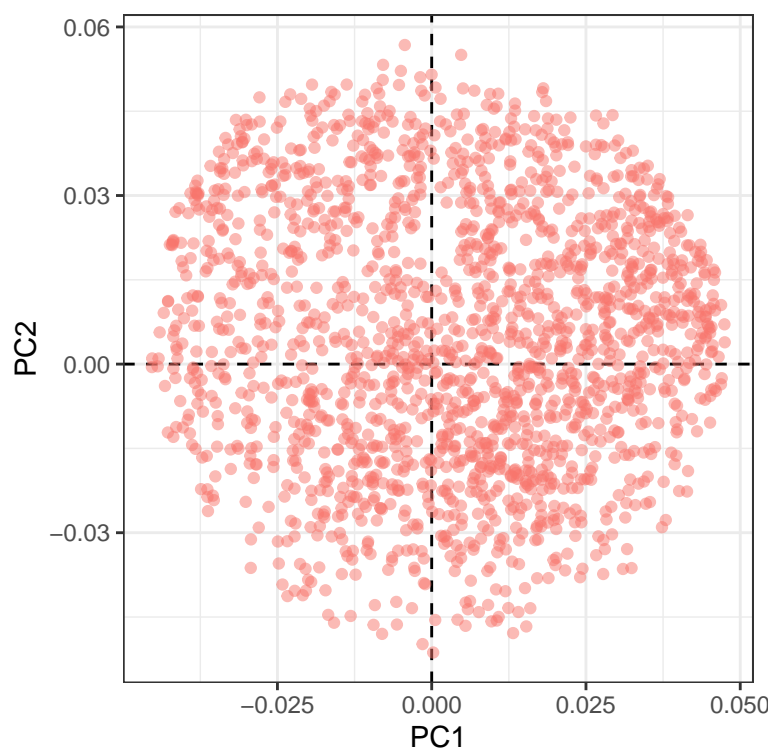

Supplement: Supplementary file 1 [file ijms-27-05895-s001.zip › result/1.MetQuant-QC/Samples_QC_all-pcaloading.pdf]

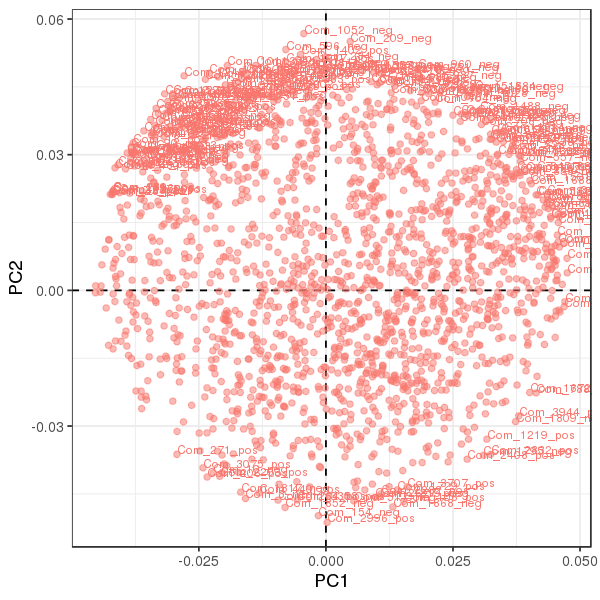

Supplement: Supplementary file 1 [file ijms-27-05895-s001.zip › result/1.MetQuant-QC/Samples_QC_all-pcaloading.png]

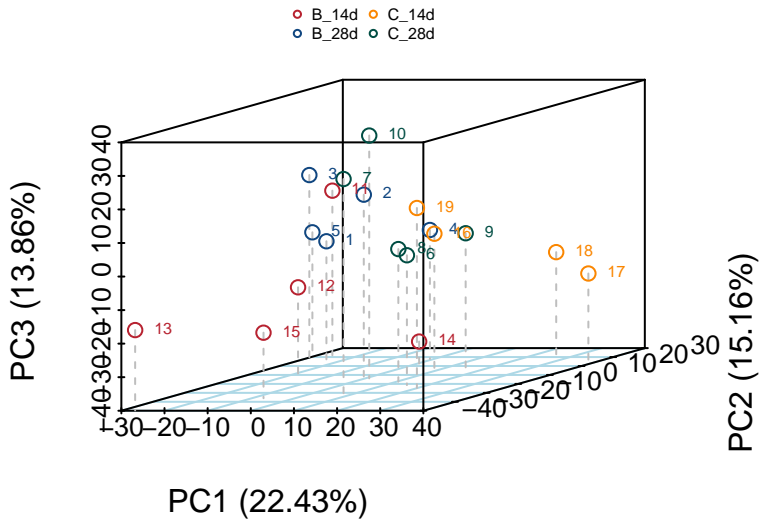

Supplement: Supplementary file 1 [file ijms-27-05895-s001.zip › result/1.MetQuant-QC/Samples_all-PCA.3D.pdf]

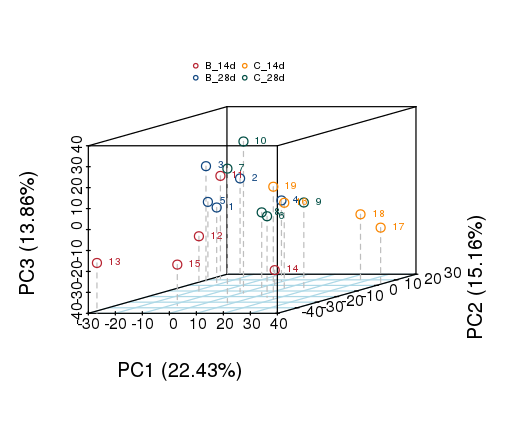

Supplement: Supplementary file 1 [file ijms-27-05895-s001.zip › result/1.MetQuant-QC/Samples_all-PCA.3D.png]

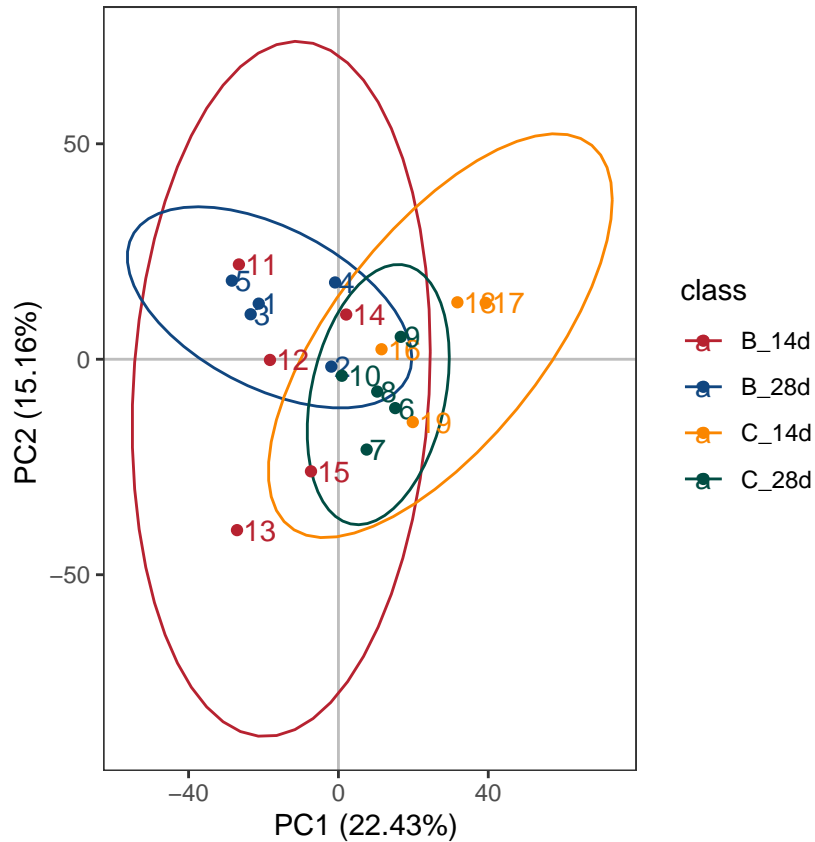

Supplement: Supplementary file 1 [file ijms-27-05895-s001.zip › result/1.MetQuant-QC/Samples_all-PCA.pdf]

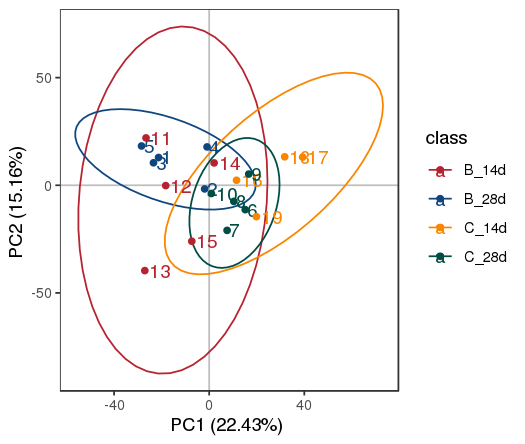

Supplement: Supplementary file 1 [file ijms-27-05895-s001.zip › result/1.MetQuant-QC/Samples_all-PCA.png]

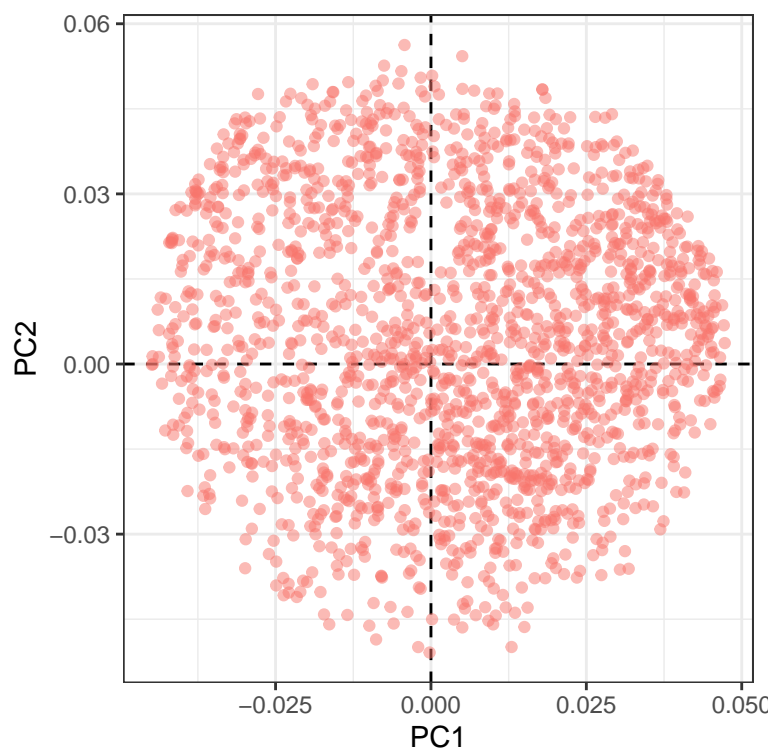

Supplement: Supplementary file 1 [file ijms-27-05895-s001.zip › result/1.MetQuant-QC/Samples_all-pcaloading.pdf]

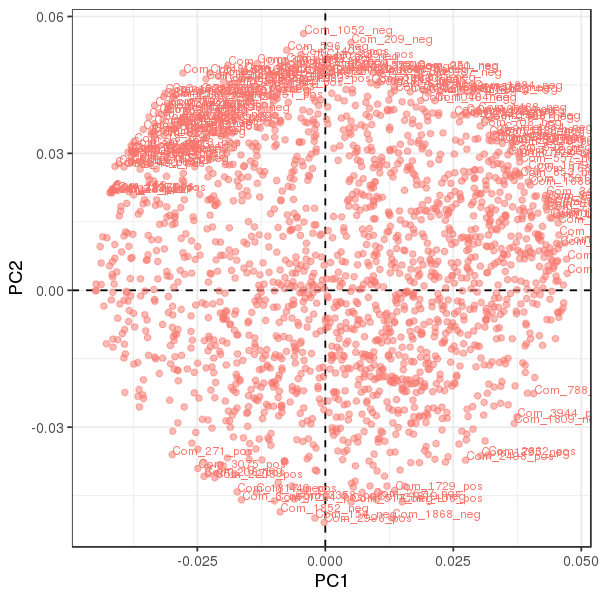

Supplement: Supplementary file 1 [file ijms-27-05895-s001.zip › result/1.MetQuant-QC/Samples_all-pcaloading.png]

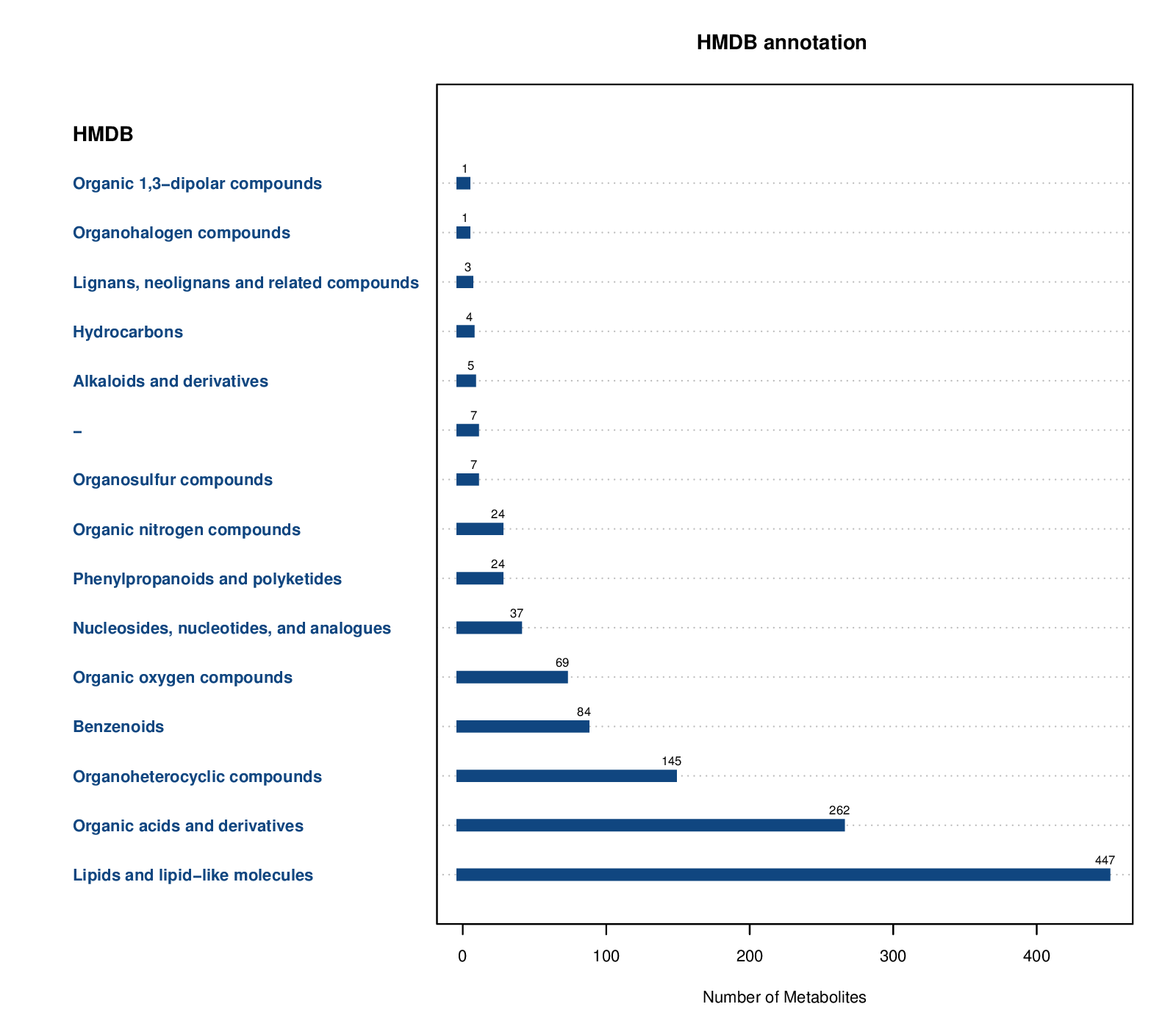

Supplement: Supplementary file 1 [file ijms-27-05895-s001.zip › result/2.MetAnnotation/HMDB/meta_all.HMDB.Anno.png]

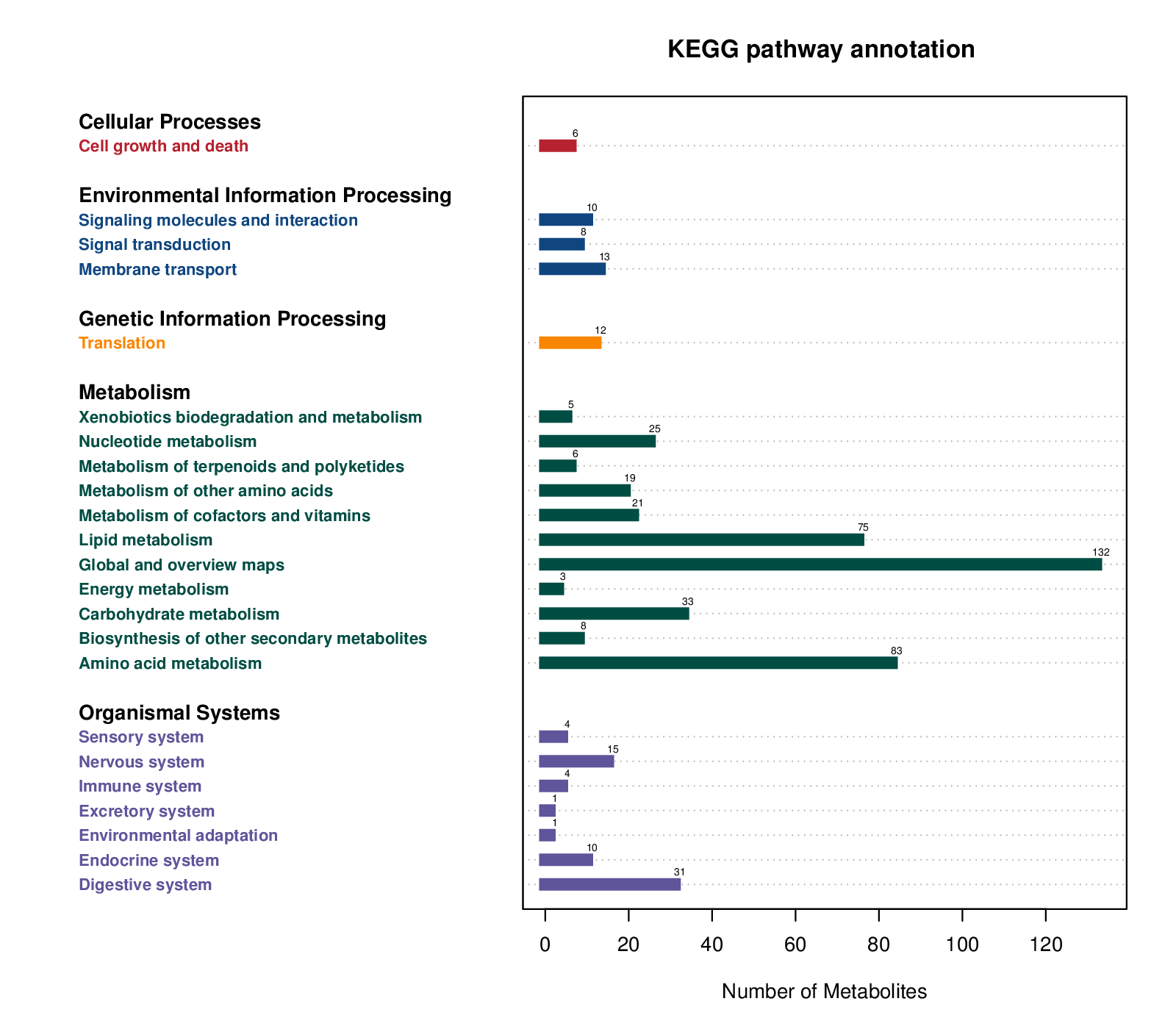

Supplement: Supplementary file 1 [file ijms-27-05895-s001.zip › result/2.MetAnnotation/KEGG/meta_all.KEGG.Anno.png]

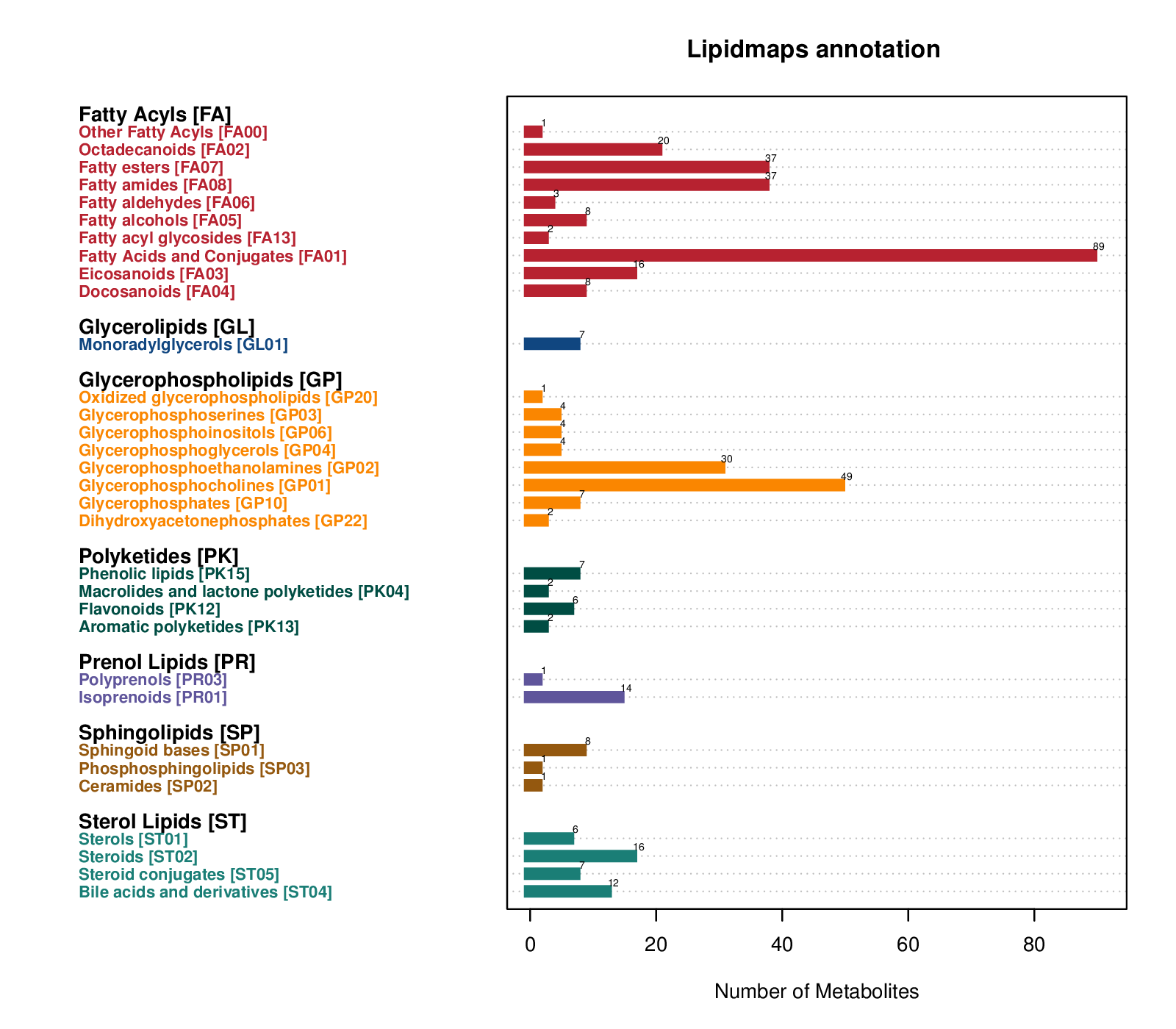

Supplement: Supplementary file 1 [file ijms-27-05895-s001.zip › result/2.MetAnnotation/Lipidmaps/meta_all.Lipidmaps.Anno.png]

B\_14d.vs.C\_14d

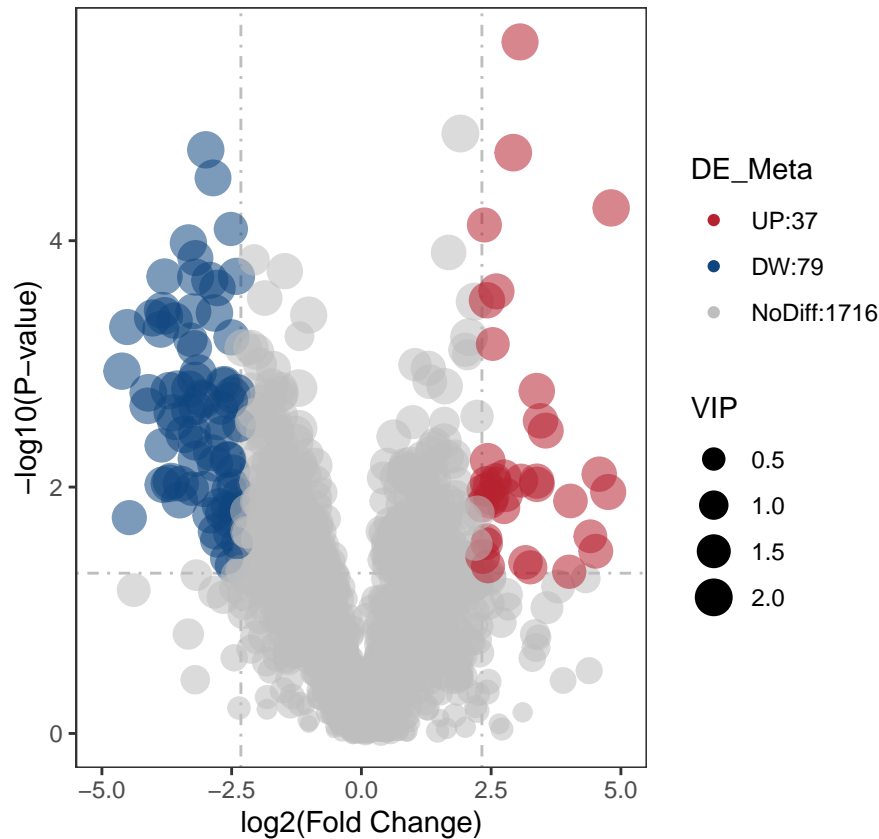

Supplement: Supplementary file 1 [file ijms-27-05895-s001.zip › result/3.MetDiffScreening/B_14d.vs.C_14d/B_14d.vs.C_14d_all.xls.volcano.pdf]

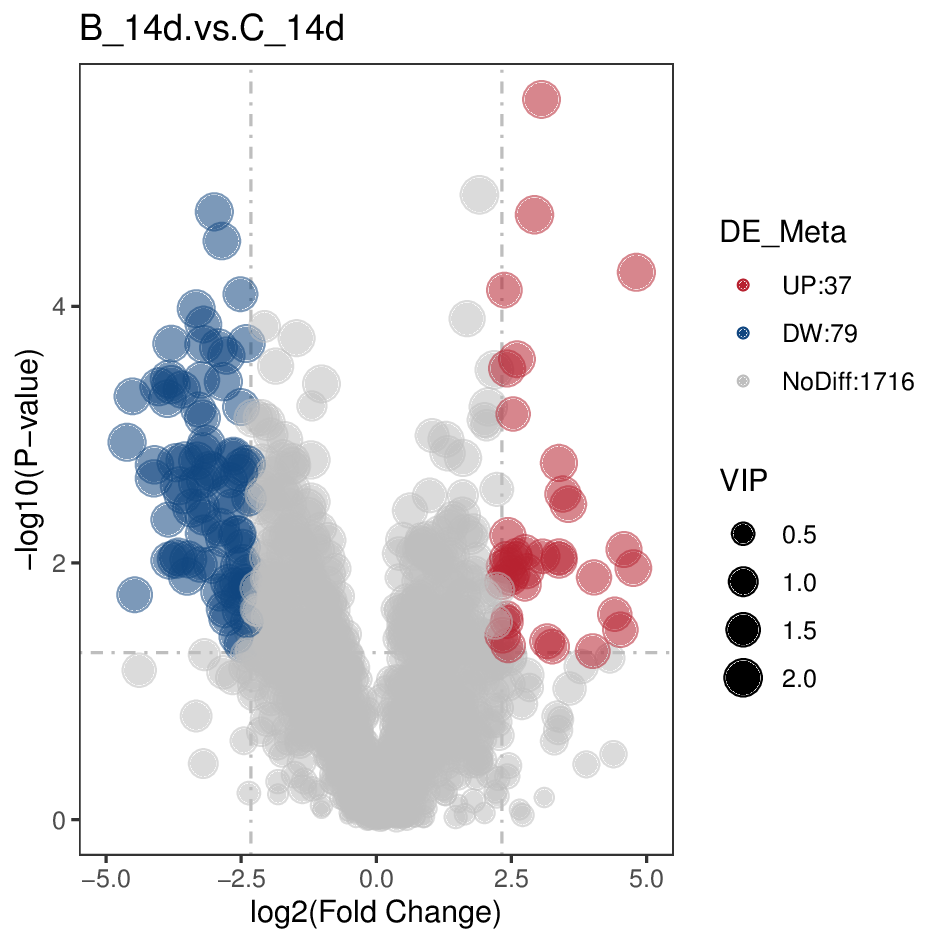

Supplement: Supplementary file 1 [file ijms-27-05895-s001.zip › result/3.MetDiffScreening/B_14d.vs.C_14d/B_14d.vs.C_14d_all.xls.volcano.png]

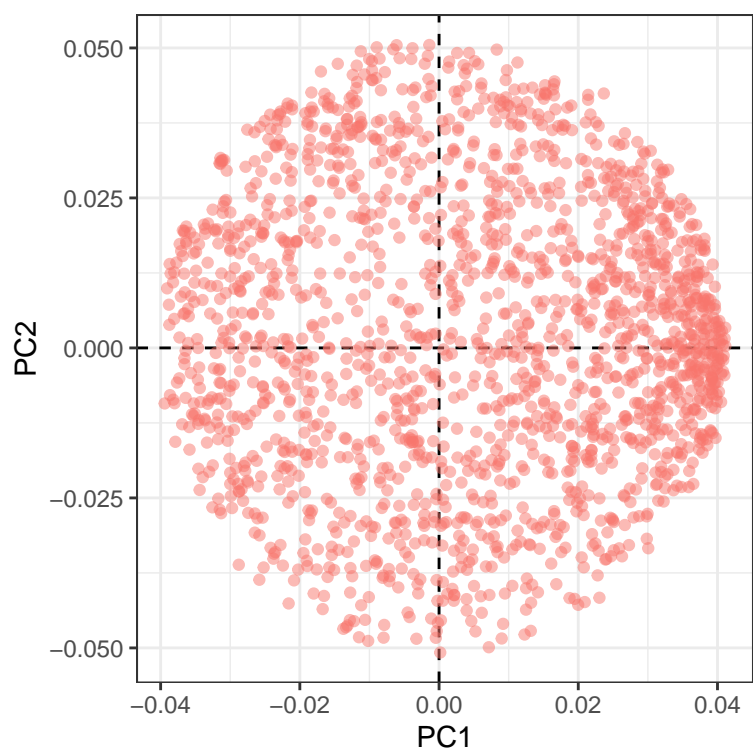

Supplement: Supplementary file 1 [file ijms-27-05895-s001.zip › result/3.MetDiffScreening/B_14d.vs.C_14d/B_14d.vs.C_14d_all_PCA-pcaloading.pdf]

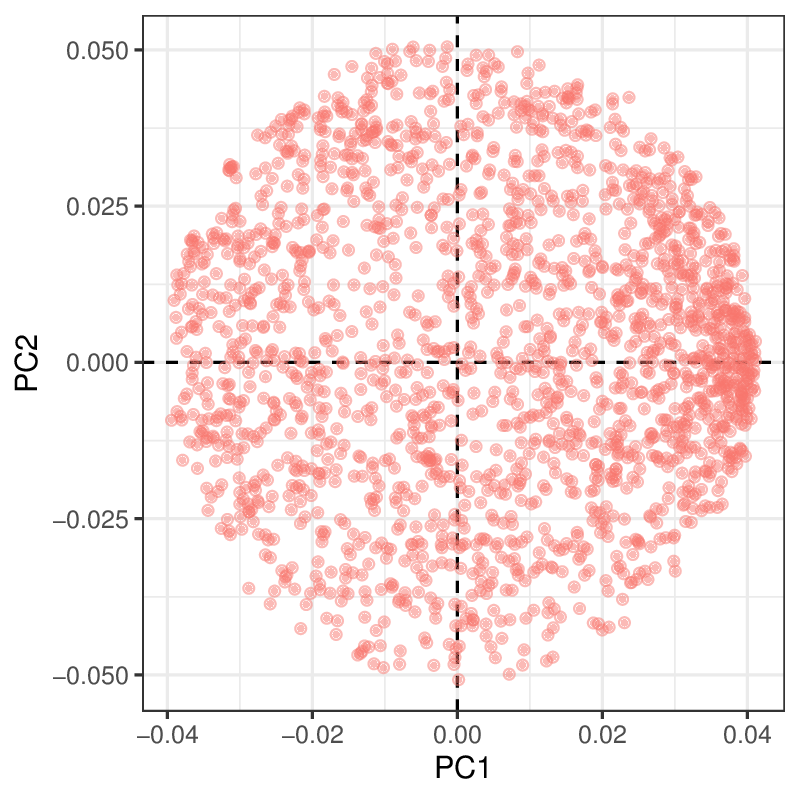

Supplement: Supplementary file 1 [file ijms-27-05895-s001.zip › result/3.MetDiffScreening/B_14d.vs.C_14d/B_14d.vs.C_14d_all_PCA-pcaloading.png]

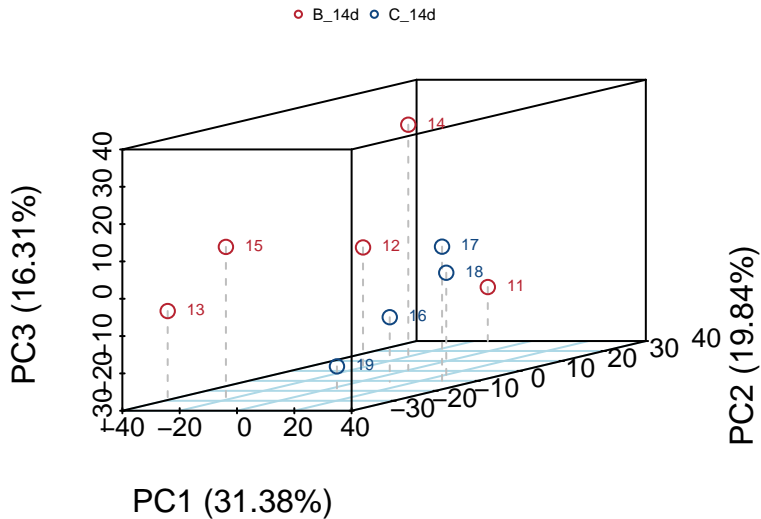

Supplement: Supplementary file 1 [file ijms-27-05895-s001.zip › result/3.MetDiffScreening/B_14d.vs.C_14d/B_14d.vs.C_14d_all_PCA.3D.pdf]

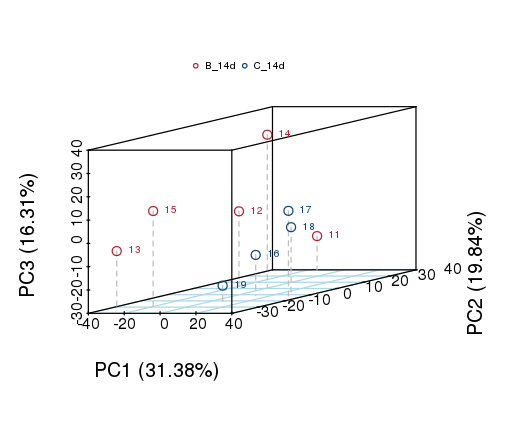

Supplement: Supplementary file 1 [file ijms-27-05895-s001.zip › result/3.MetDiffScreening/B_14d.vs.C_14d/B_14d.vs.C_14d_all_PCA.3D.png]

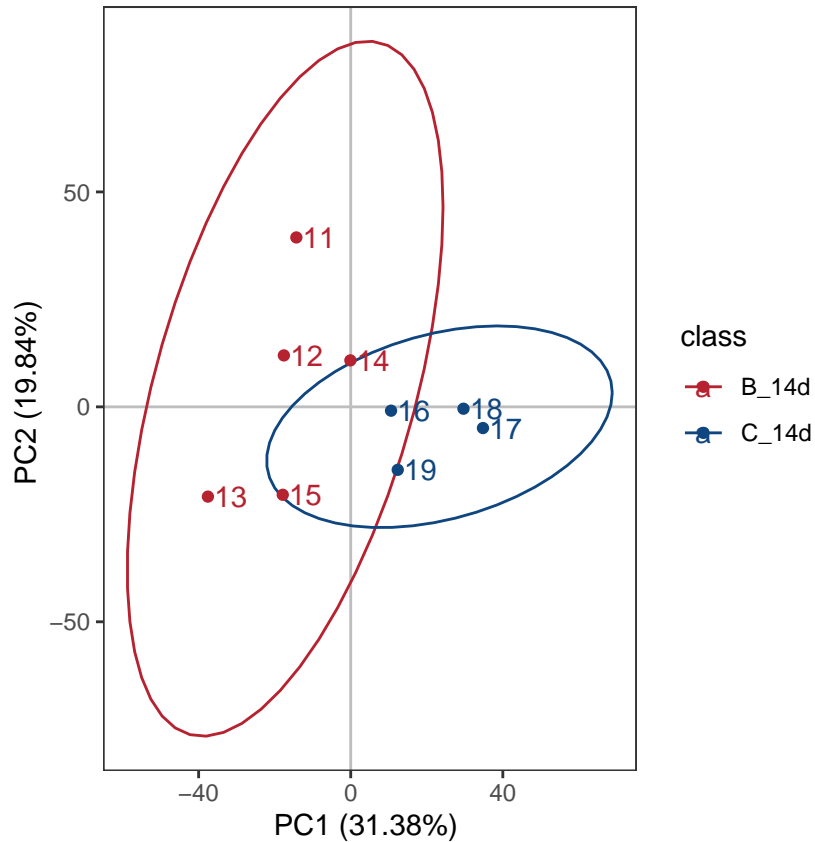

Supplement: Supplementary file 1 [file ijms-27-05895-s001.zip › result/3.MetDiffScreening/B_14d.vs.C_14d/B_14d.vs.C_14d_all_PCA.pdf]

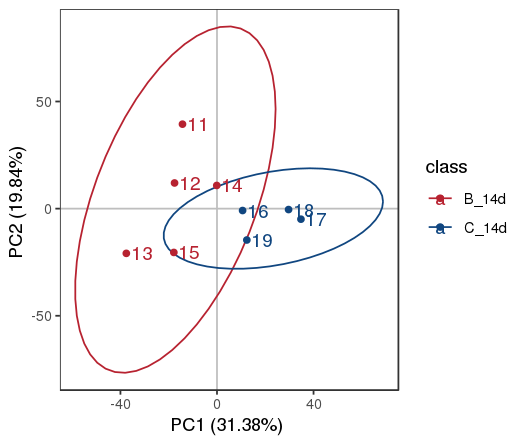

Supplement: Supplementary file 1 [file ijms-27-05895-s001.zip › result/3.MetDiffScreening/B_14d.vs.C_14d/B_14d.vs.C_14d_all_PCA.png]

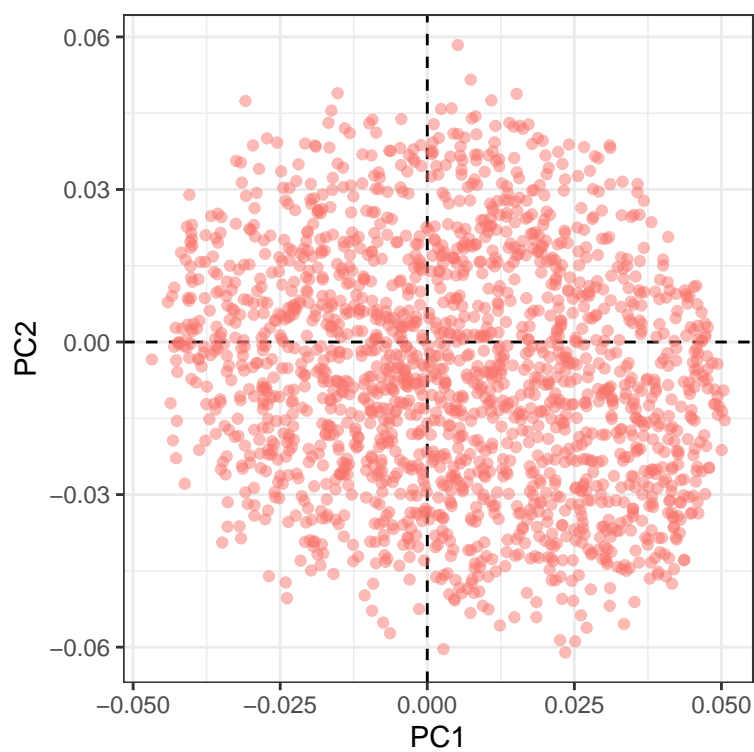

Supplement: Supplementary file 1 [file ijms-27-05895-s001.zip › result/3.MetDiffScreening/B_14d.vs.C_14d/B_14d.vs.C_14d_all_PLSDA-loading.pdf]

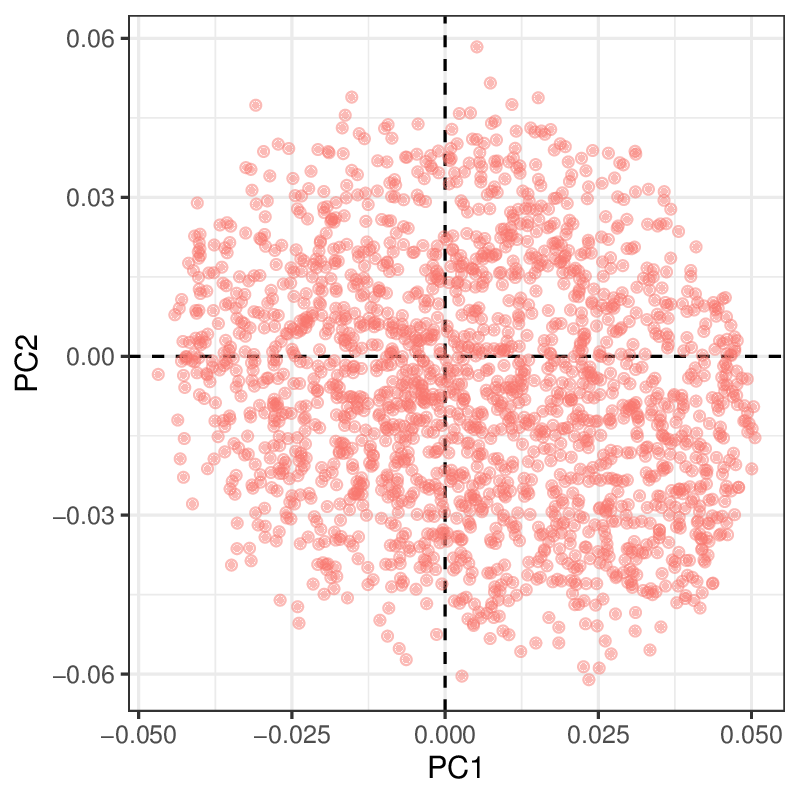

Supplement: Supplementary file 1 [file ijms-27-05895-s001.zip › result/3.MetDiffScreening/B_14d.vs.C_14d/B_14d.vs.C_14d_all_PLSDA-loading.png]

class B\_14d C\_14d

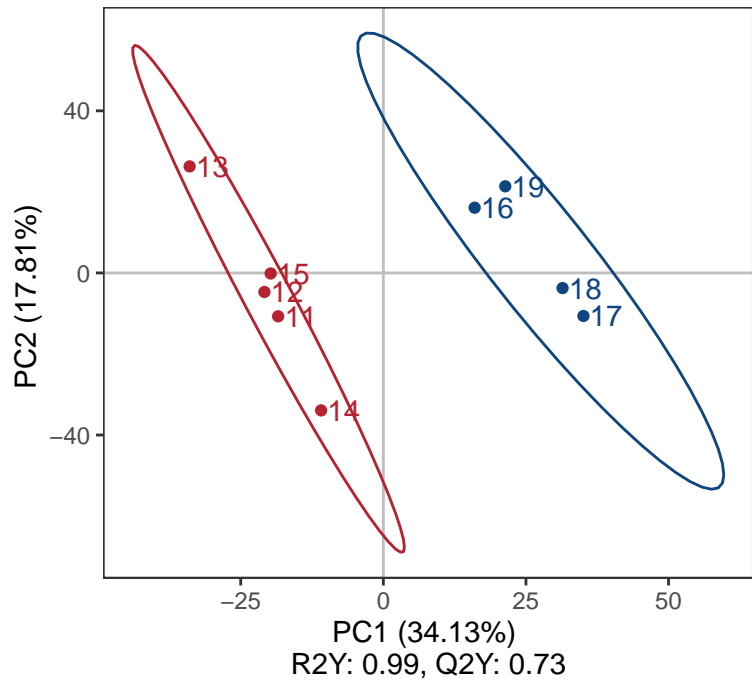

Supplement: Supplementary file 1 [file ijms-27-05895-s001.zip › result/3.MetDiffScreening/B_14d.vs.C_14d/B_14d.vs.C_14d_all_PLSDA-score.pdf]

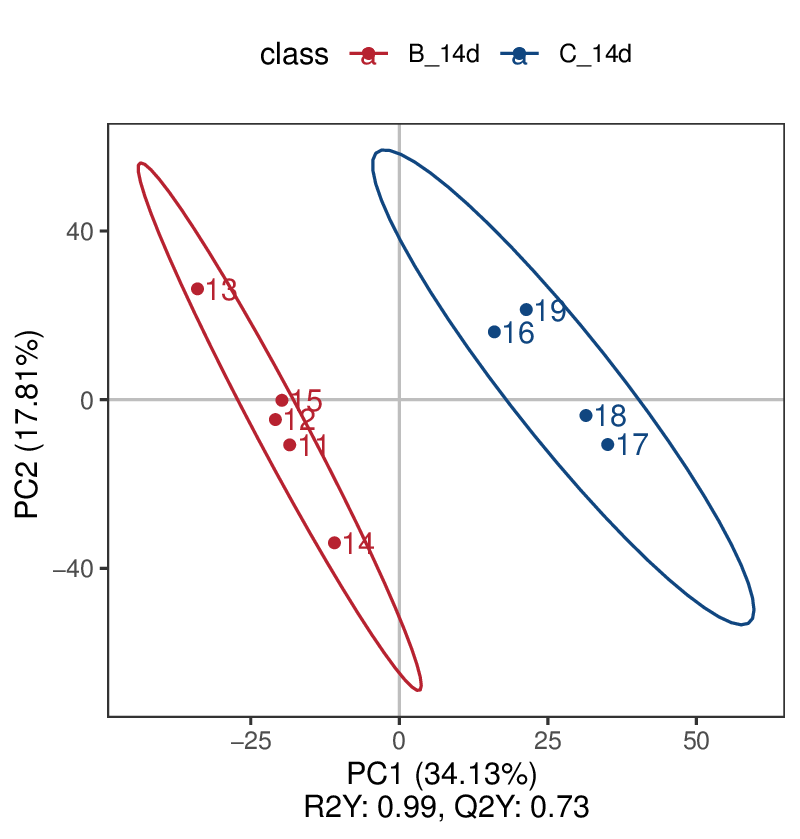

Supplement: Supplementary file 1 [file ijms-27-05895-s001.zip › result/3.MetDiffScreening/B_14d.vs.C_14d/B_14d.vs.C_14d_all_PLSDA-score.png]

B\_14d\_C\_14d

Intercepts: R2=(0.0,0.87), Q2=(0.0,-1.11)

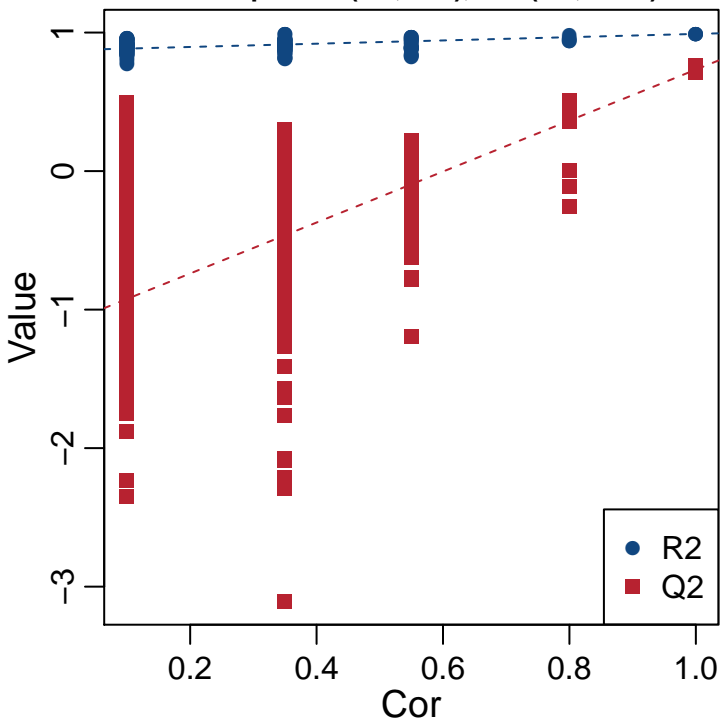

Supplement: Supplementary file 1 [file ijms-27-05895-s001.zip › result/3.MetDiffScreening/B_14d.vs.C_14d/B_14d.vs.C_14d_all_PLSDA-valid.pdf]

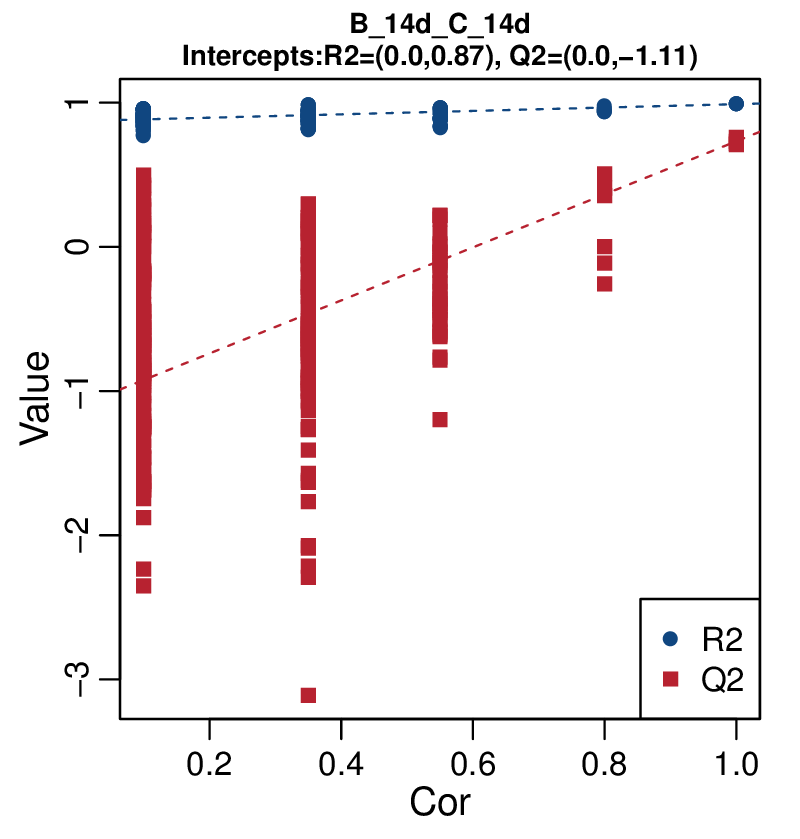

Supplement: Supplementary file 1 [file ijms-27-05895-s001.zip › result/3.MetDiffScreening/B_14d.vs.C_14d/B_14d.vs.C_14d_all_PLSDA-valid.png]

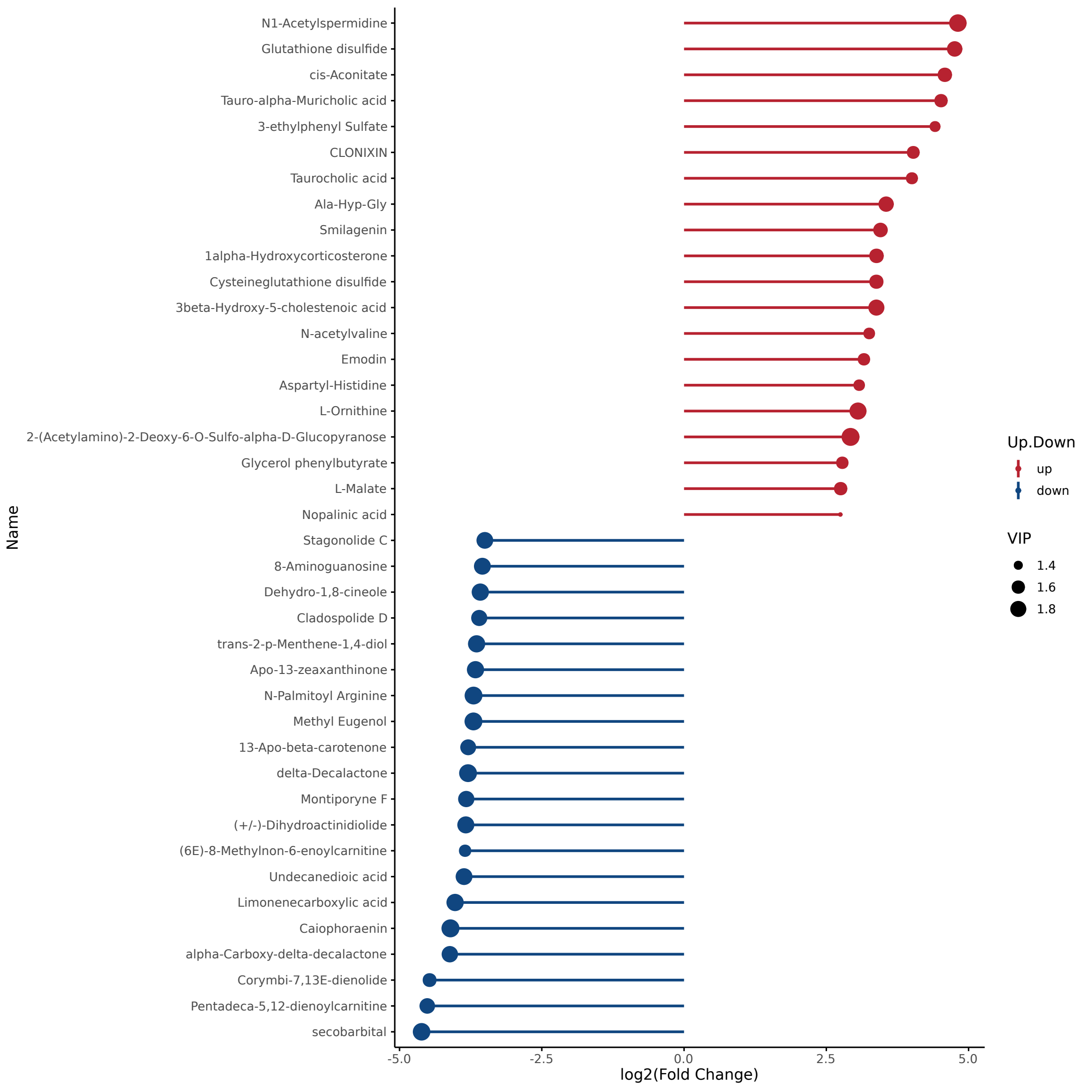

Supplement: Supplementary file 1 [file ijms-27-05895-s001.zip › result/3.MetDiffScreening/B_14d.vs.C_14d/B_14d.vs.C_14d_all_stem.pdf]

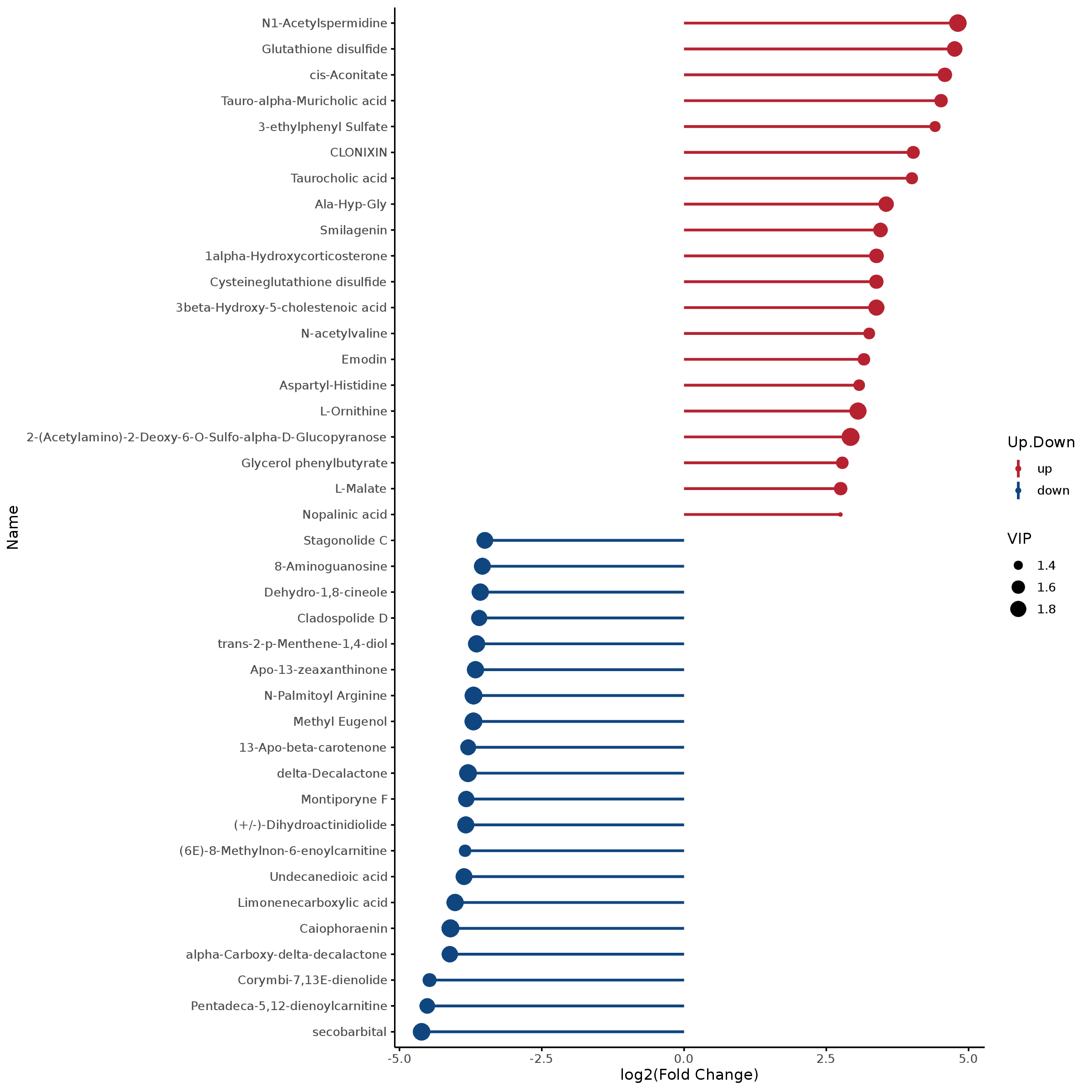

Supplement: Supplementary file 1 [file ijms-27-05895-s001.zip › result/3.MetDiffScreening/B_14d.vs.C_14d/B_14d.vs.C_14d_all_stem.png]

B\_28d.vs.C\_28d

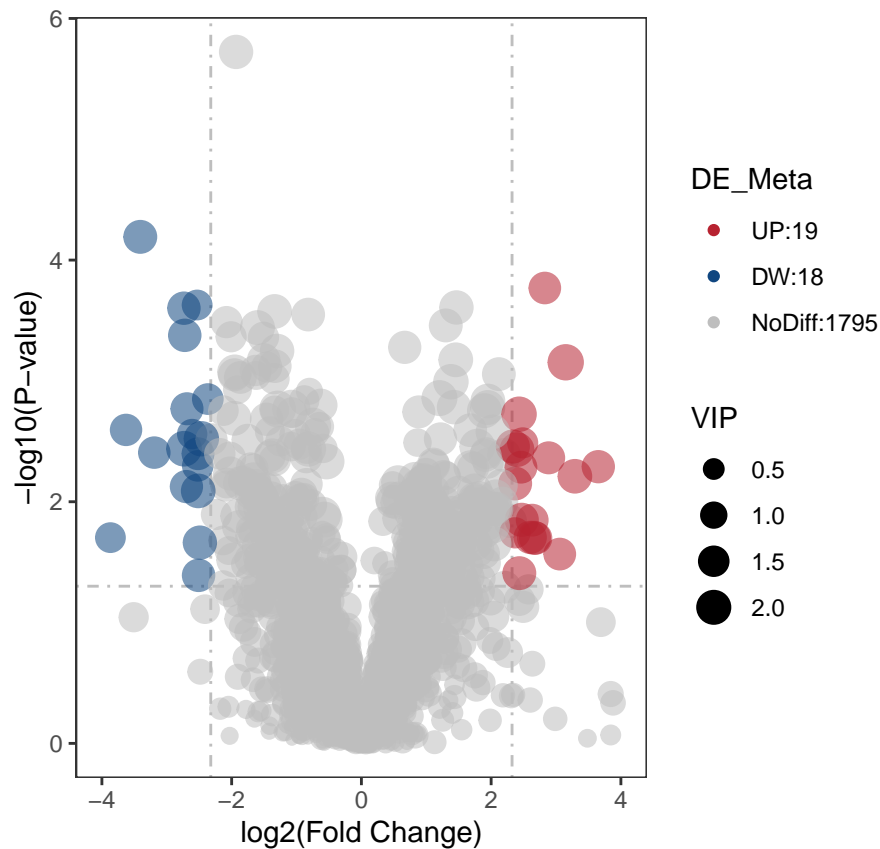

Supplement: Supplementary file 1 [file ijms-27-05895-s001.zip › result/3.MetDiffScreening/B_28d.vs.C_28d/B_28d.vs.C_28d_all.xls.volcano.pdf]

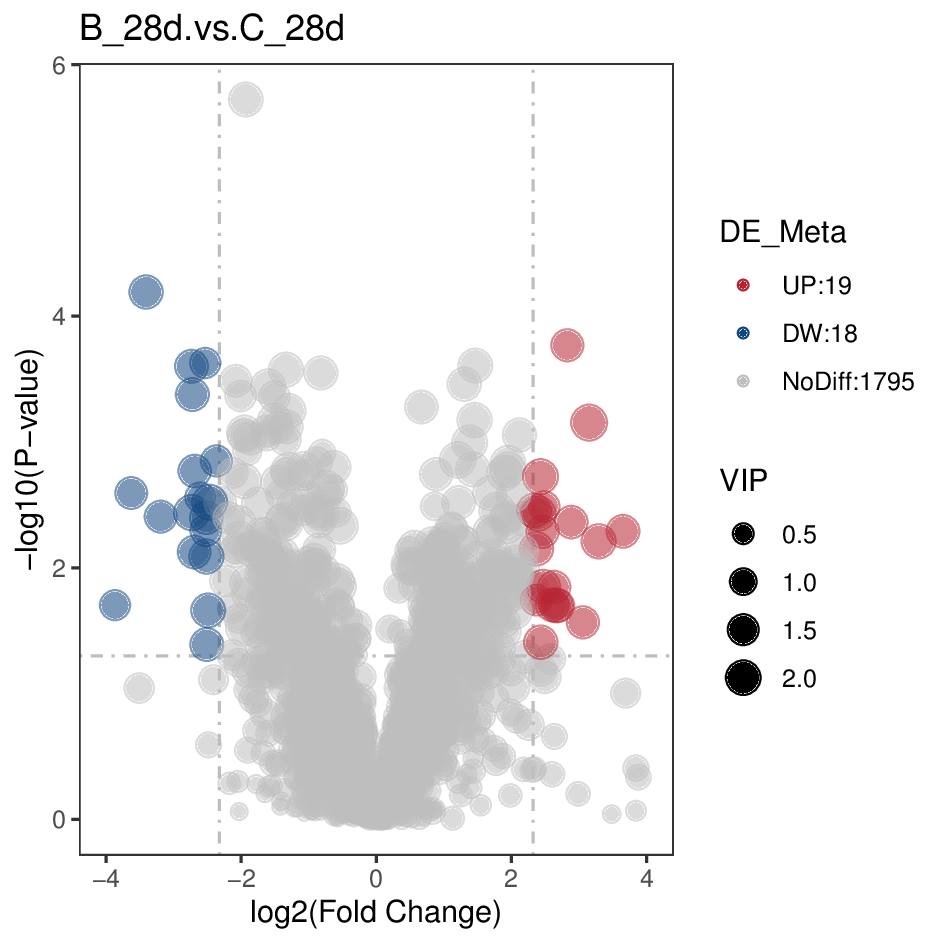

Supplement: Supplementary file 1 [file ijms-27-05895-s001.zip › result/3.MetDiffScreening/B_28d.vs.C_28d/B_28d.vs.C_28d_all.xls.volcano.png]

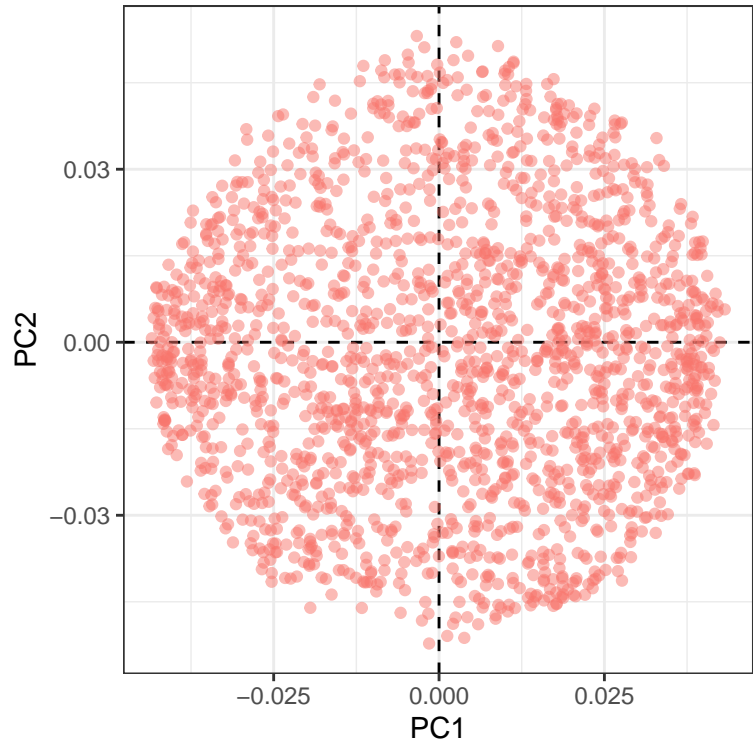

Supplement: Supplementary file 1 [file ijms-27-05895-s001.zip › result/3.MetDiffScreening/B_28d.vs.C_28d/B_28d.vs.C_28d_all_PCA-pcaloading.pdf]

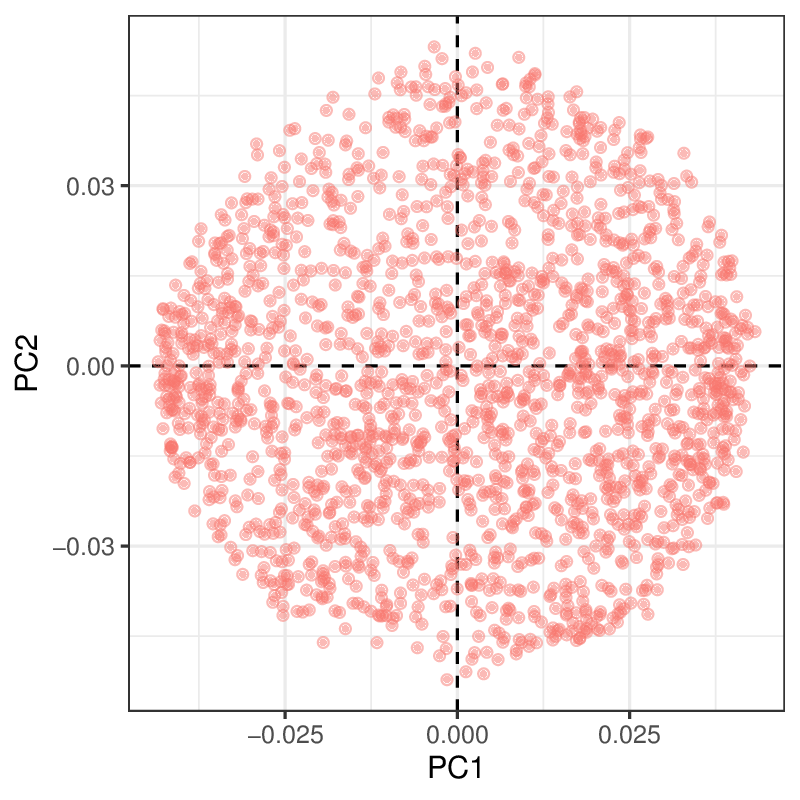

Supplement: Supplementary file 1 [file ijms-27-05895-s001.zip › result/3.MetDiffScreening/B_28d.vs.C_28d/B_28d.vs.C_28d_all_PCA-pcaloading.png]

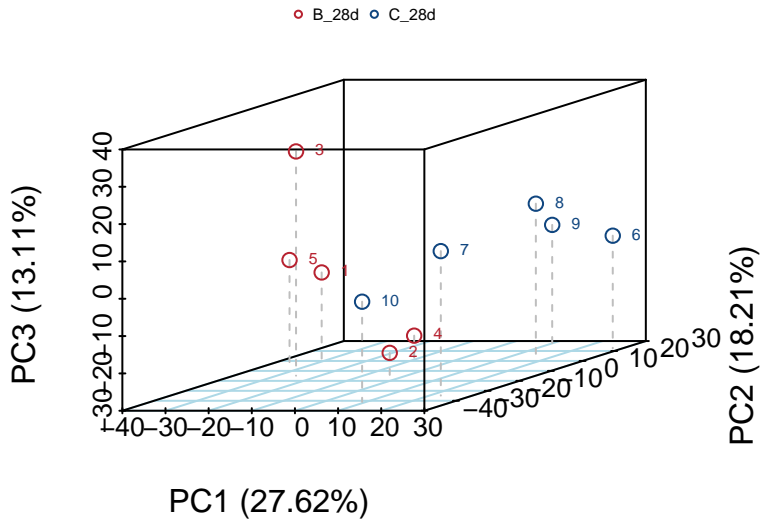

Supplement: Supplementary file 1 [file ijms-27-05895-s001.zip › result/3.MetDiffScreening/B_28d.vs.C_28d/B_28d.vs.C_28d_all_PCA.3D.pdf]

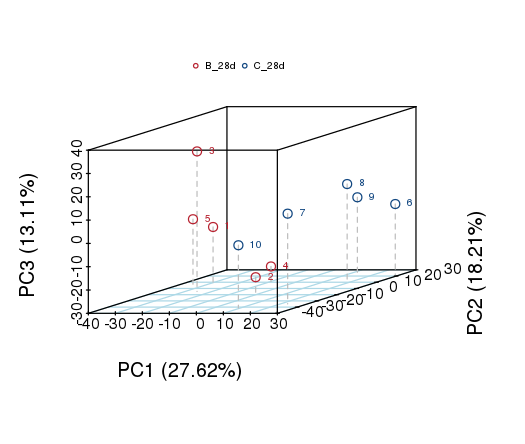

Supplement: Supplementary file 1 [file ijms-27-05895-s001.zip › result/3.MetDiffScreening/B_28d.vs.C_28d/B_28d.vs.C_28d_all_PCA.3D.png]

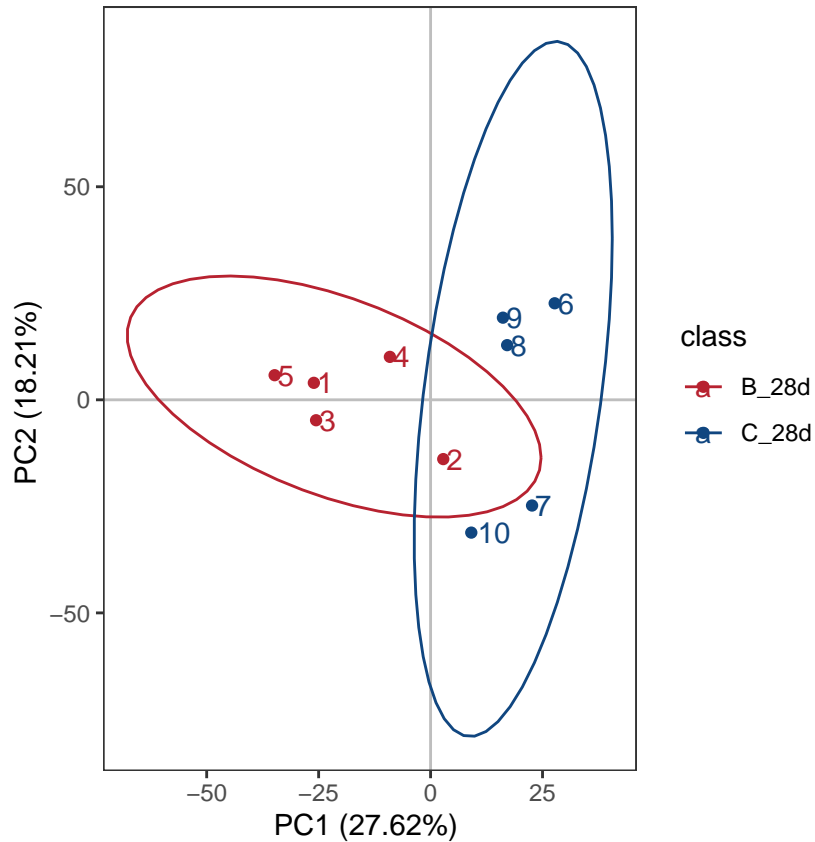

Supplement: Supplementary file 1 [file ijms-27-05895-s001.zip › result/3.MetDiffScreening/B_28d.vs.C_28d/B_28d.vs.C_28d_all_PCA.pdf]

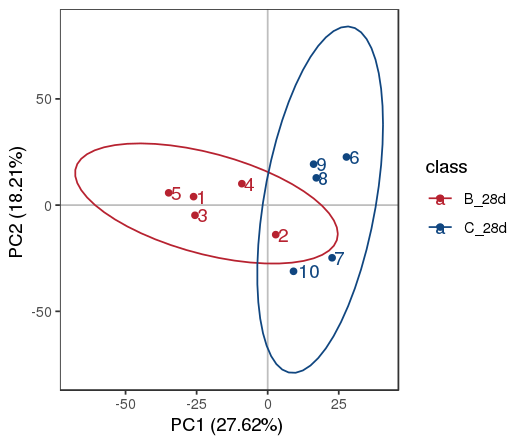

Supplement: Supplementary file 1 [file ijms-27-05895-s001.zip › result/3.MetDiffScreening/B_28d.vs.C_28d/B_28d.vs.C_28d_all_PCA.png]

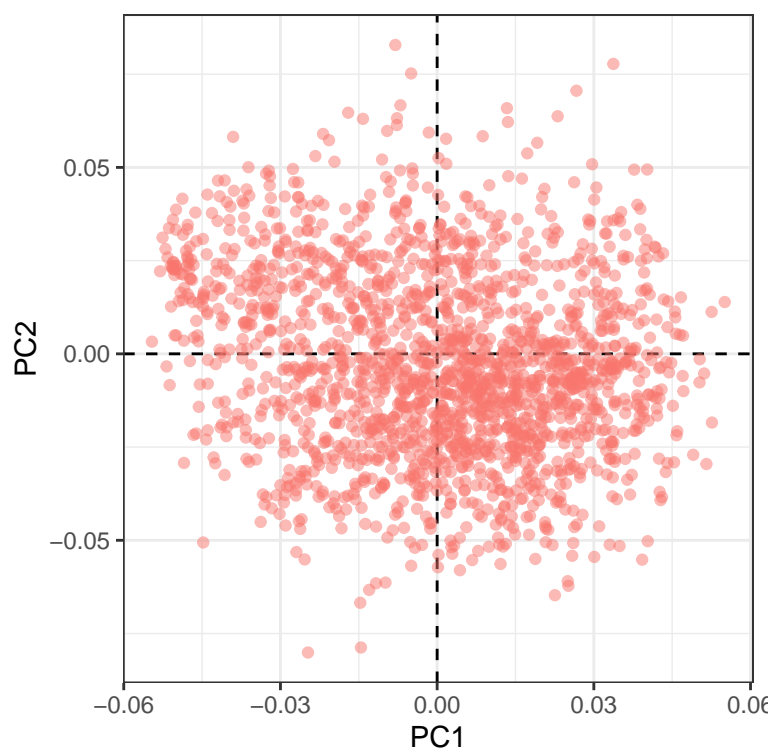

Supplement: Supplementary file 1 [file ijms-27-05895-s001.zip › result/3.MetDiffScreening/B_28d.vs.C_28d/B_28d.vs.C_28d_all_PLSDA-loading.pdf]

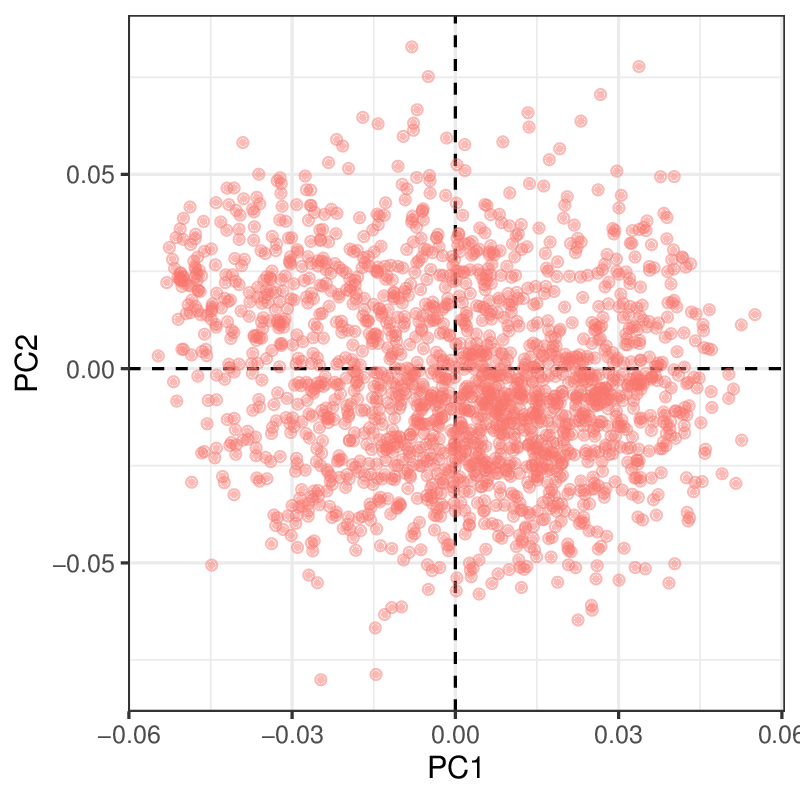

Supplement: Supplementary file 1 [file ijms-27-05895-s001.zip › result/3.MetDiffScreening/B_28d.vs.C_28d/B_28d.vs.C_28d_all_PLSDA-loading.png]

class B\_28d C\_28d

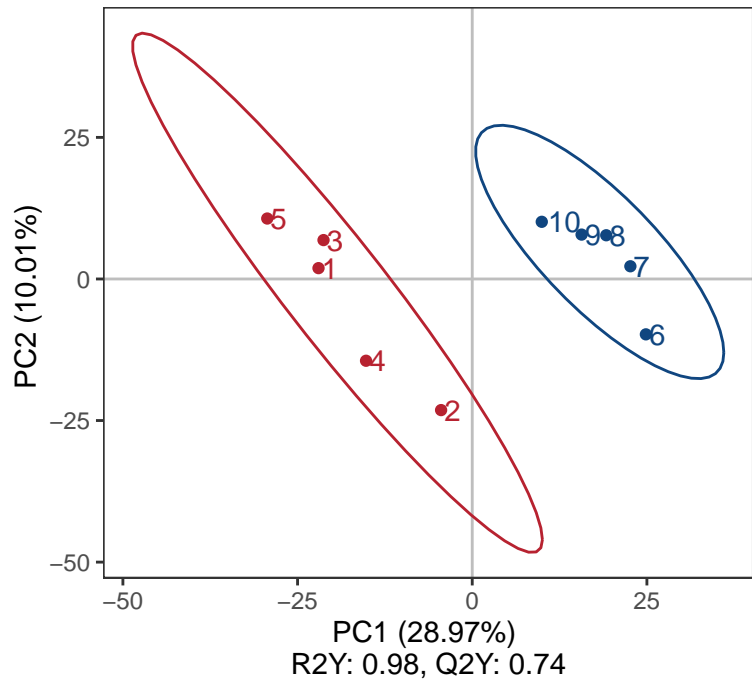

Supplement: Supplementary file 1 [file ijms-27-05895-s001.zip › result/3.MetDiffScreening/B_28d.vs.C_28d/B_28d.vs.C_28d_all_PLSDA-score.pdf]

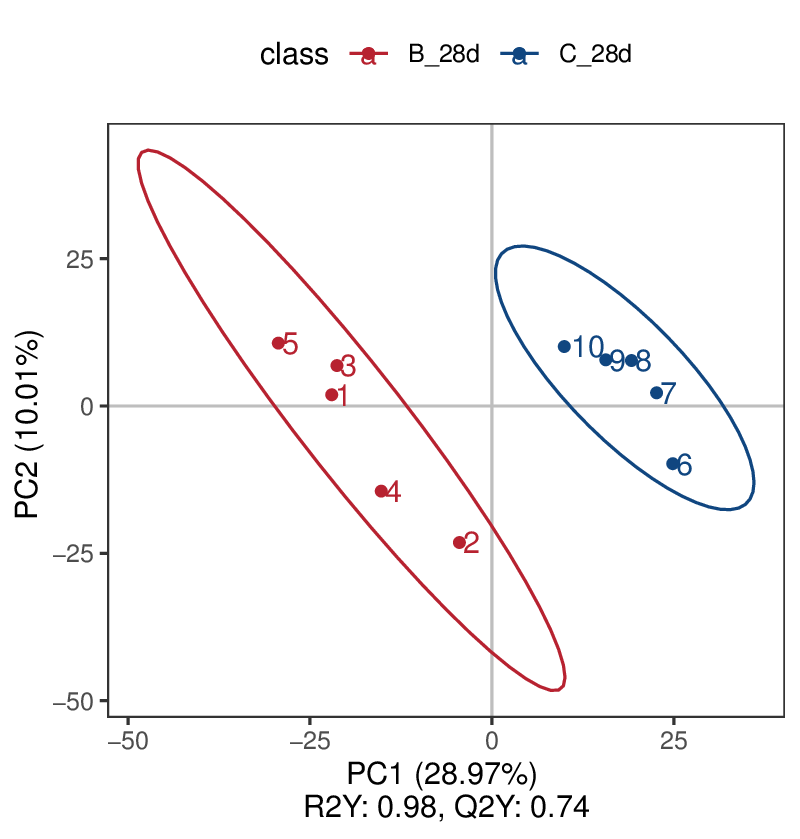

Supplement: Supplementary file 1 [file ijms-27-05895-s001.zip › result/3.MetDiffScreening/B_28d.vs.C_28d/B_28d.vs.C_28d_all_PLSDA-score.png]

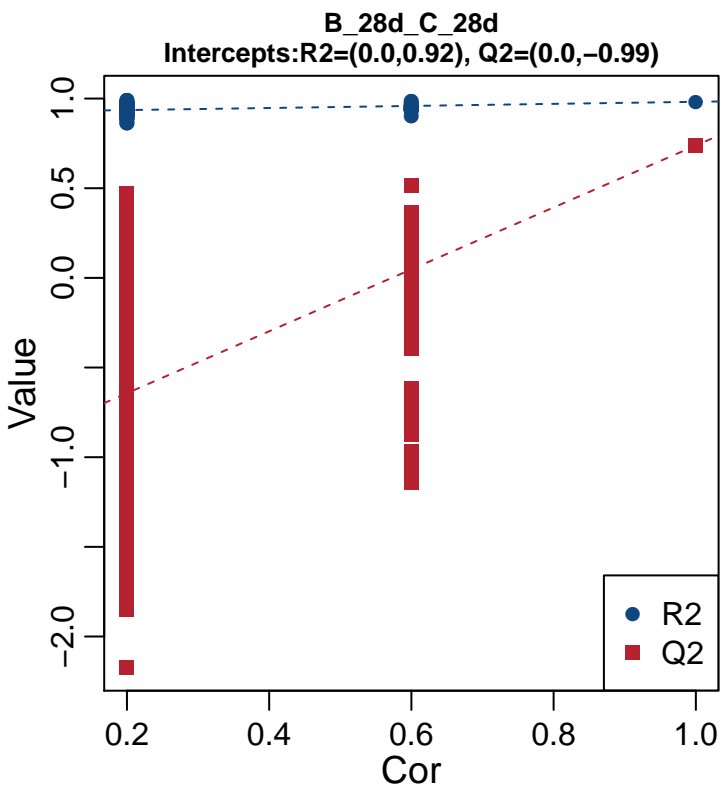

Supplement: Supplementary file 1 [file ijms-27-05895-s001.zip › result/3.MetDiffScreening/B_28d.vs.C_28d/B_28d.vs.C_28d_all_PLSDA-valid.pdf]

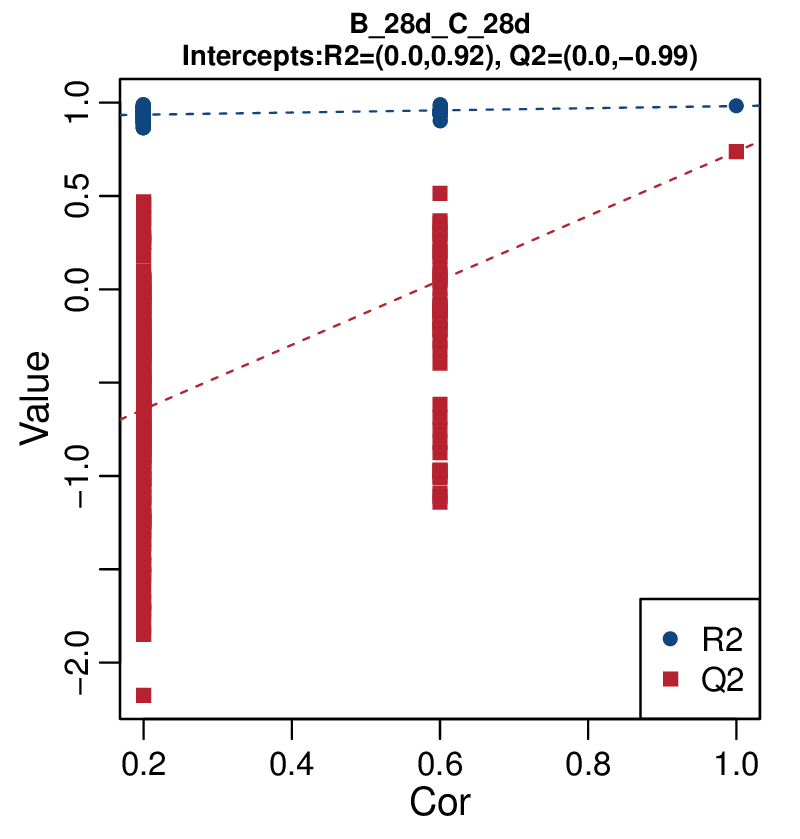

Supplement: Supplementary file 1 [file ijms-27-05895-s001.zip › result/3.MetDiffScreening/B_28d.vs.C_28d/B_28d.vs.C_28d_all_PLSDA-valid.png]

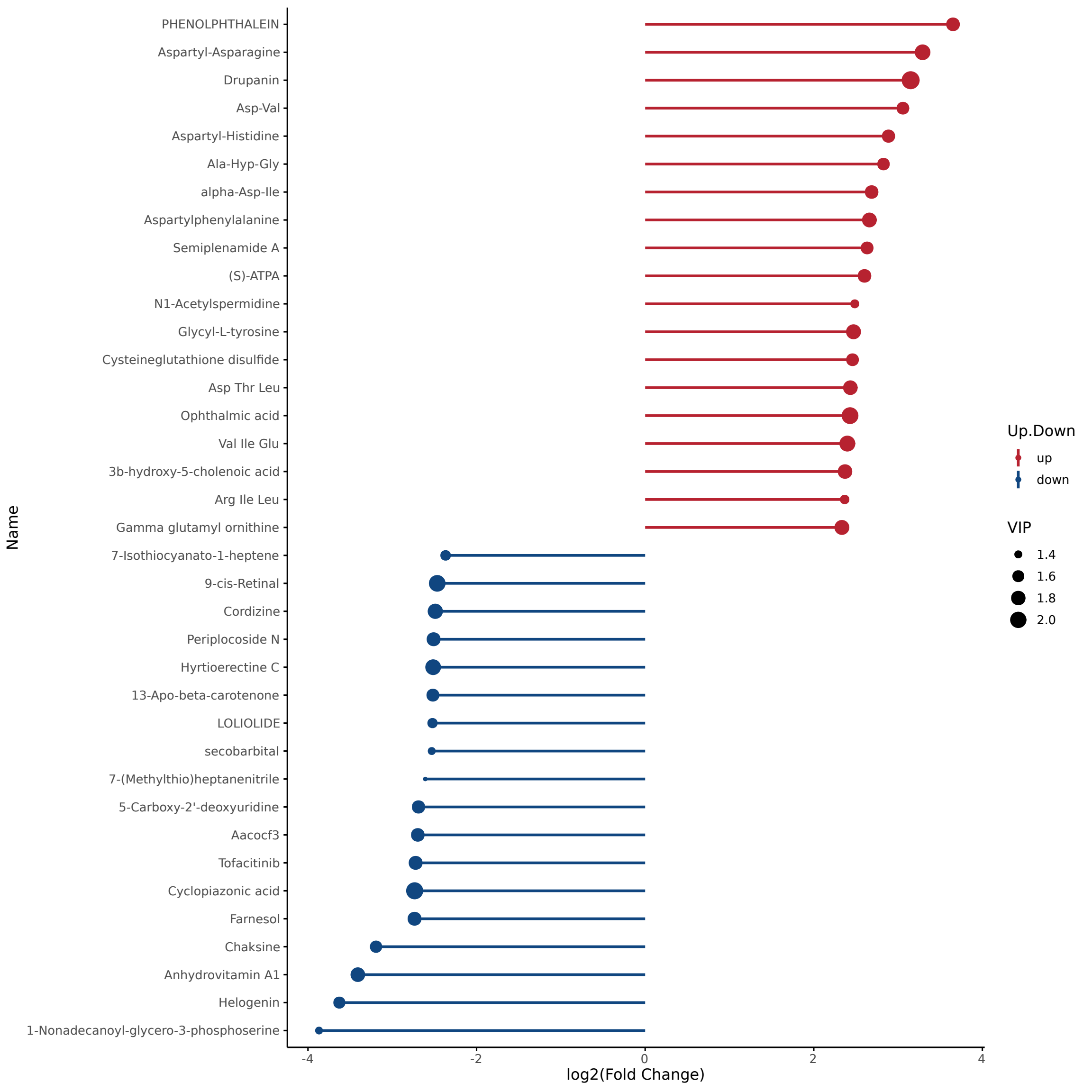

Supplement: Supplementary file 1 [file ijms-27-05895-s001.zip › result/3.MetDiffScreening/B_28d.vs.C_28d/B_28d.vs.C_28d_all_stem.pdf]

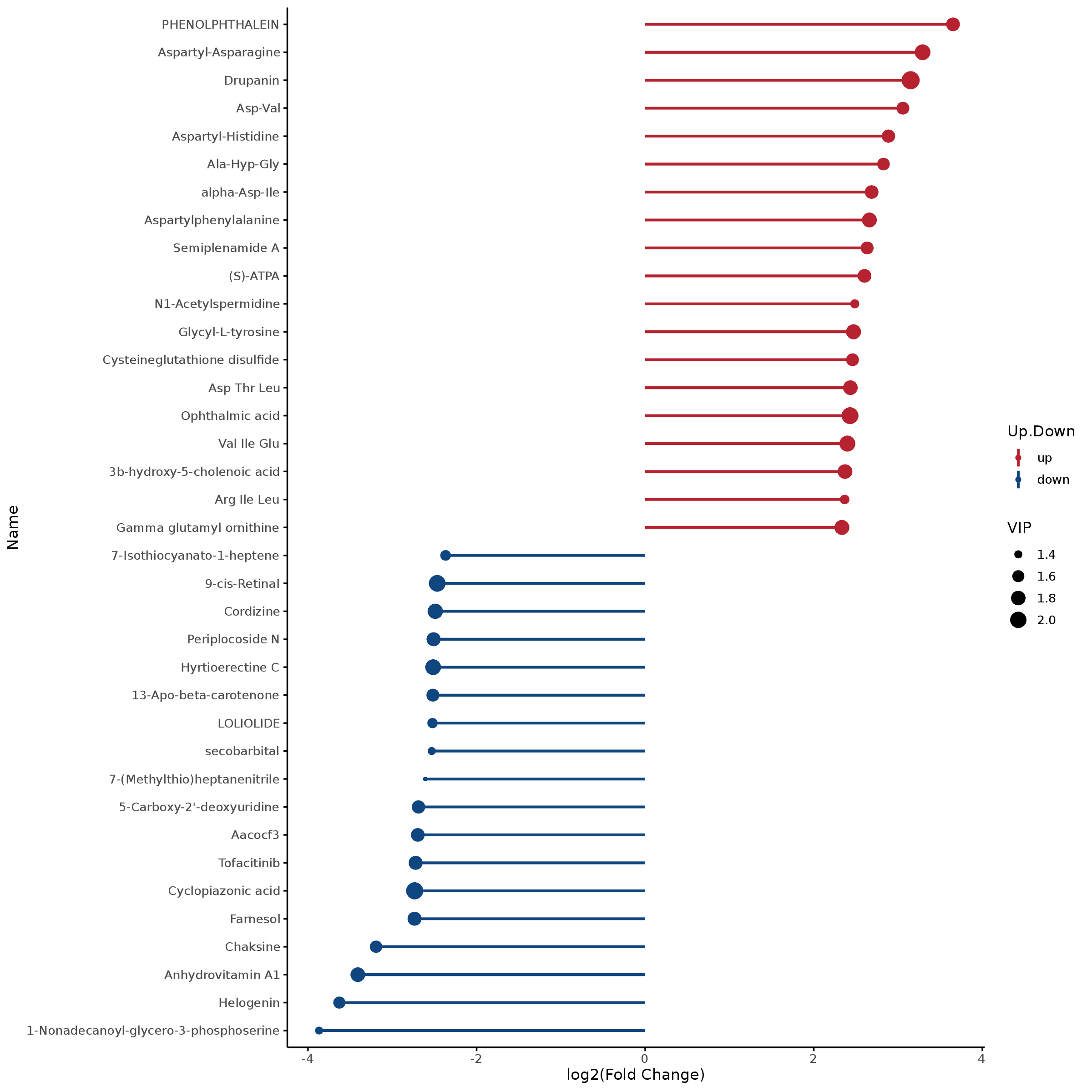

Supplement: Supplementary file 1 [file ijms-27-05895-s001.zip › result/3.MetDiffScreening/B_28d.vs.C_28d/B_28d.vs.C_28d_all_stem.png]

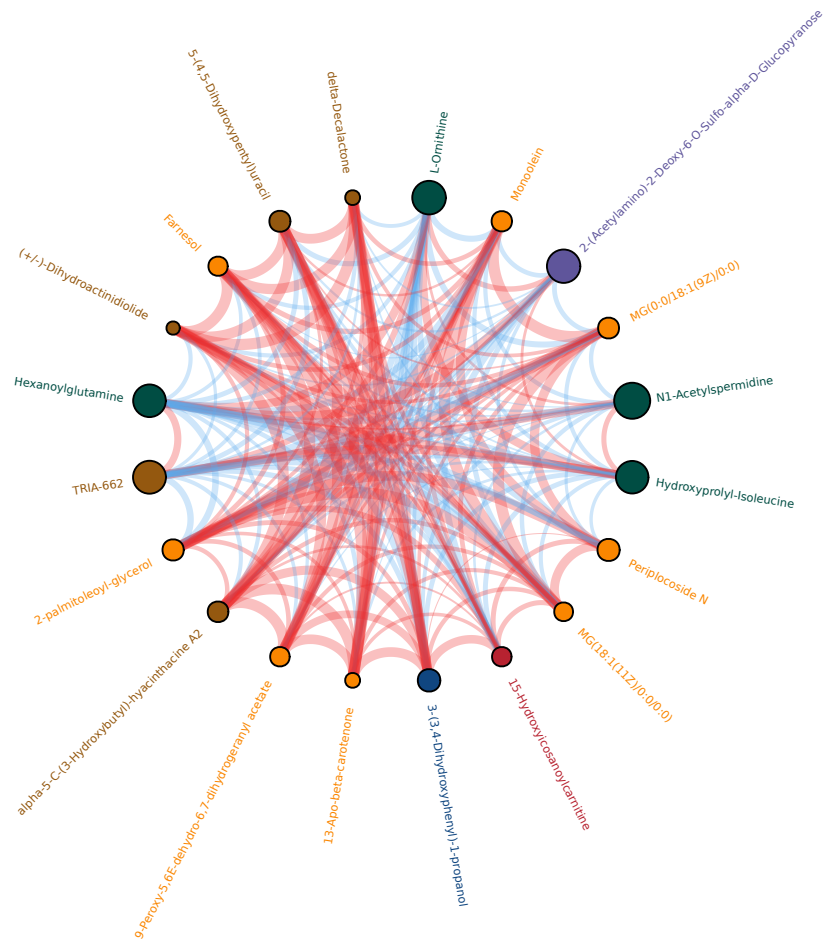

Supplement: Supplementary file 1 [file ijms-27-05895-s001.zip › result/4.MetDiffAnalysis/B_14d.vs.C_14d/B_14d.vs.C_14d_all_chord.pdf]

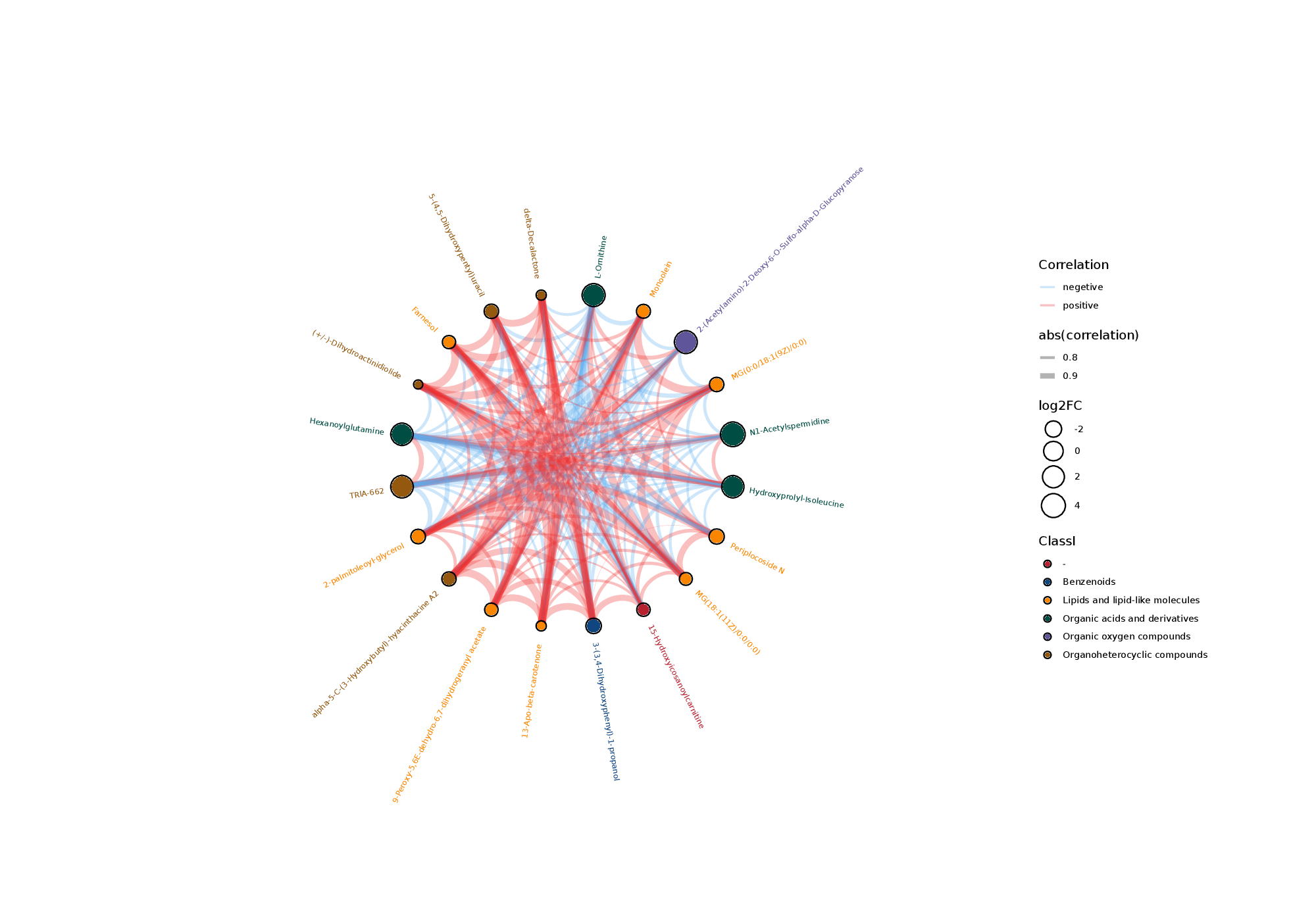

Supplement: Supplementary file 1 [file ijms-27-05895-s001.zip › result/4.MetDiffAnalysis/B_14d.vs.C_14d/B_14d.vs.C_14d_all_chord.png]

B\_14d.vs.C\_14d

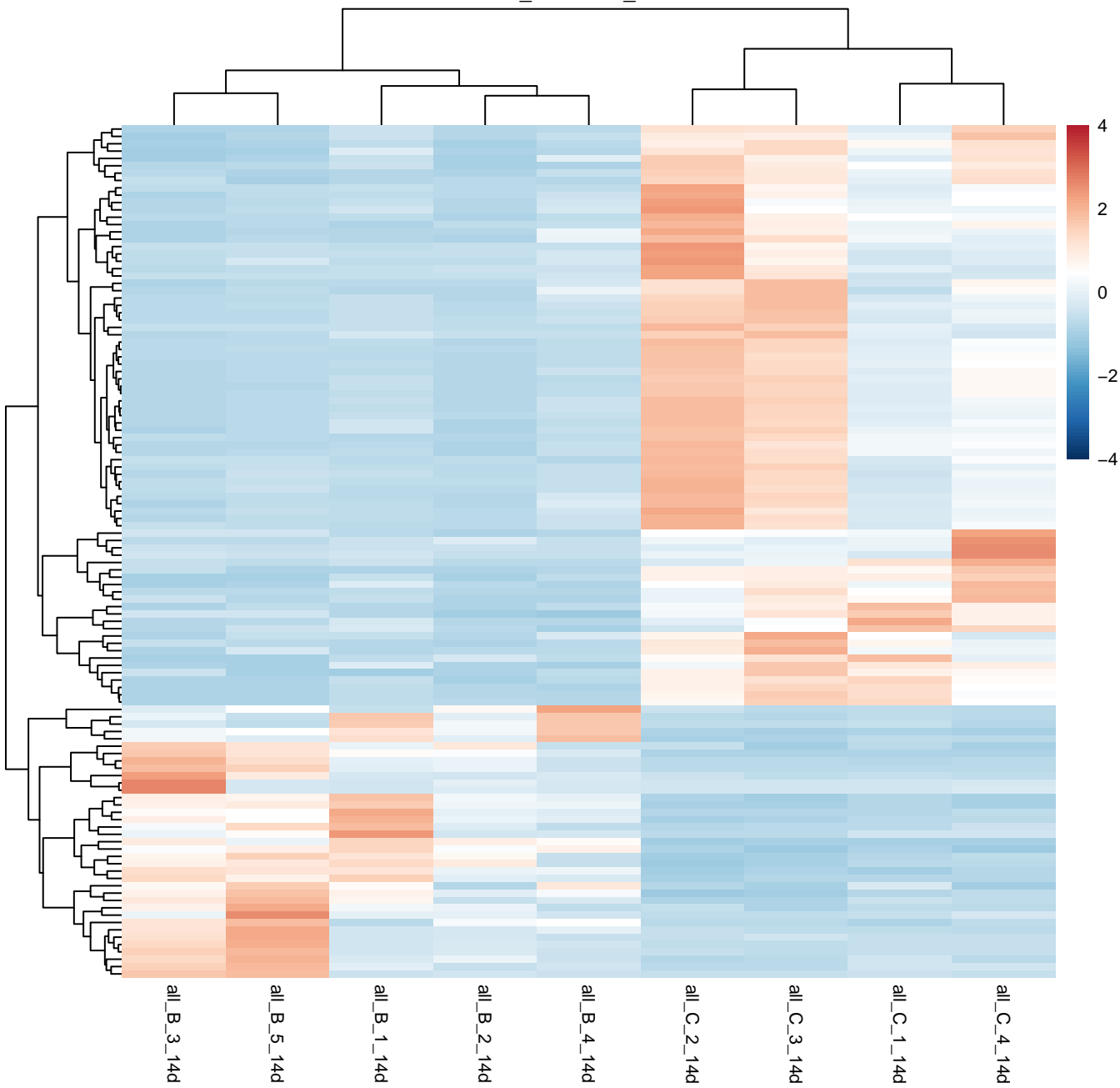

Supplement: Supplementary file 1 [file ijms-27-05895-s001.zip › result/4.MetDiffAnalysis/B_14d.vs.C_14d/B_14d.vs.C_14d_all_cluster_heatmap.pdf]

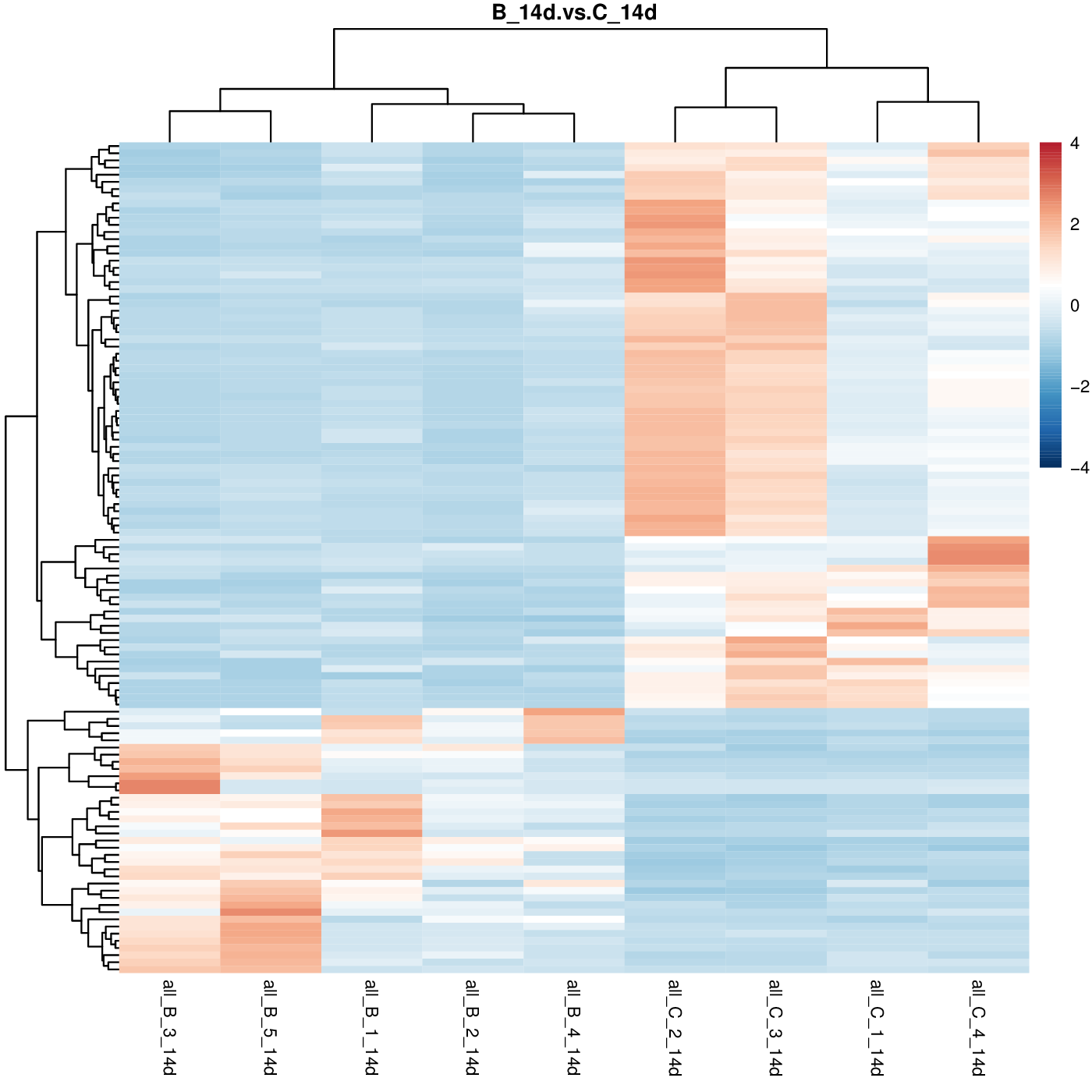

Supplement: Supplementary file 1 [file ijms-27-05895-s001.zip › result/4.MetDiffAnalysis/B_14d.vs.C_14d/B_14d.vs.C_14d_all_cluster_heatmap.png]

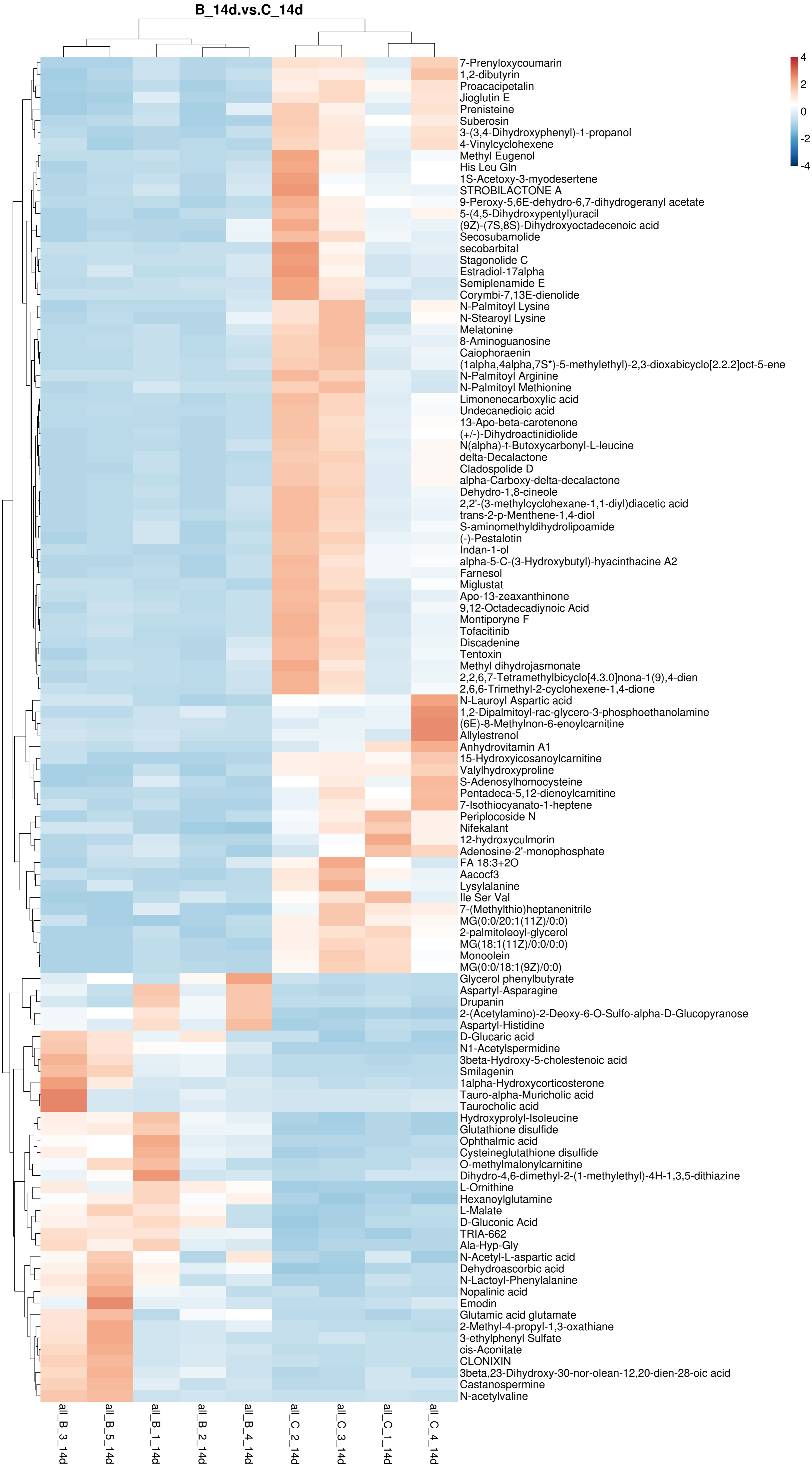

Supplement: Supplementary file 1 [file ijms-27-05895-s001.zip › result/4.MetDiffAnalysis/B_14d.vs.C_14d/B_14d.vs.C_14d_all_cluster_heatmap_detail.pdf]

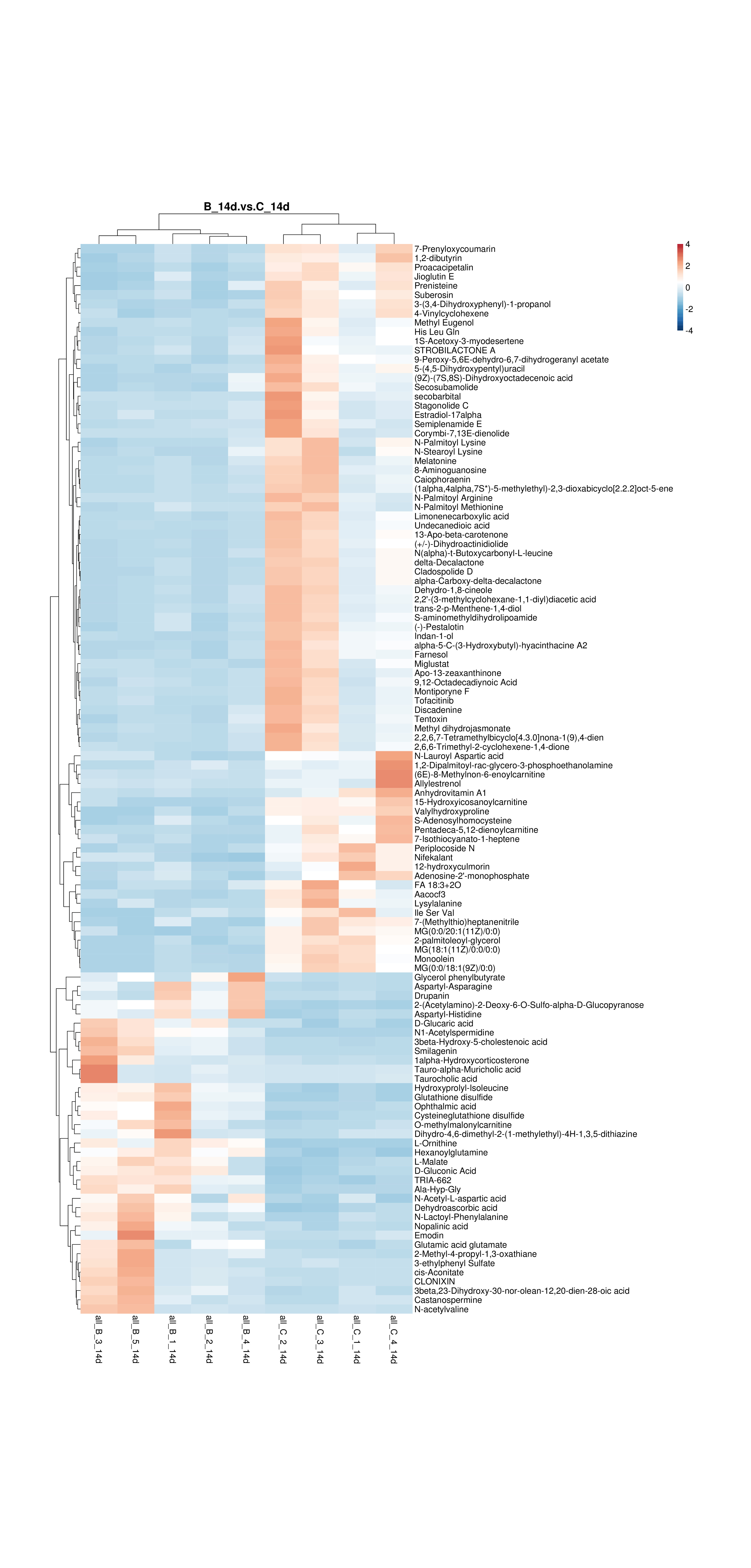

Supplement: Supplementary file 1 [file ijms-27-05895-s001.zip › result/4.MetDiffAnalysis/B_14d.vs.C_14d/B_14d.vs.C_14d_all_cluster_heatmap_detail.png]

## B\_14d.vs.C\_14d

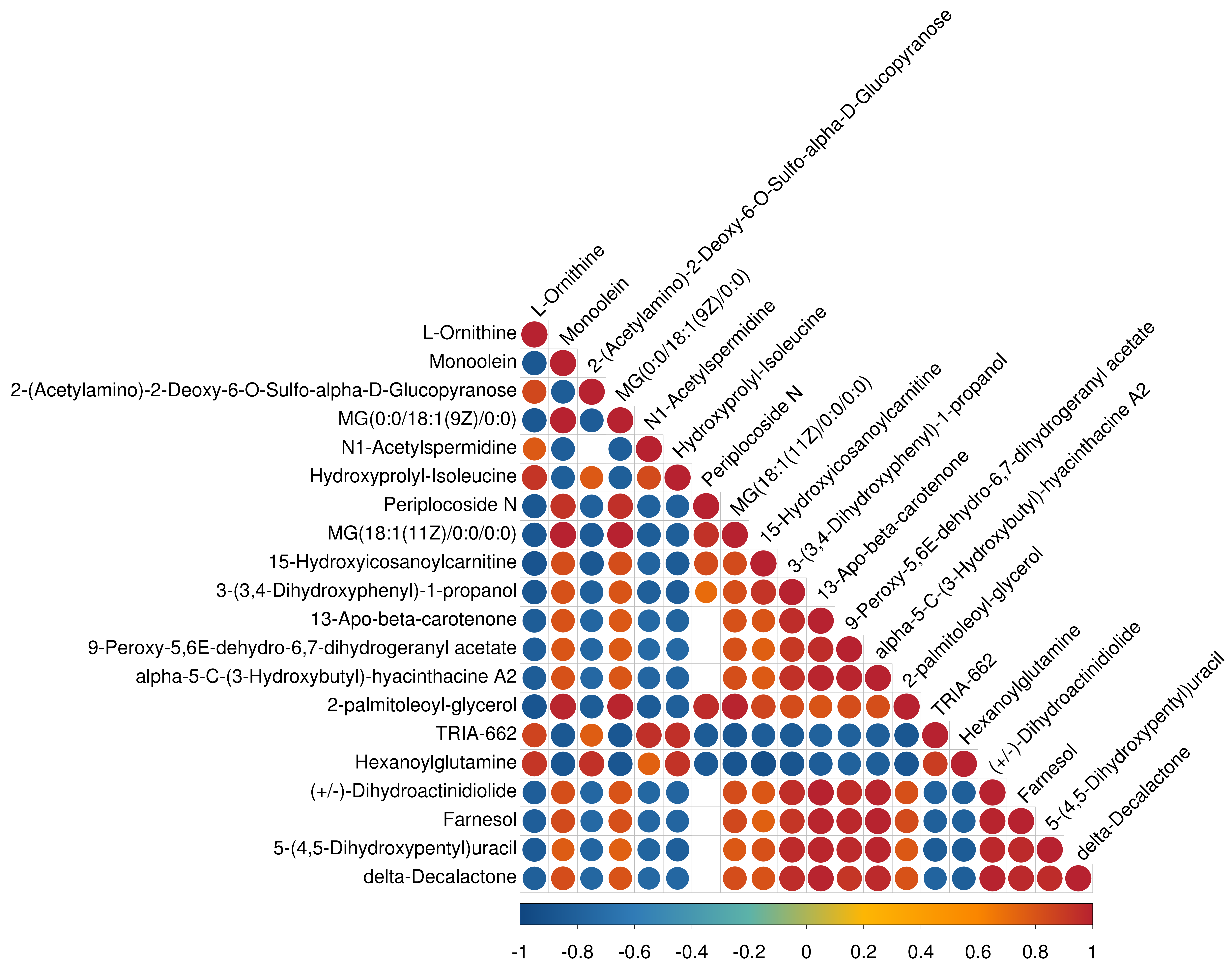

Supplement: Supplementary file 1 [file ijms-27-05895-s001.zip › result/4.MetDiffAnalysis/B_14d.vs.C_14d/B_14d.vs.C_14d_all_corr.pdf]

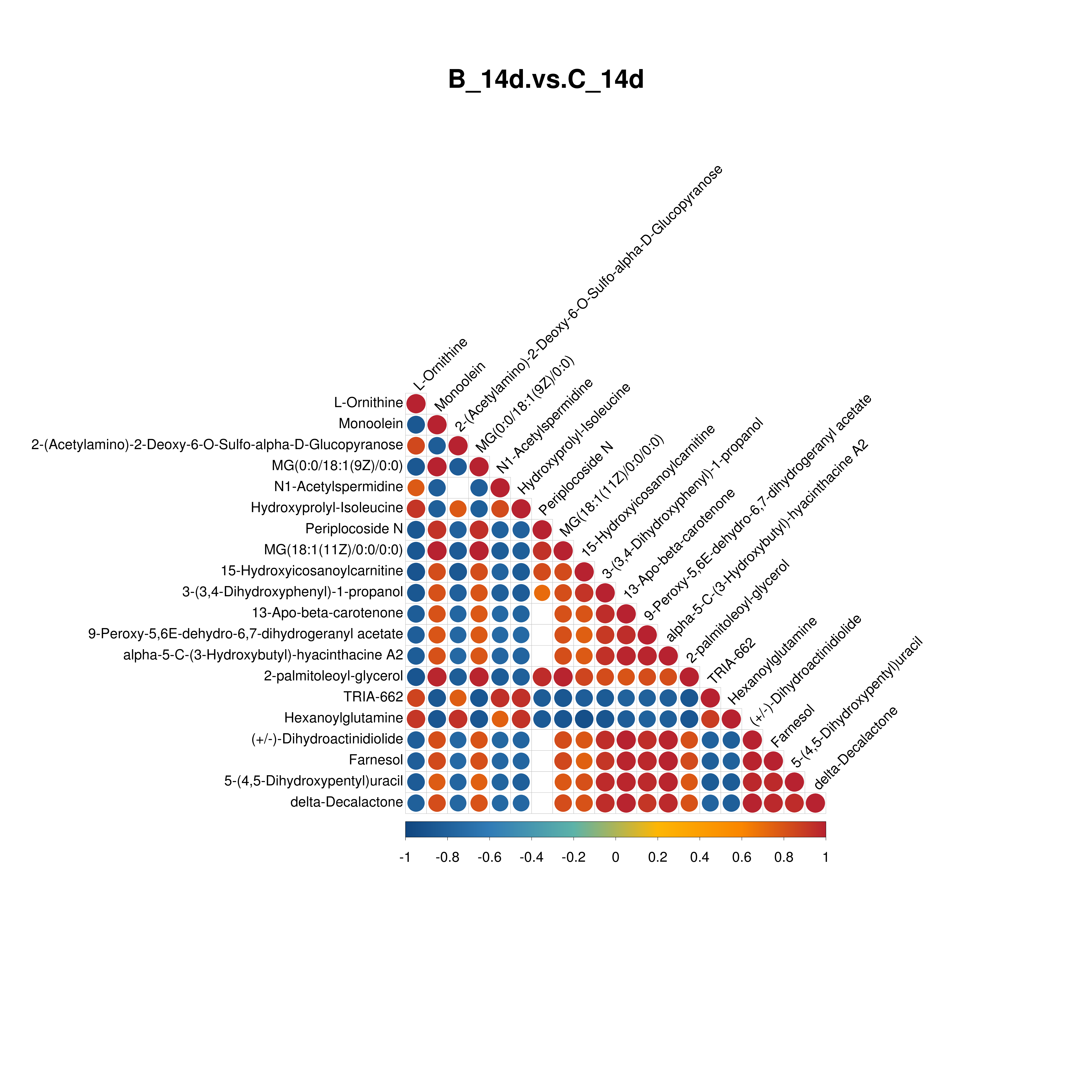

Supplement: Supplementary file 1 [file ijms-27-05895-s001.zip › result/4.MetDiffAnalysis/B_14d.vs.C_14d/B_14d.vs.C_14d_all_corr.png]

B\_14d.vs.C\_14d

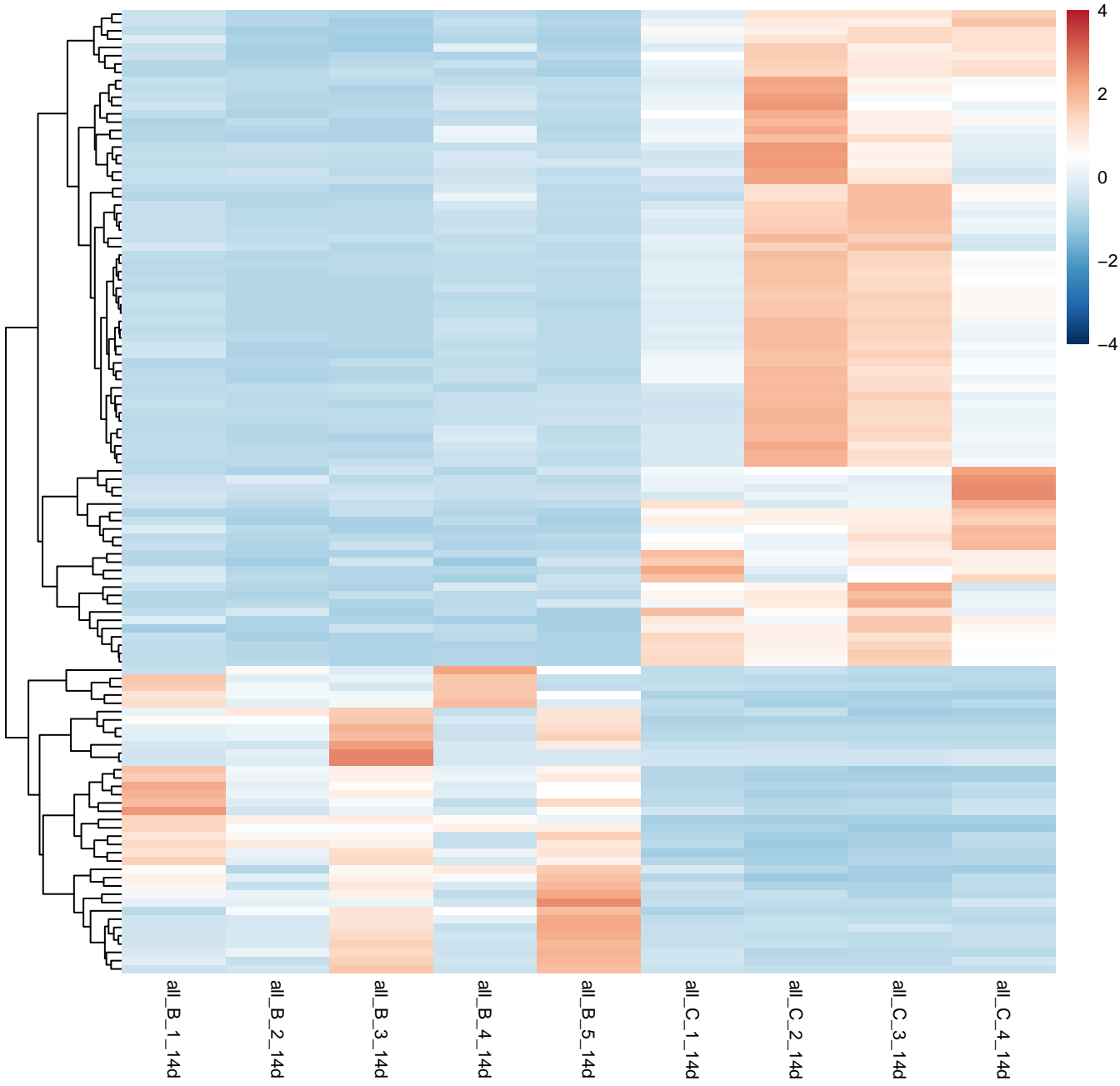

Supplement: Supplementary file 1 [file ijms-27-05895-s001.zip › result/4.MetDiffAnalysis/B_14d.vs.C_14d/B_14d.vs.C_14d_all_heatmap.pdf]

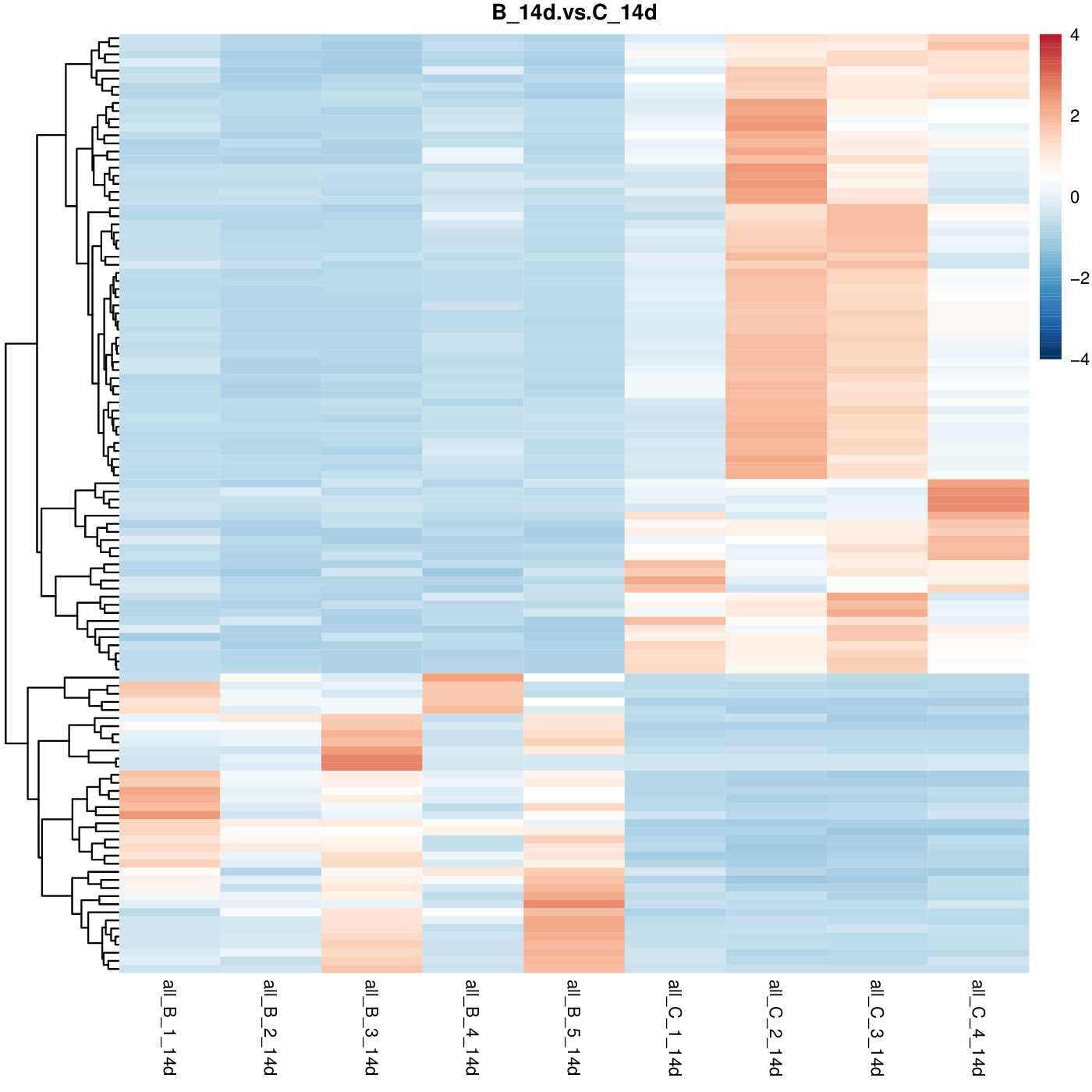

Supplement: Supplementary file 1 [file ijms-27-05895-s001.zip › result/4.MetDiffAnalysis/B_14d.vs.C_14d/B_14d.vs.C_14d_all_heatmap.png]

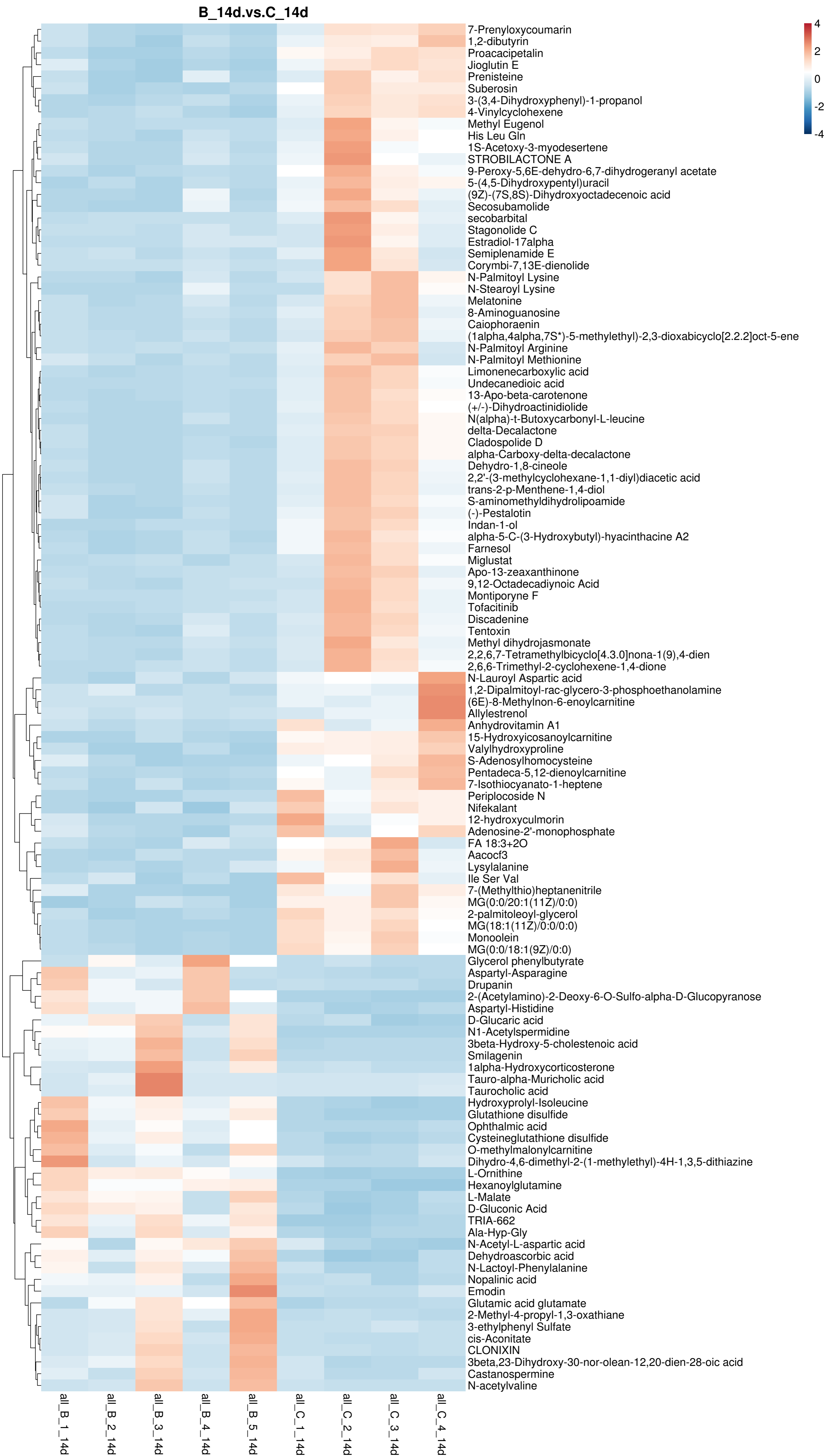

Supplement: Supplementary file 1 [file ijms-27-05895-s001.zip › result/4.MetDiffAnalysis/B_14d.vs.C_14d/B_14d.vs.C_14d_all_heatmap_detail.pdf]

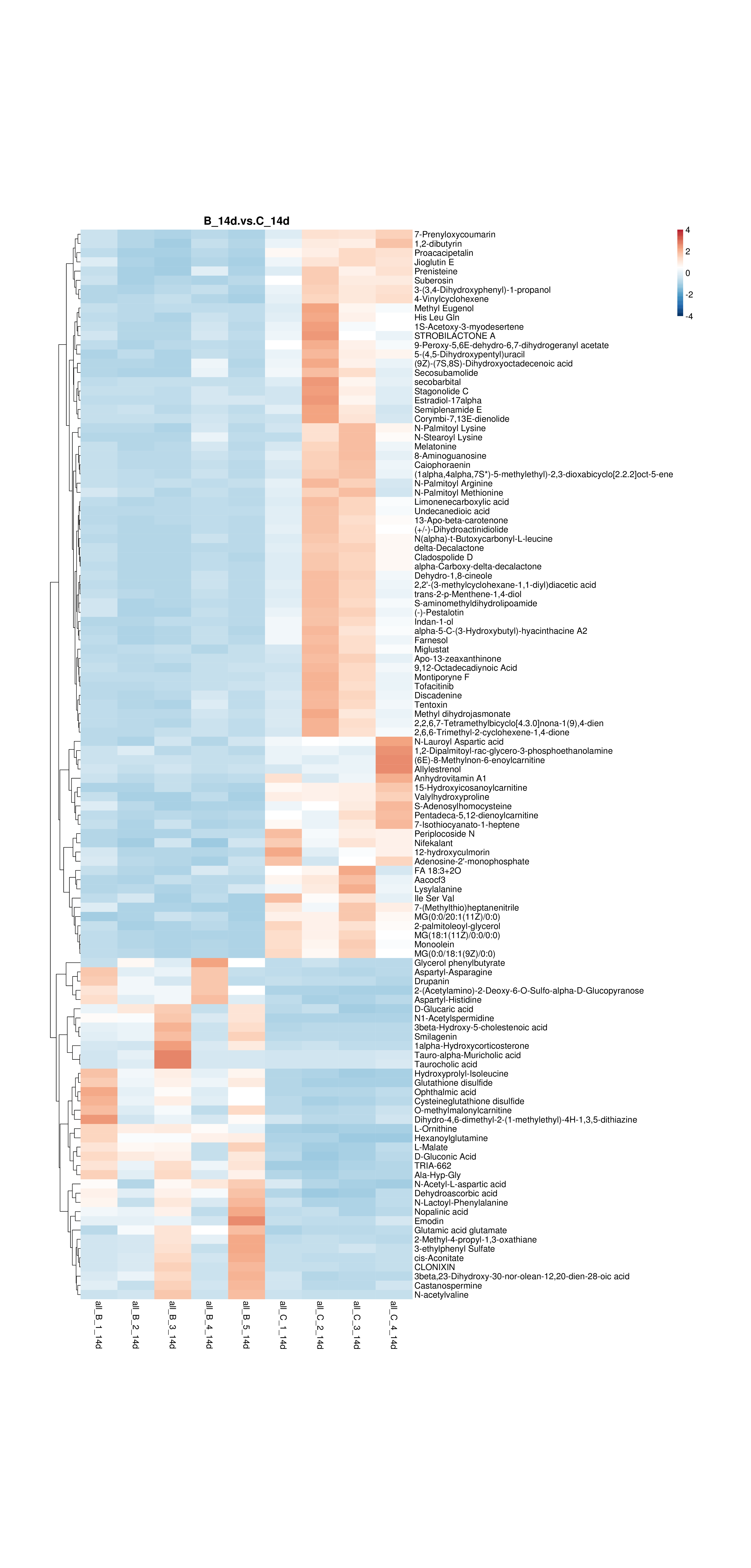

Supplement: Supplementary file 1 [file ijms-27-05895-s001.zip › result/4.MetDiffAnalysis/B_14d.vs.C_14d/B_14d.vs.C_14d_all_heatmap_detail.png]

B\_14d.vs.C\_14d

Metabolites

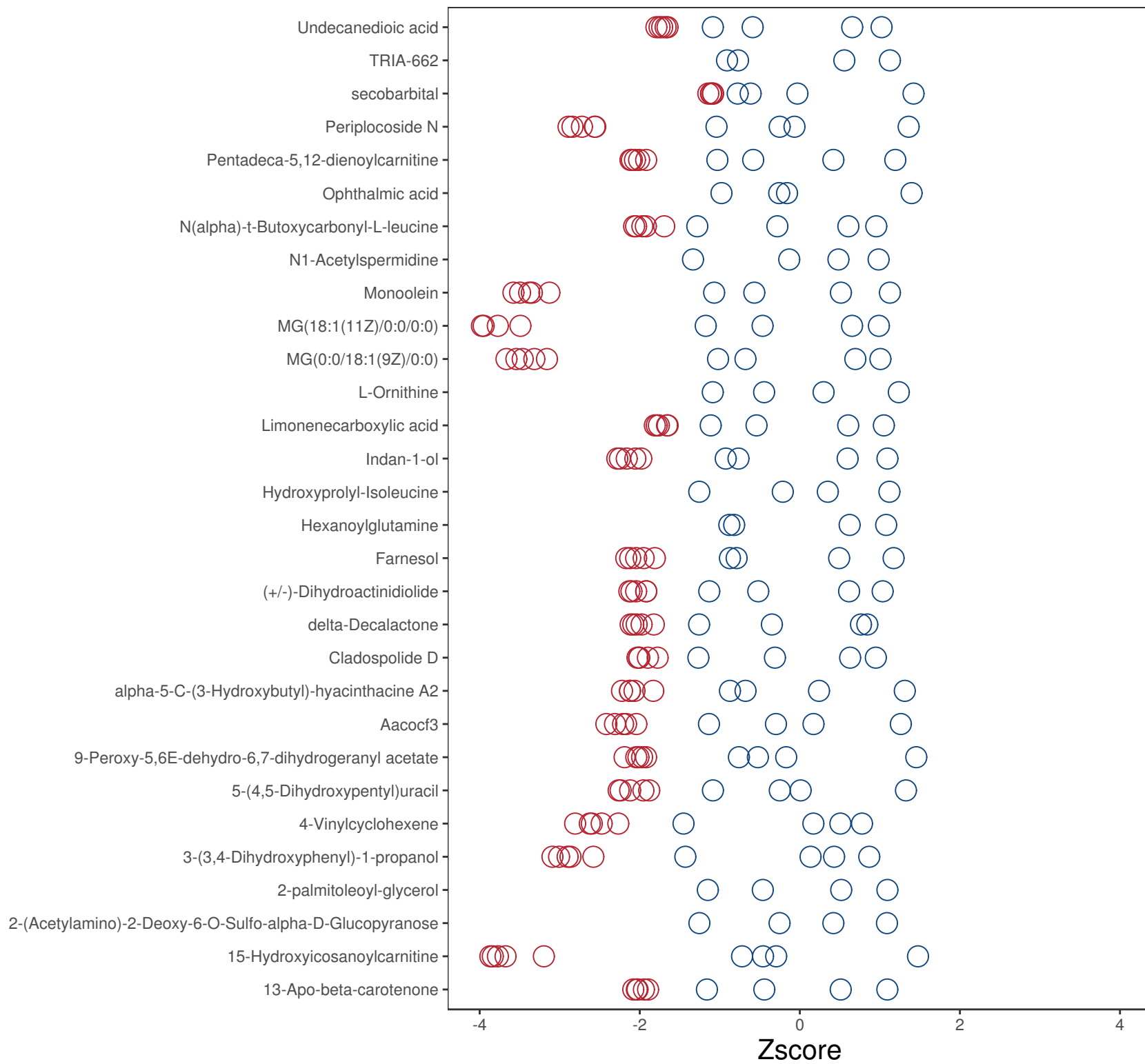

Group

B\_14d  
C\_14d

Supplement: Supplementary file 1 [file ijms-27-05895-s001.zip › result/4.MetDiffAnalysis/B_14d.vs.C_14d/B_14d.vs.C_14d_all_zscore.pdf]

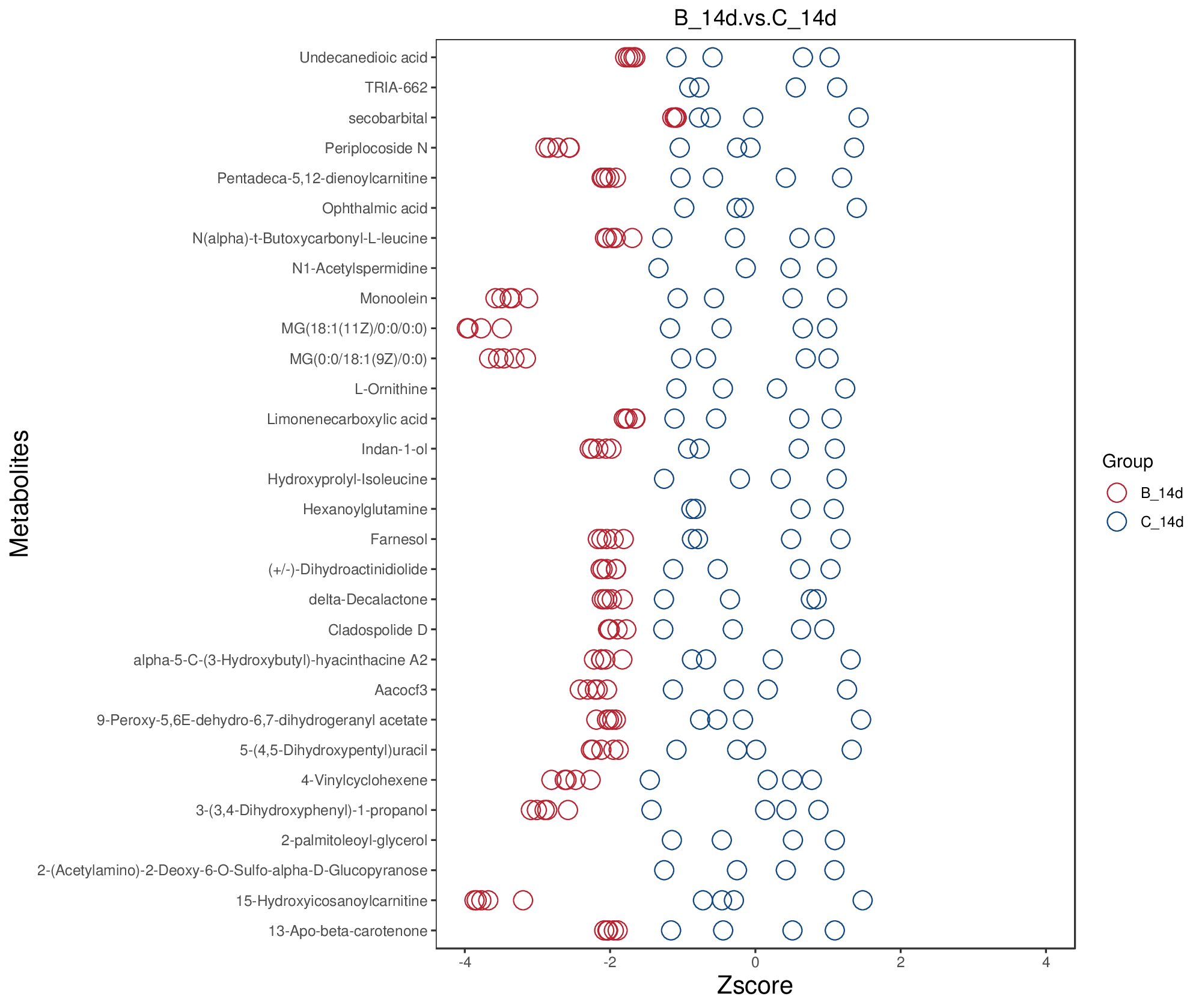

Supplement: Supplementary file 1 [file ijms-27-05895-s001.zip › result/4.MetDiffAnalysis/B_14d.vs.C_14d/B_14d.vs.C_14d_all_zscore.png]

B\_14d.vs.C\_14d

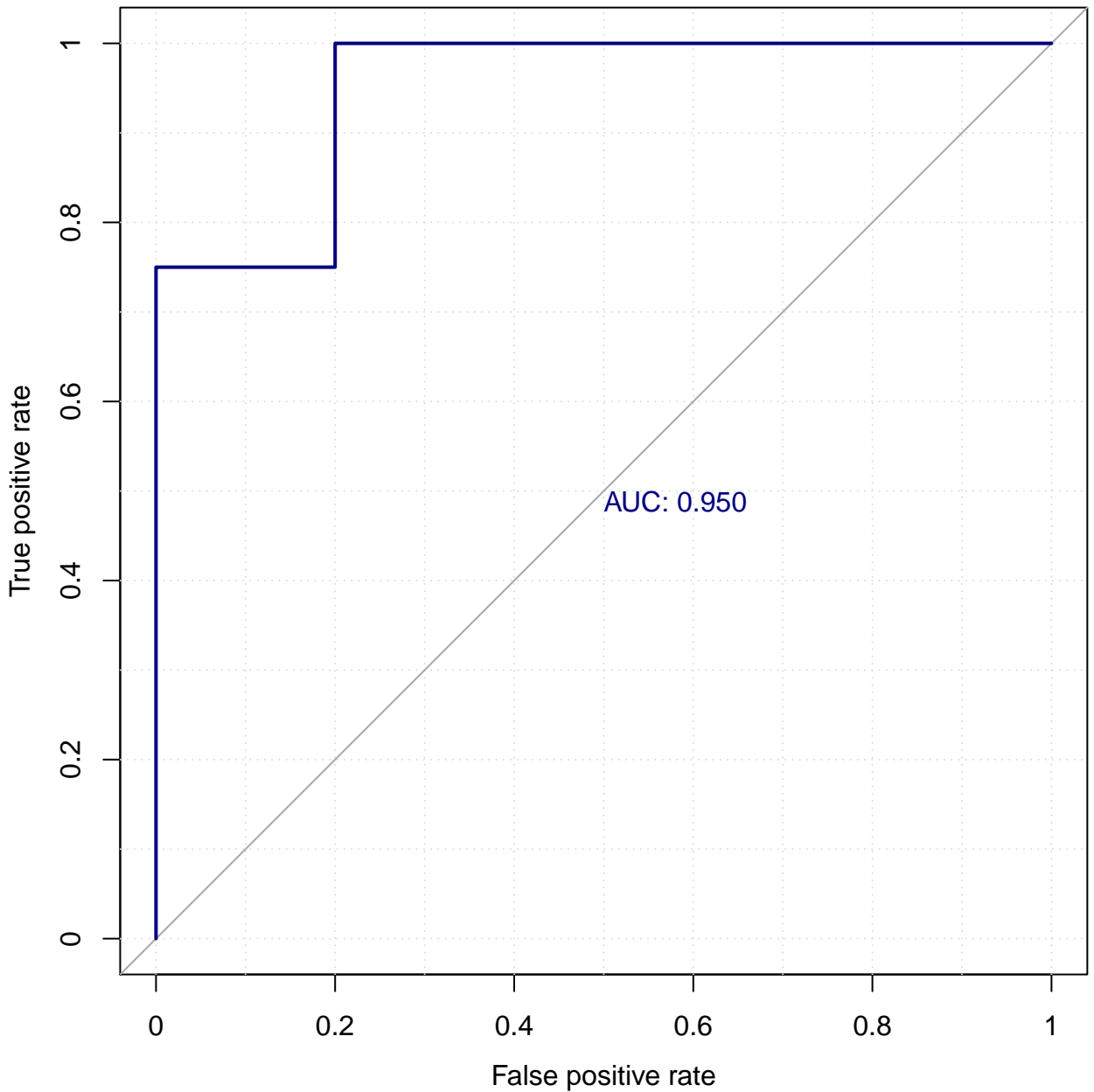

Supplement: Supplementary file 1 [file ijms-27-05895-s001.zip › result/4.MetDiffAnalysis/B_14d.vs.C_14d/ROC_all/Com_1022_pos_ROC.pdf]

B\_14d.vs.C\_14d

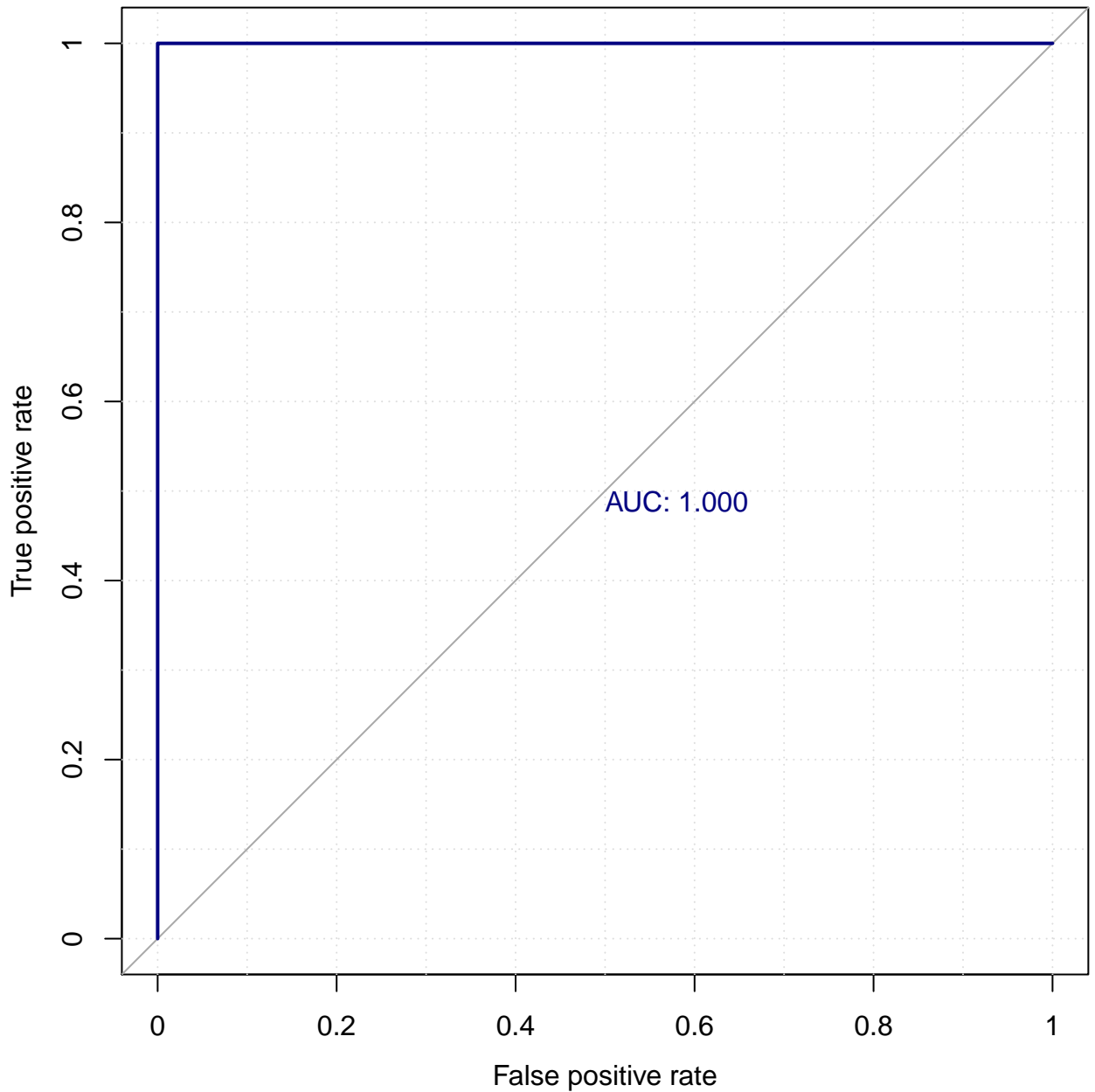

Supplement: Supplementary file 1 [file ijms-27-05895-s001.zip › result/4.MetDiffAnalysis/B_14d.vs.C_14d/ROC_all/Com_1067_pos_ROC.pdf]

B\_14d.vs.C\_14d

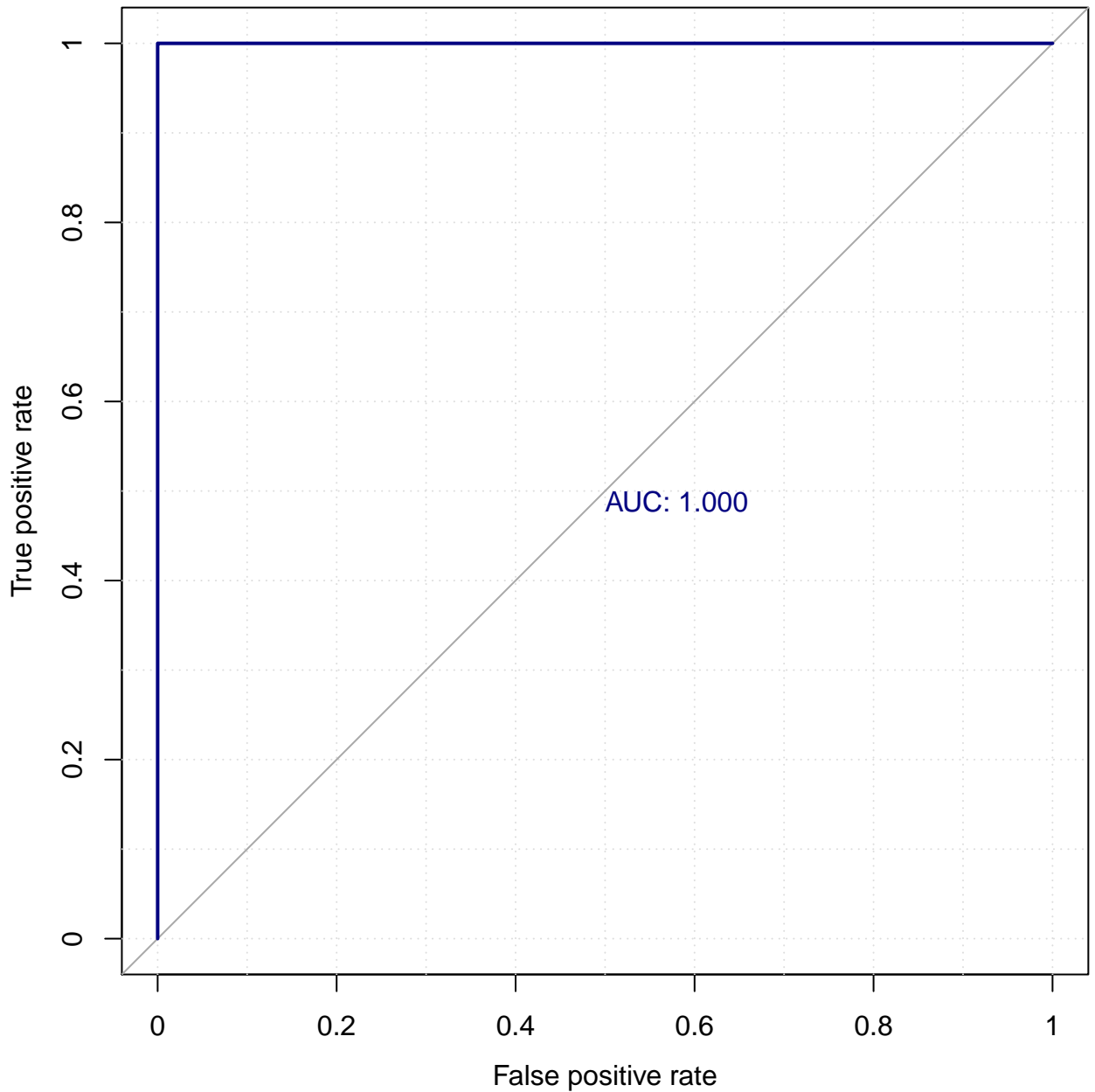

Supplement: Supplementary file 1 [file ijms-27-05895-s001.zip › result/4.MetDiffAnalysis/B_14d.vs.C_14d/ROC_all/Com_1120_pos_ROC.pdf]

B\_14d.vs.C\_14d

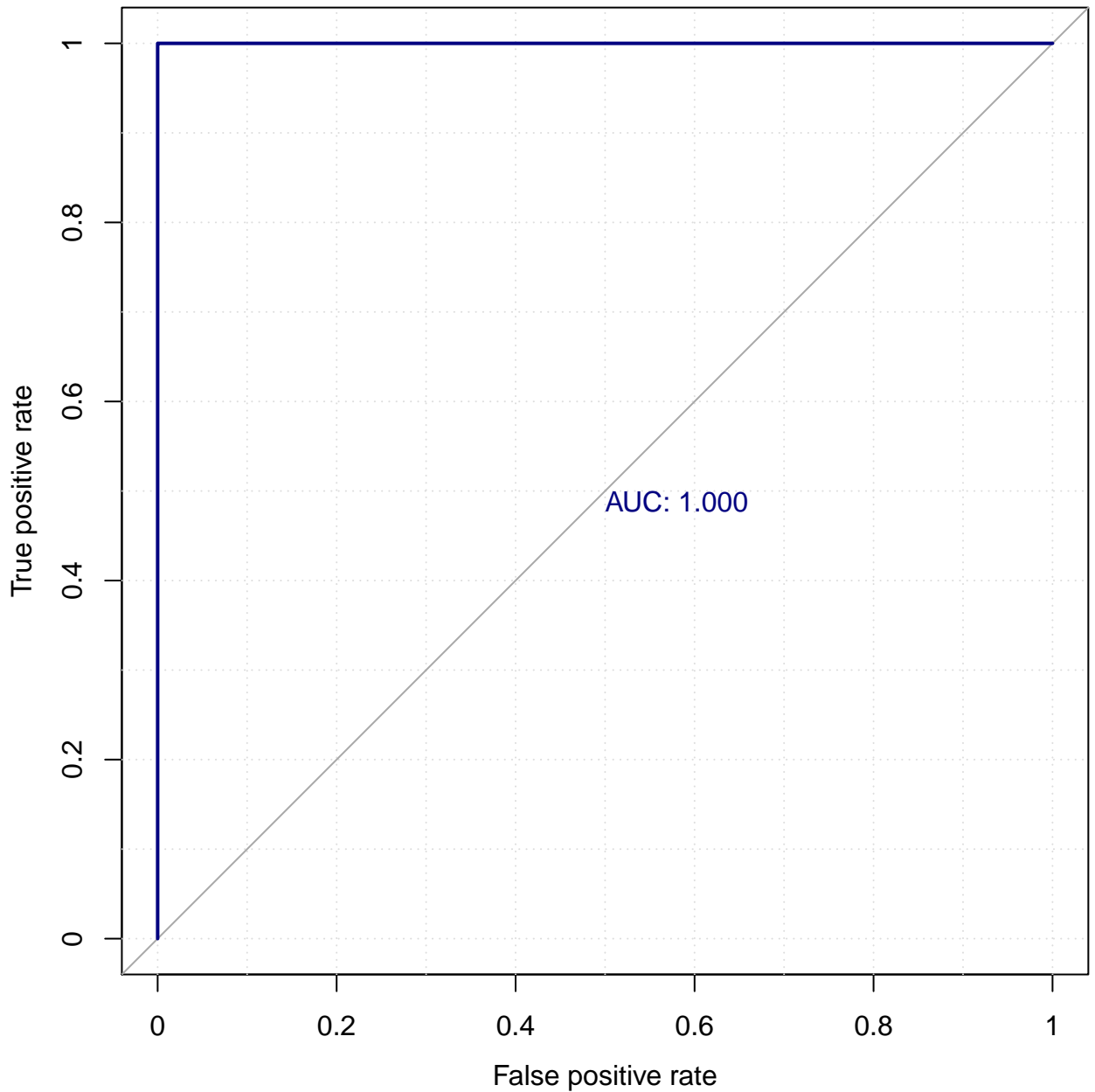

Supplement: Supplementary file 1 [file ijms-27-05895-s001.zip › result/4.MetDiffAnalysis/B_14d.vs.C_14d/ROC_all/Com_1161_pos_ROC.pdf]

B\_14d.vs.C\_14d

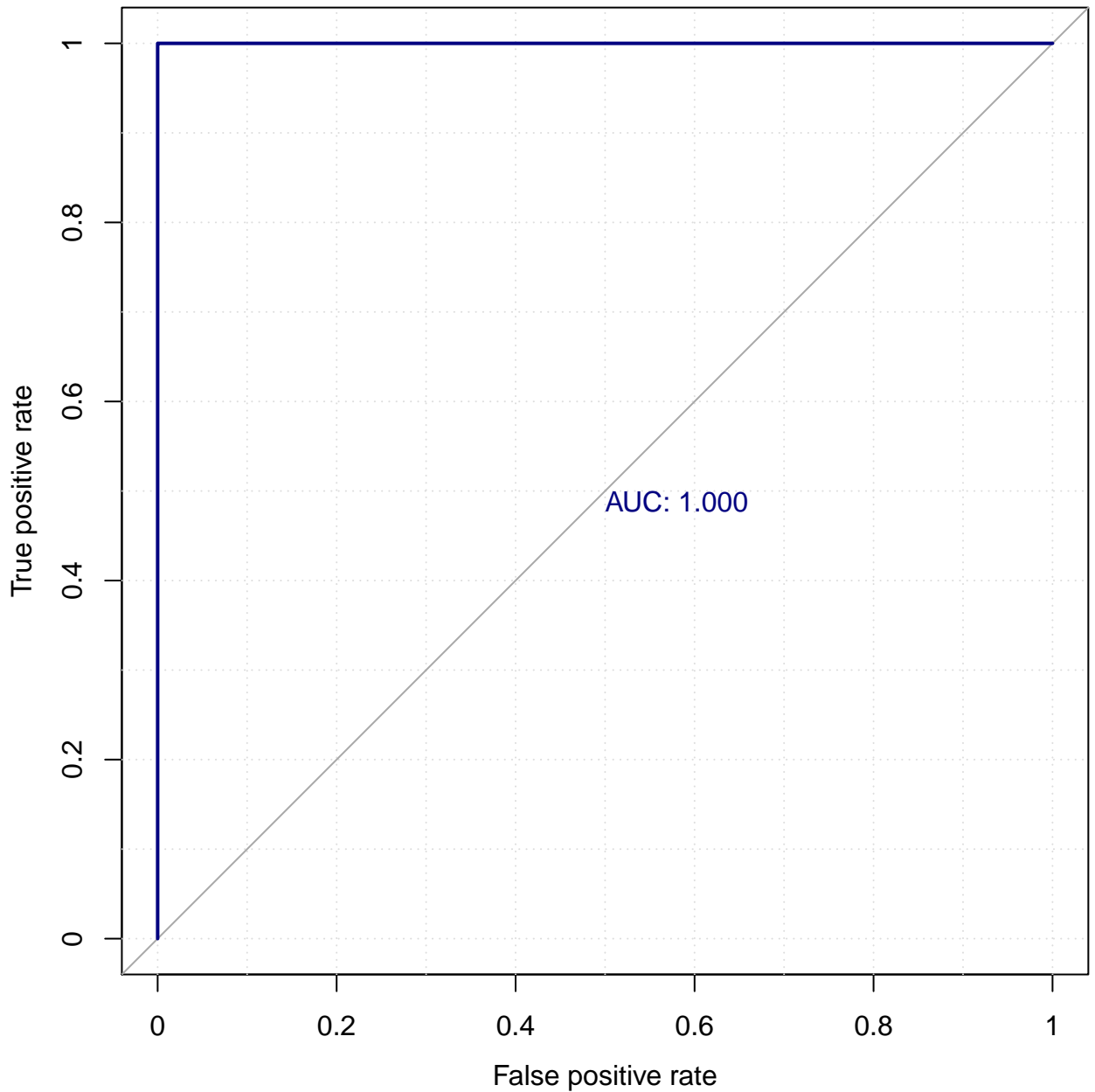

Supplement: Supplementary file 1 [file ijms-27-05895-s001.zip › result/4.MetDiffAnalysis/B_14d.vs.C_14d/ROC_all/Com_1187_pos_ROC.pdf]

B\_14d.vs.C\_14d

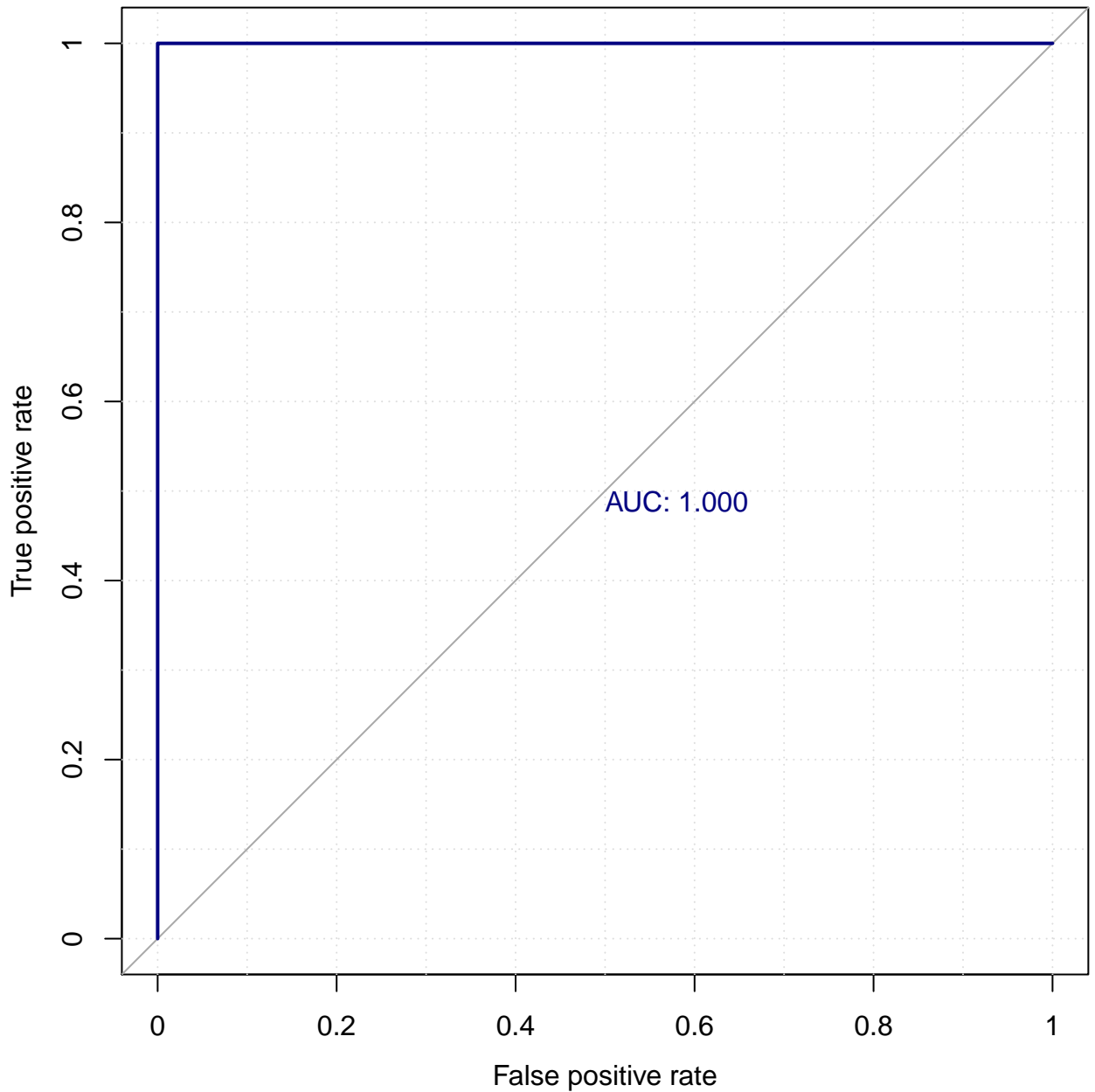

Supplement: Supplementary file 1 [file ijms-27-05895-s001.zip › result/4.MetDiffAnalysis/B_14d.vs.C_14d/ROC_all/Com_1191_neg_ROC.pdf]

B\_14d.vs.C\_14d

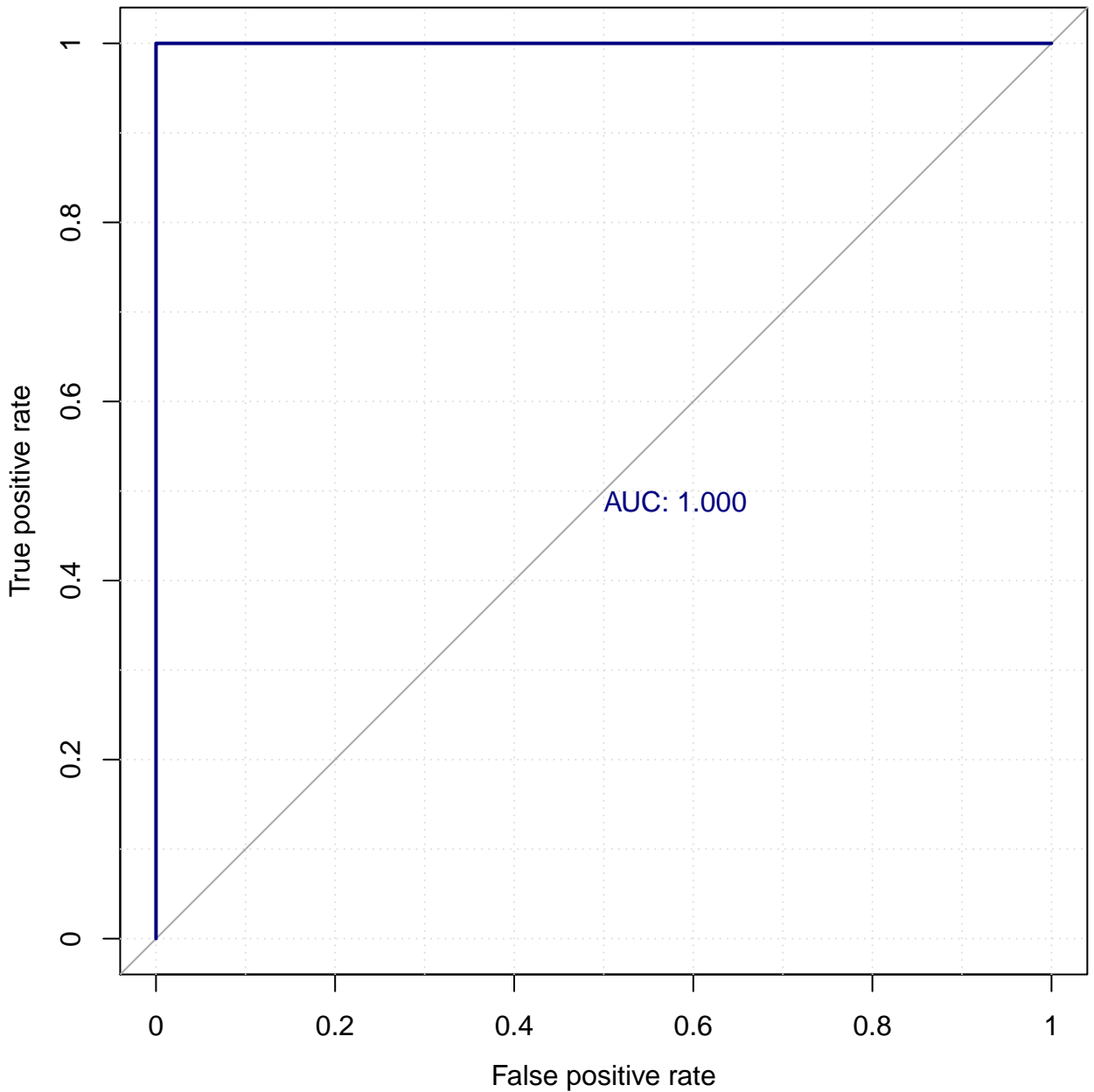

Supplement: Supplementary file 1 [file ijms-27-05895-s001.zip › result/4.MetDiffAnalysis/B_14d.vs.C_14d/ROC_all/Com_1220_pos_ROC.pdf]

B\_14d.vs.C\_14d

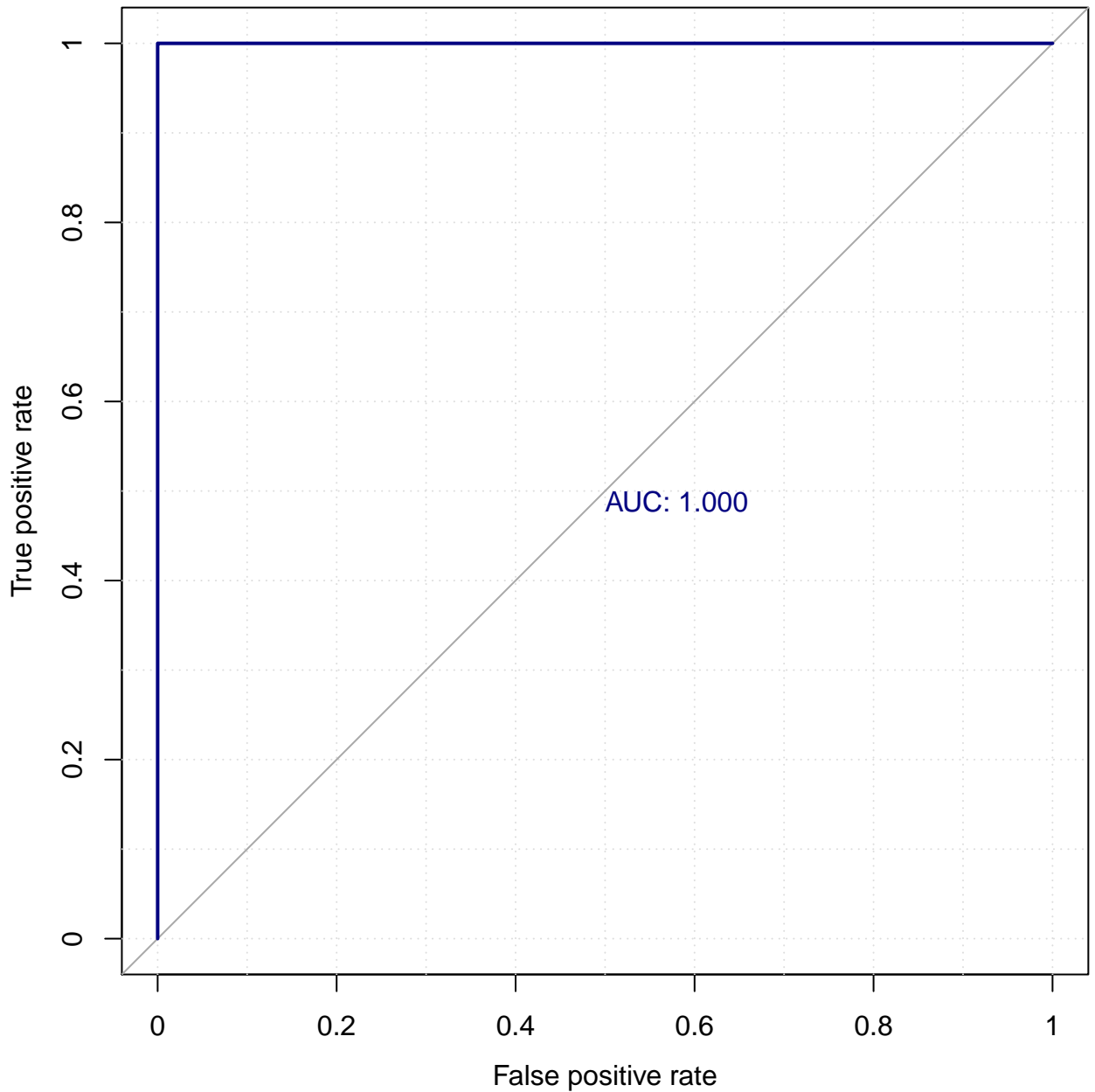

Supplement: Supplementary file 1 [file ijms-27-05895-s001.zip › result/4.MetDiffAnalysis/B_14d.vs.C_14d/ROC_all/Com_122_neg_ROC.pdf]

B\_14d.vs.C\_14d

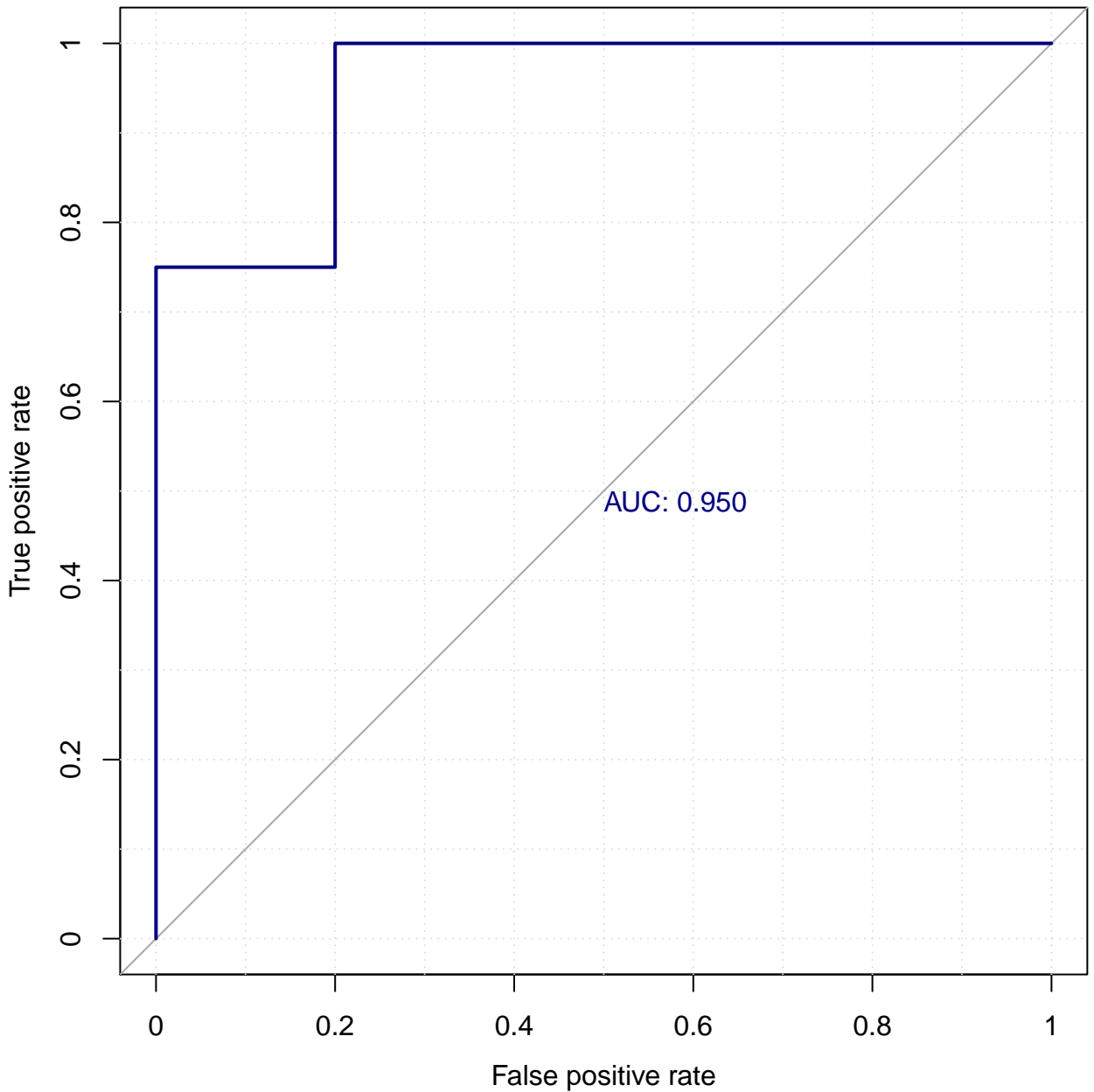

Supplement: Supplementary file 1 [file ijms-27-05895-s001.zip › result/4.MetDiffAnalysis/B_14d.vs.C_14d/ROC_all/Com_1258_pos_ROC.pdf]

B\_14d.vs.C\_14d

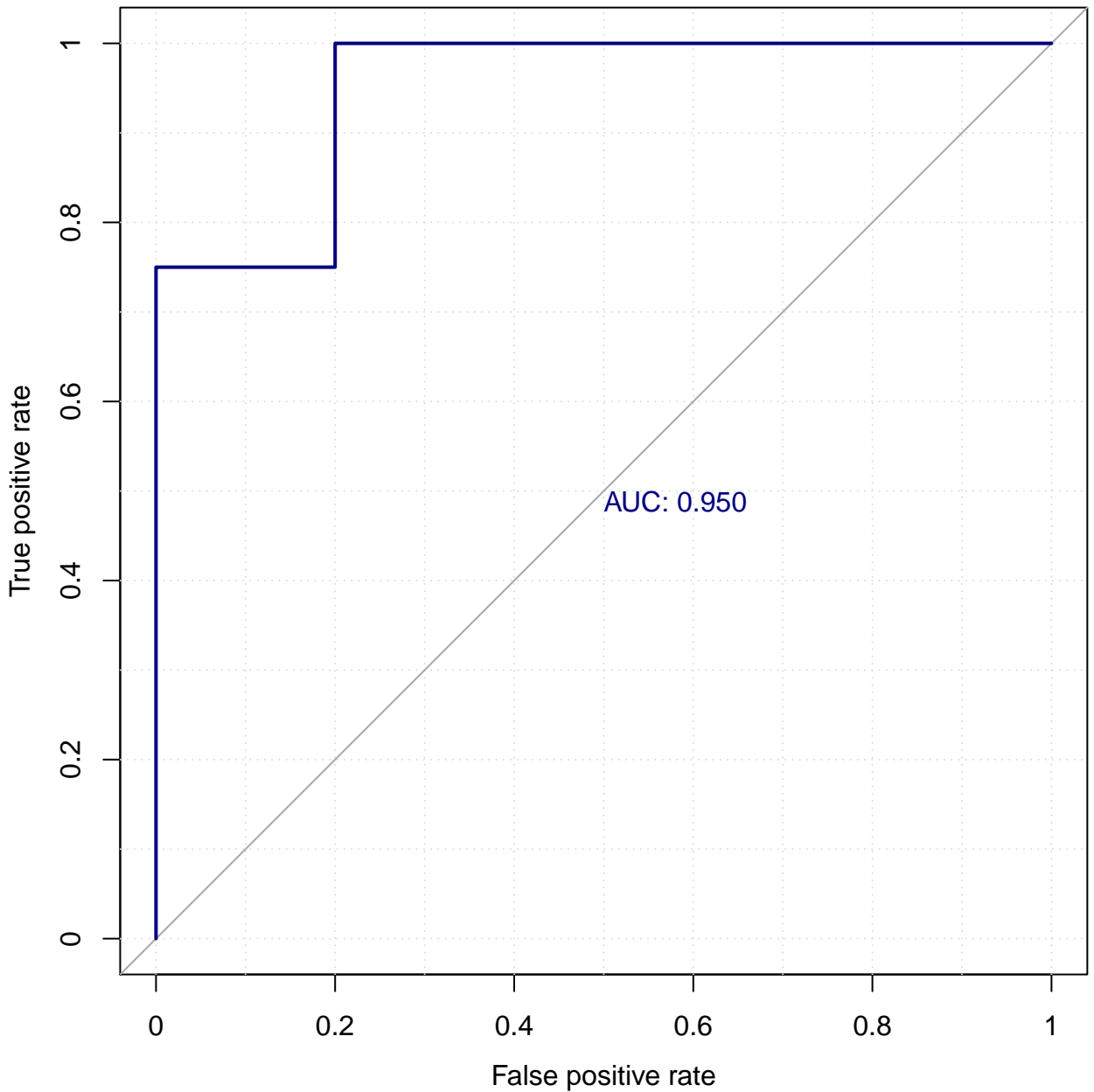

Supplement: Supplementary file 1 [file ijms-27-05895-s001.zip › result/4.MetDiffAnalysis/B_14d.vs.C_14d/ROC_all/Com_1323_neg_ROC.pdf]

B\_14d.vs.C\_14d

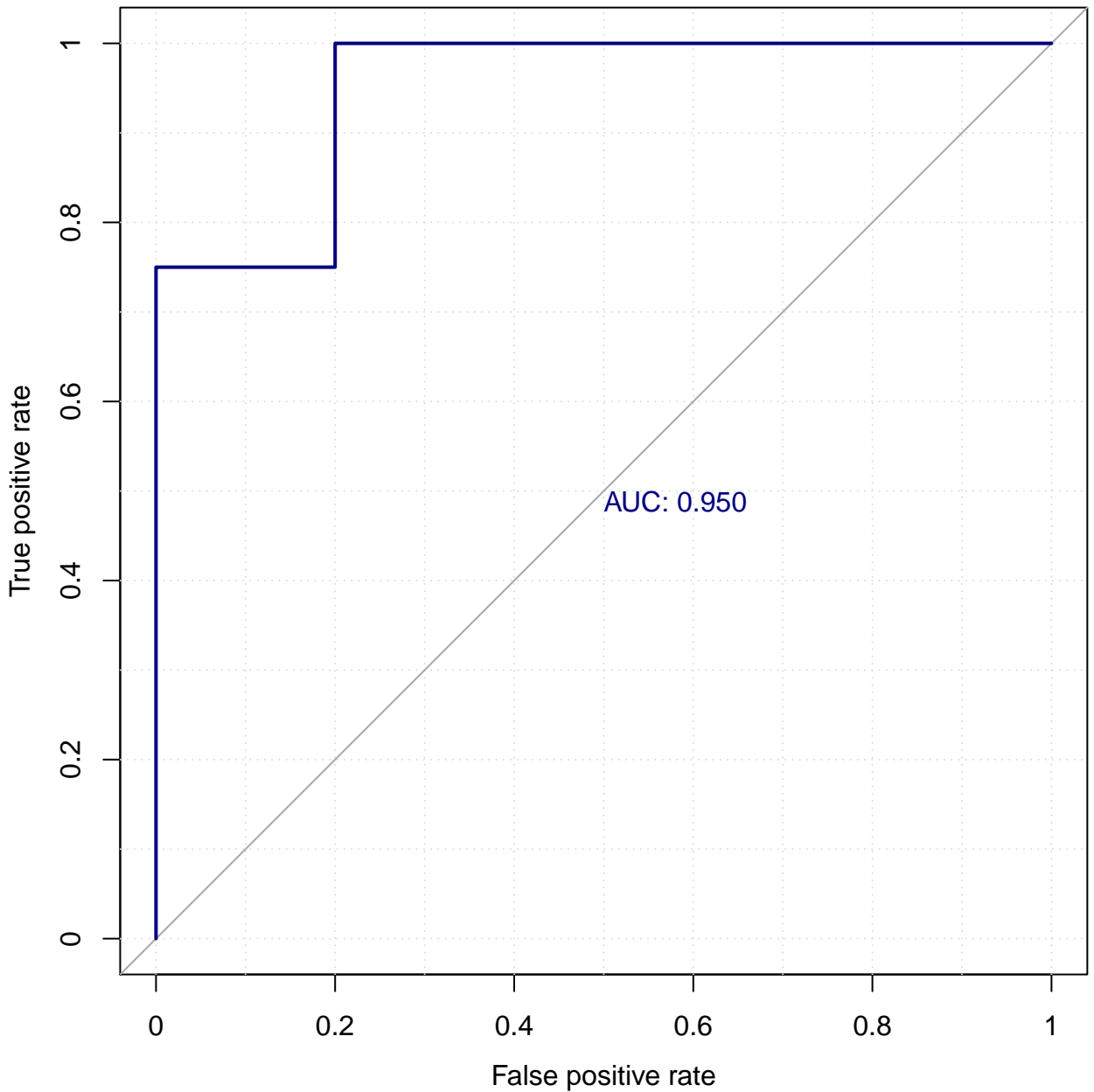

Supplement: Supplementary file 1 [file ijms-27-05895-s001.zip › result/4.MetDiffAnalysis/B_14d.vs.C_14d/ROC_all/Com_1357_neg_ROC.pdf]

B\_14d.vs.C\_14d

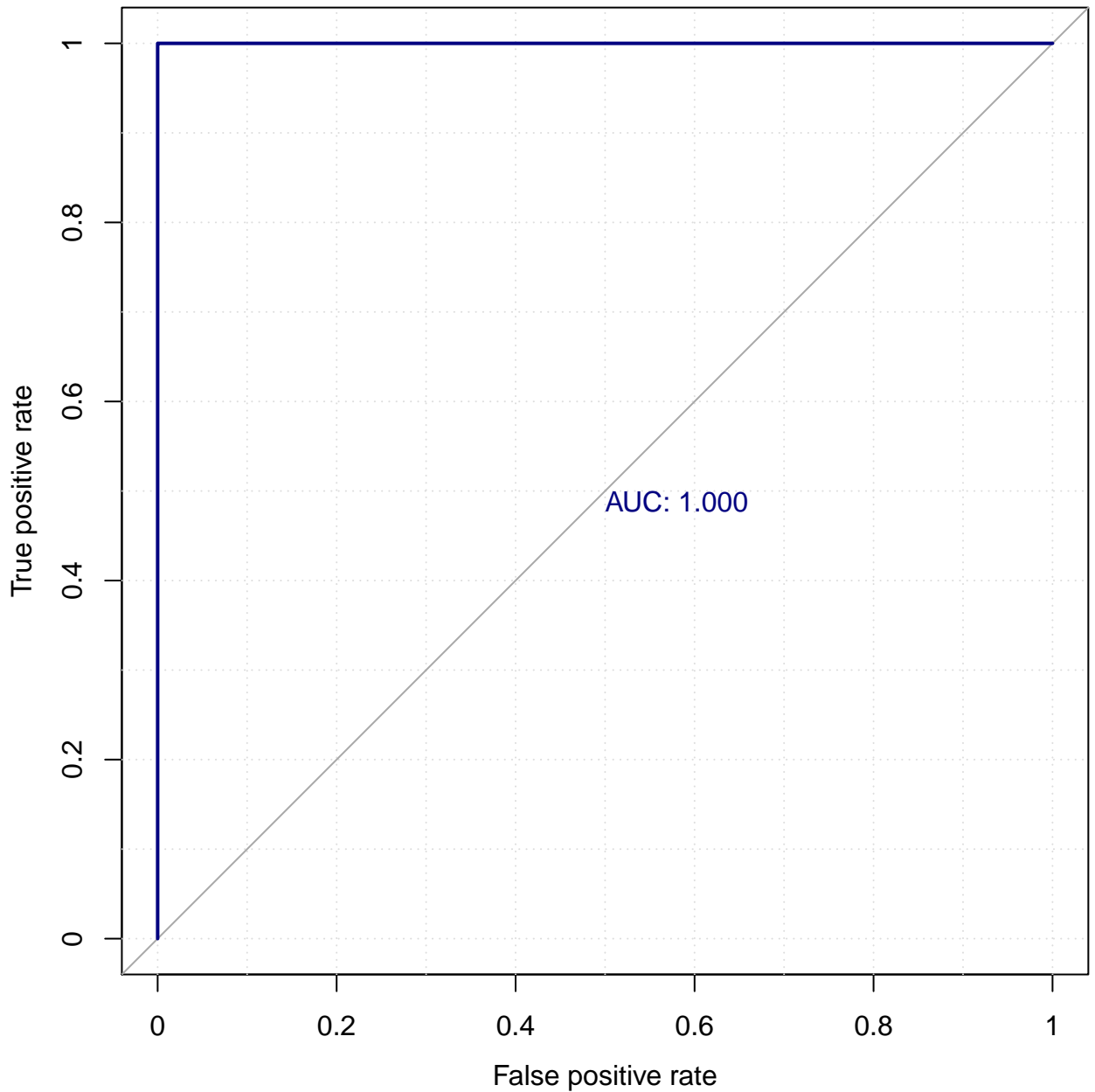

Supplement: Supplementary file 1 [file ijms-27-05895-s001.zip › result/4.MetDiffAnalysis/B_14d.vs.C_14d/ROC_all/Com_1392_pos_ROC.pdf]

B\_14d.vs.C\_14d

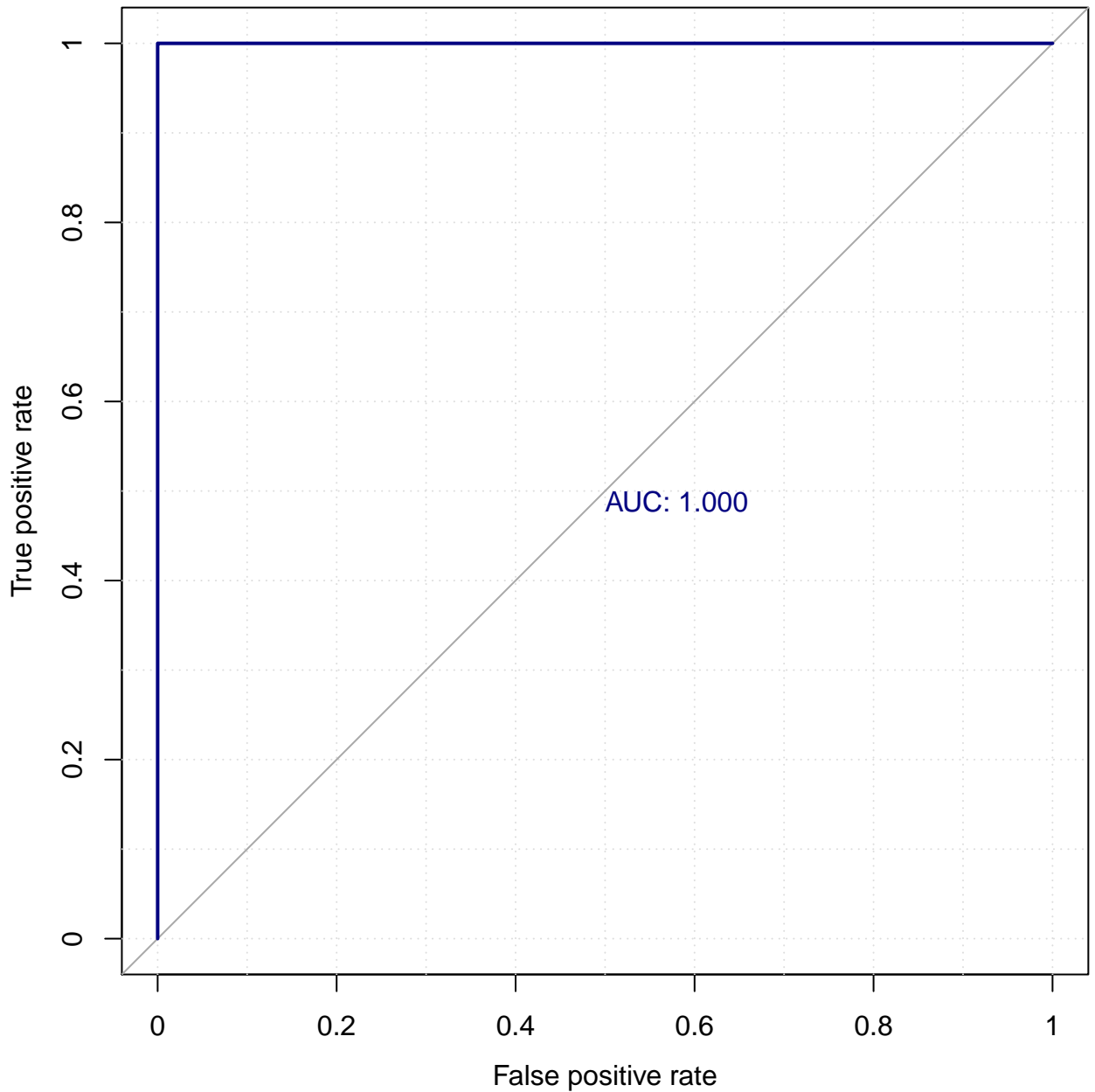

Supplement: Supplementary file 1 [file ijms-27-05895-s001.zip › result/4.MetDiffAnalysis/B_14d.vs.C_14d/ROC_all/Com_13_neg_ROC.pdf]

B\_14d.vs.C\_14d

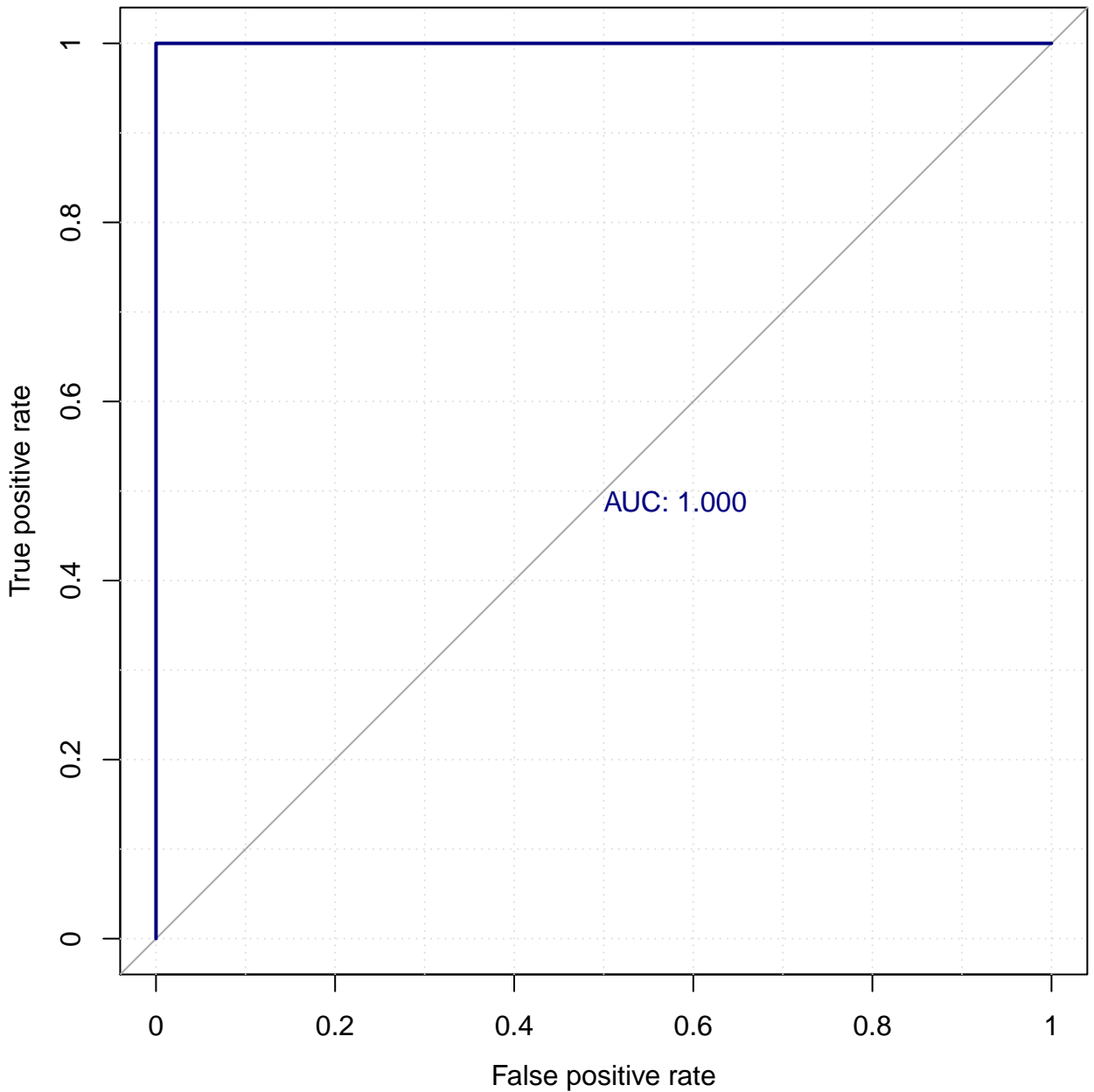

Supplement: Supplementary file 1 [file ijms-27-05895-s001.zip › result/4.MetDiffAnalysis/B_14d.vs.C_14d/ROC_all/Com_1470_pos_ROC.pdf]

B\_14d.vs.C\_14d

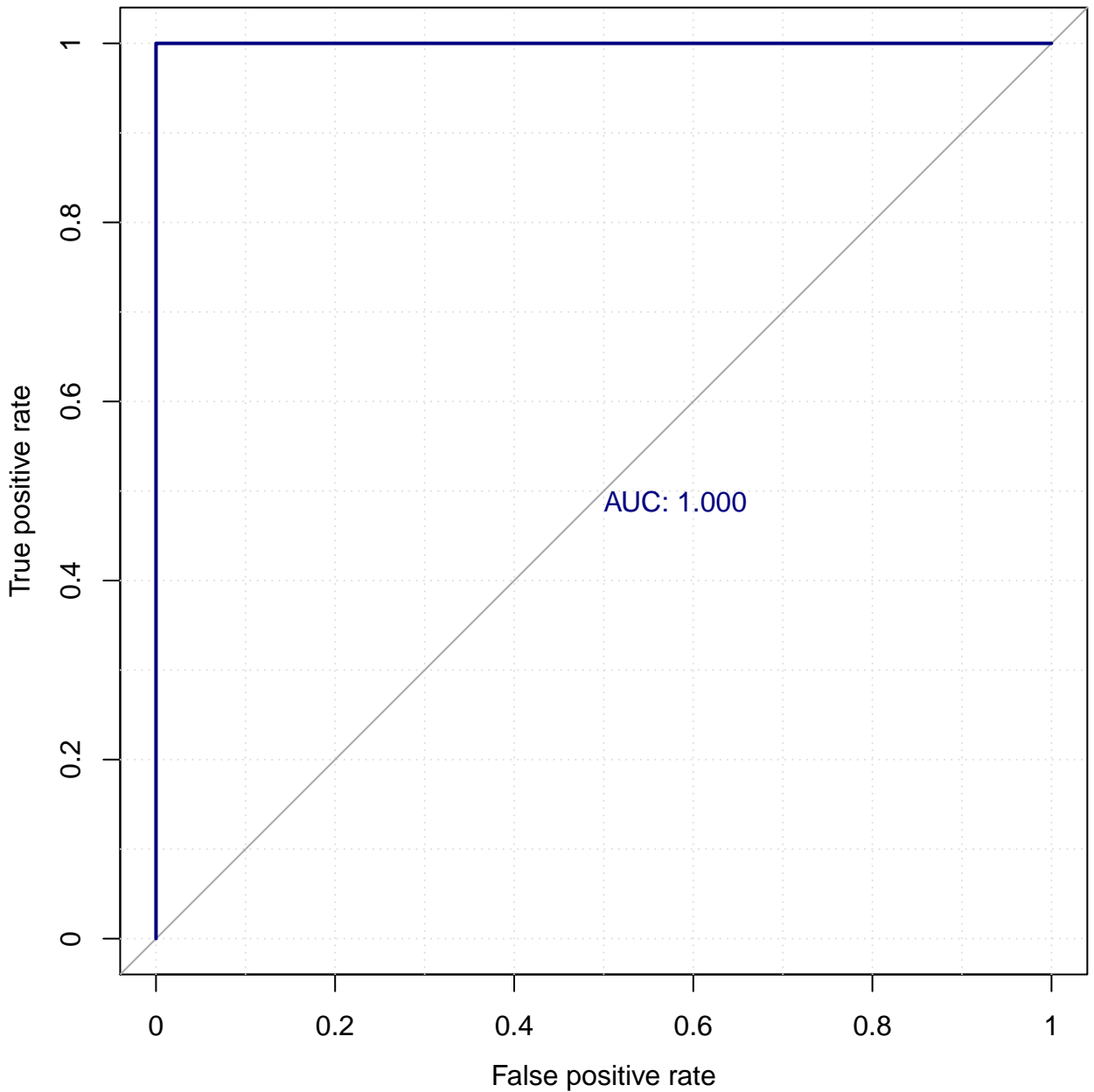

Supplement: Supplementary file 1 [file ijms-27-05895-s001.zip › result/4.MetDiffAnalysis/B_14d.vs.C_14d/ROC_all/Com_1559_pos_ROC.pdf]

B\_14d.vs.C\_14d

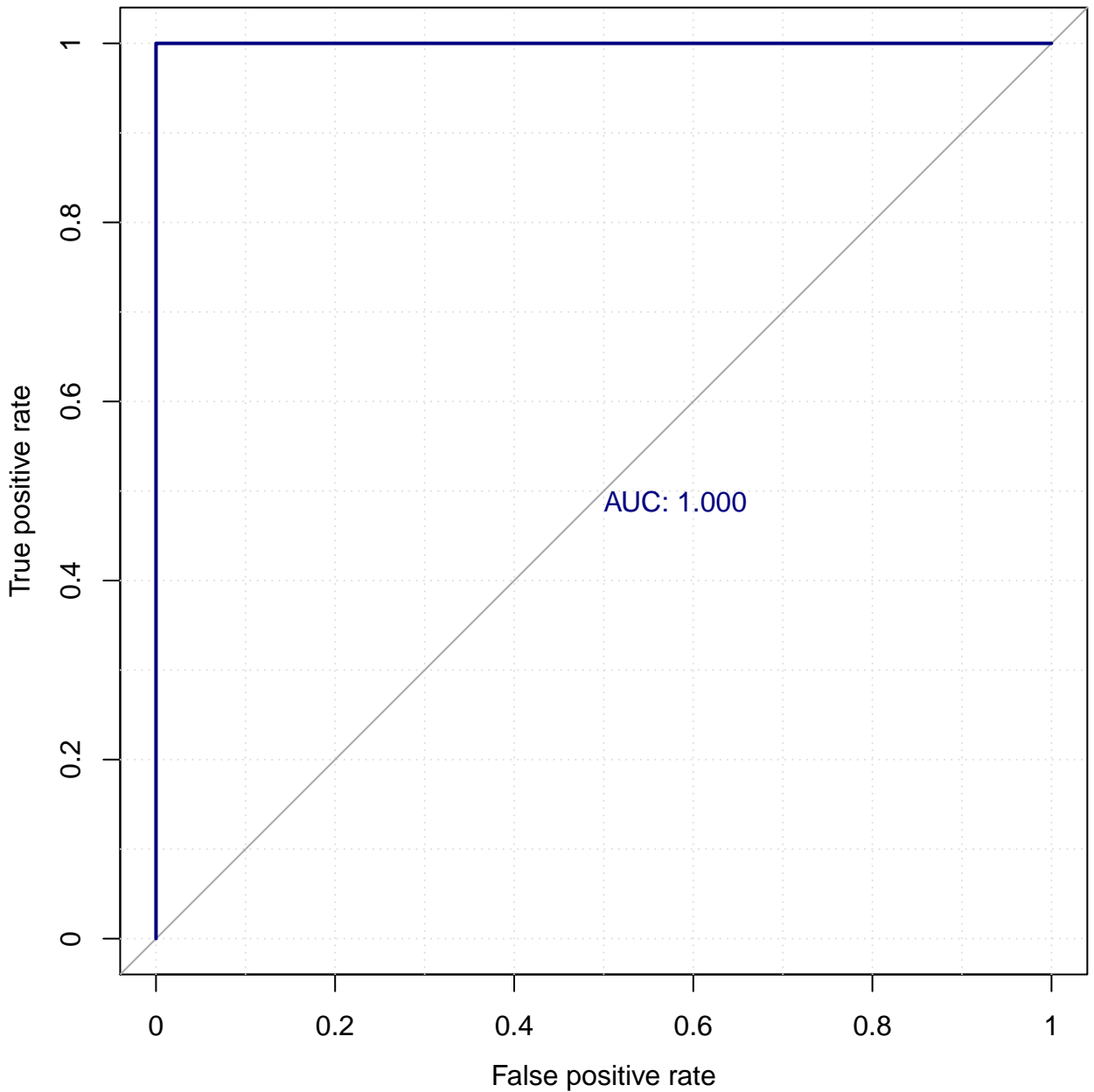

Supplement: Supplementary file 1 [file ijms-27-05895-s001.zip › result/4.MetDiffAnalysis/B_14d.vs.C_14d/ROC_all/Com_1587_neg_ROC.pdf]

B\_14d.vs.C\_14d

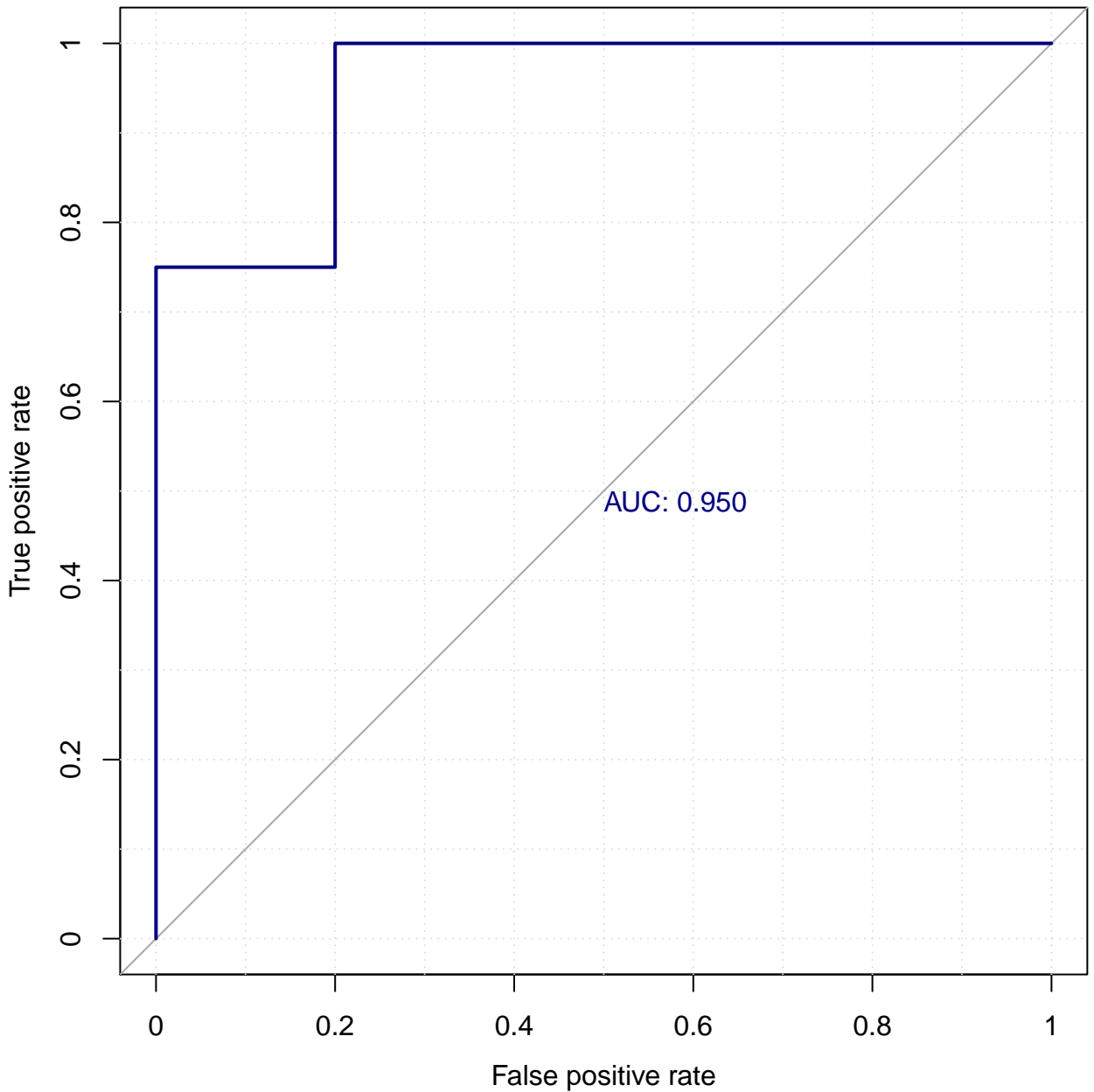

Supplement: Supplementary file 1 [file ijms-27-05895-s001.zip › result/4.MetDiffAnalysis/B_14d.vs.C_14d/ROC_all/Com_1630_neg_ROC.pdf]

B\_14d.vs.C\_14d

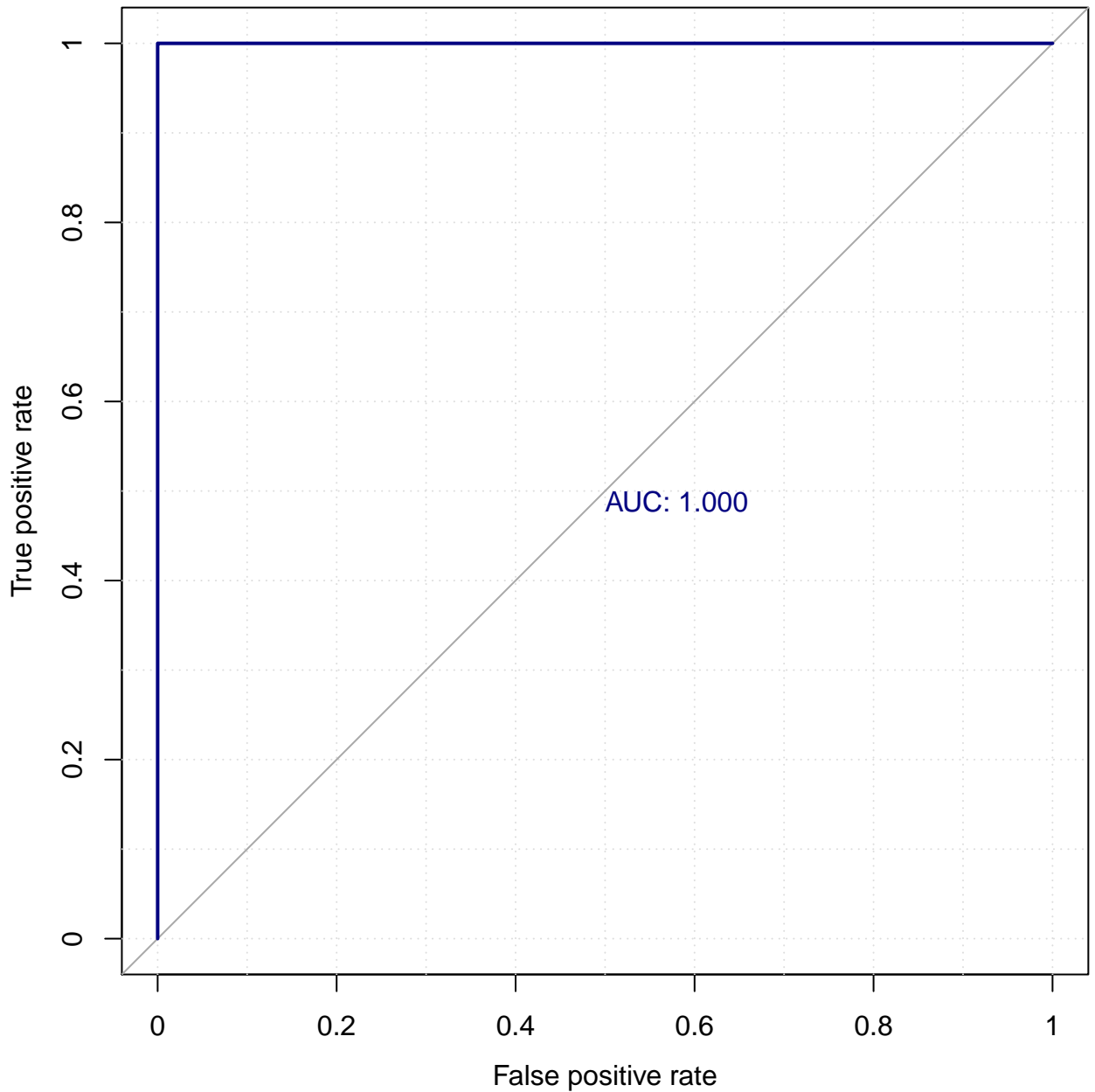

Supplement: Supplementary file 1 [file ijms-27-05895-s001.zip › result/4.MetDiffAnalysis/B_14d.vs.C_14d/ROC_all/Com_1643_pos_ROC.pdf]

B\_14d.vs.C\_14d

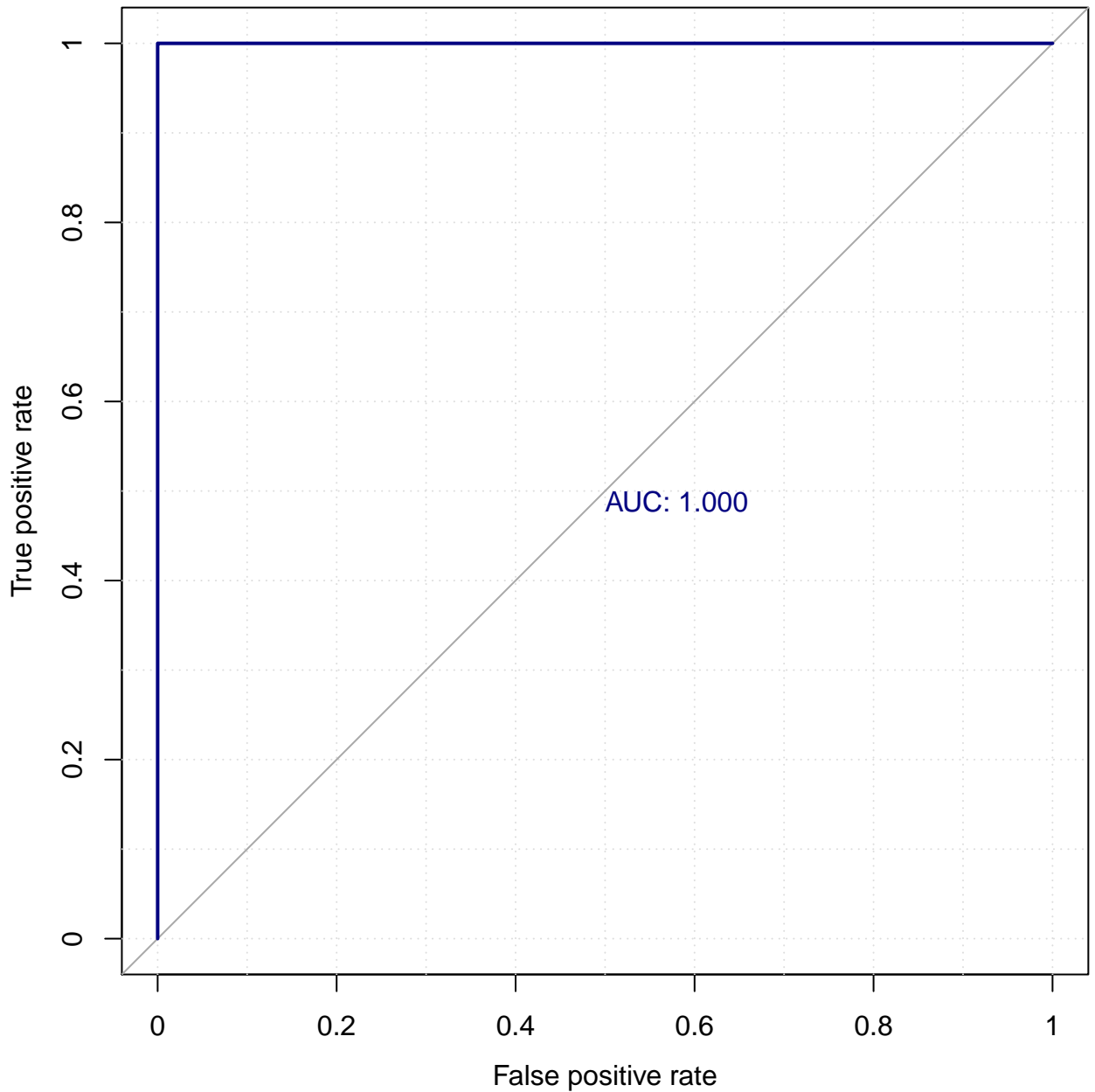

Supplement: Supplementary file 1 [file ijms-27-05895-s001.zip › result/4.MetDiffAnalysis/B_14d.vs.C_14d/ROC_all/Com_1679_neg_ROC.pdf]

B\_14d.vs.C\_14d

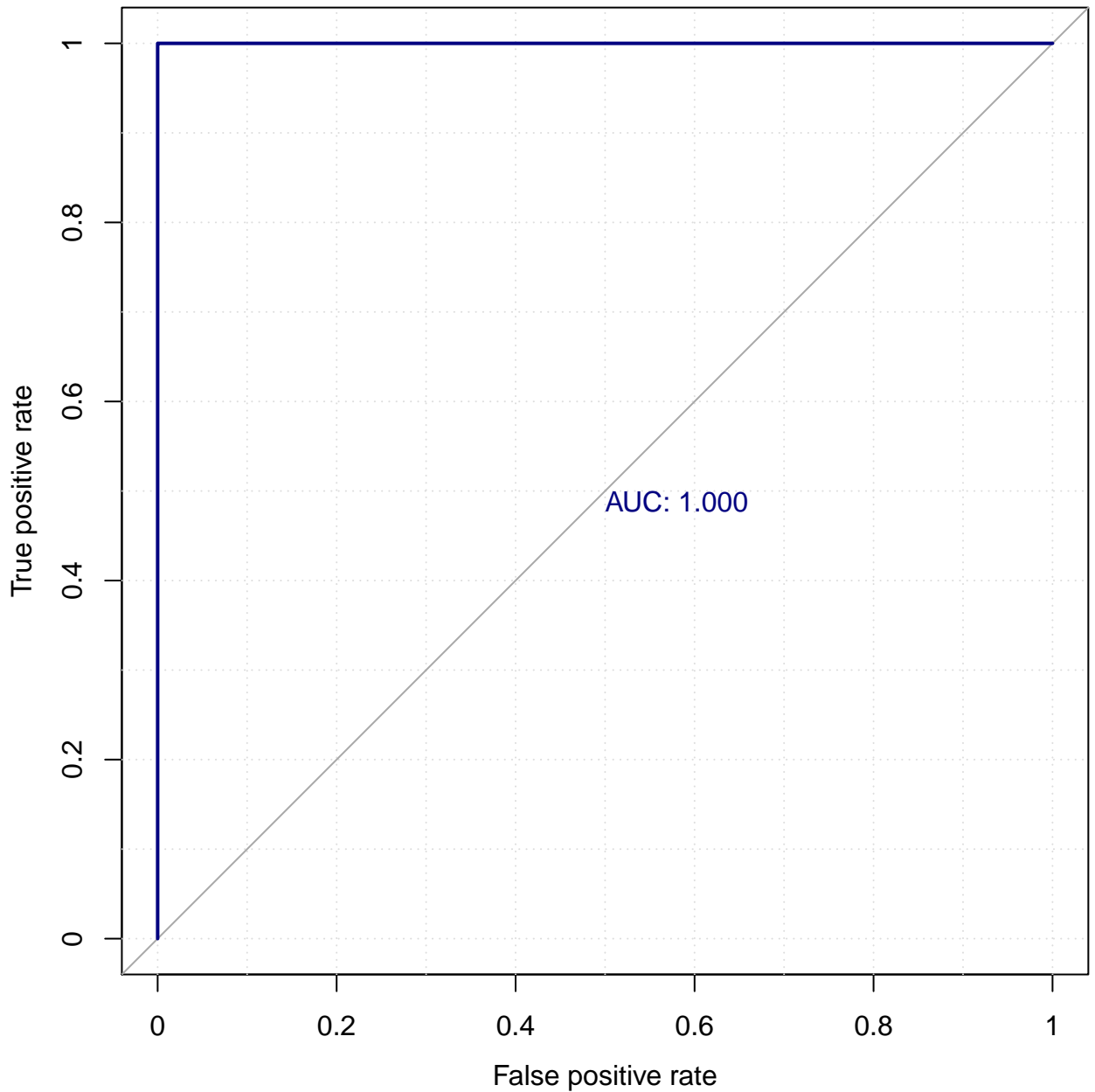

Supplement: Supplementary file 1 [file ijms-27-05895-s001.zip › result/4.MetDiffAnalysis/B_14d.vs.C_14d/ROC_all/Com_1695_neg_ROC.pdf]

B\_14d.vs.C\_14d

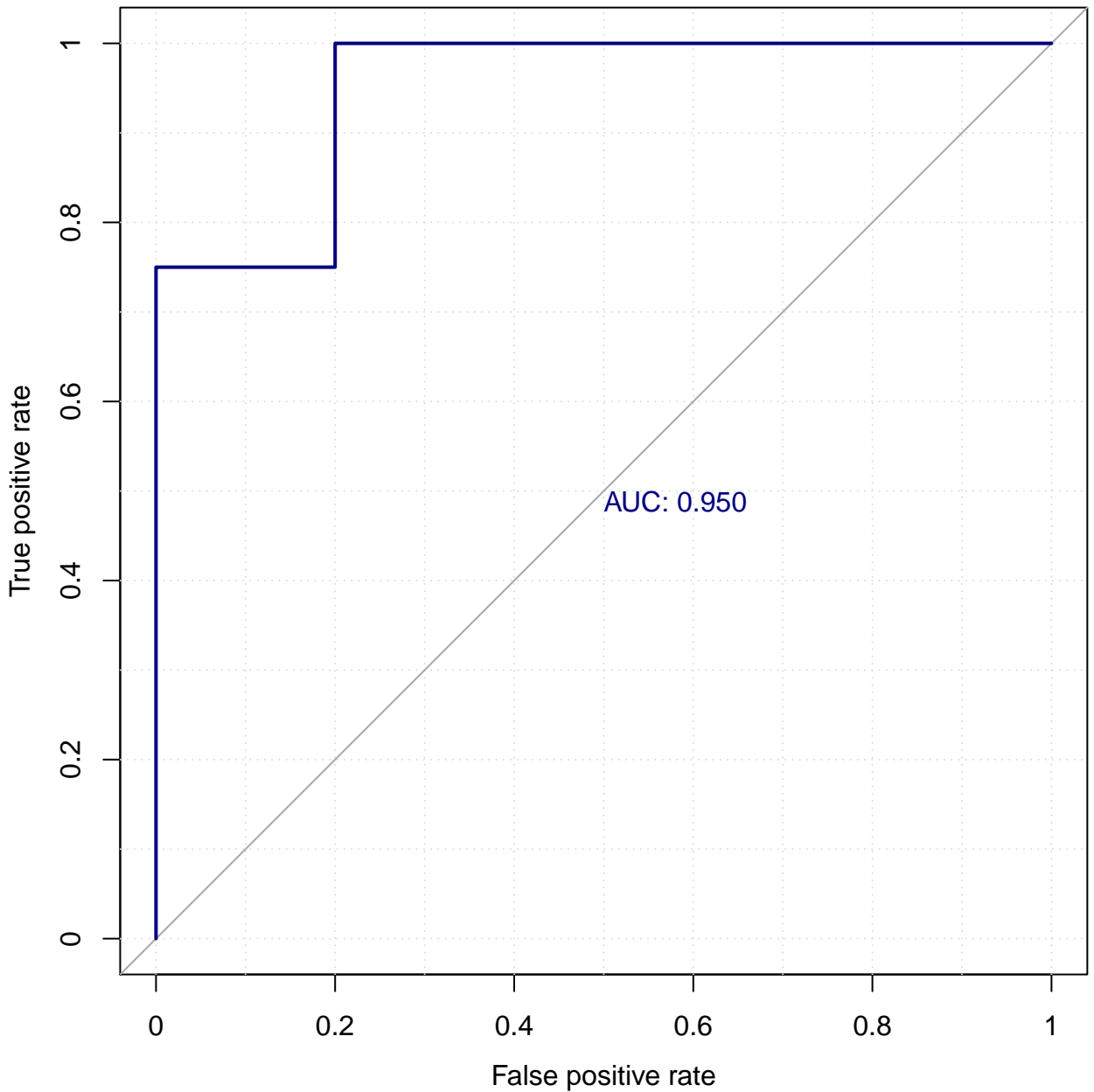

Supplement: Supplementary file 1 [file ijms-27-05895-s001.zip › result/4.MetDiffAnalysis/B_14d.vs.C_14d/ROC_all/Com_1718_neg_ROC.pdf]

B\_14d.vs.C\_14d

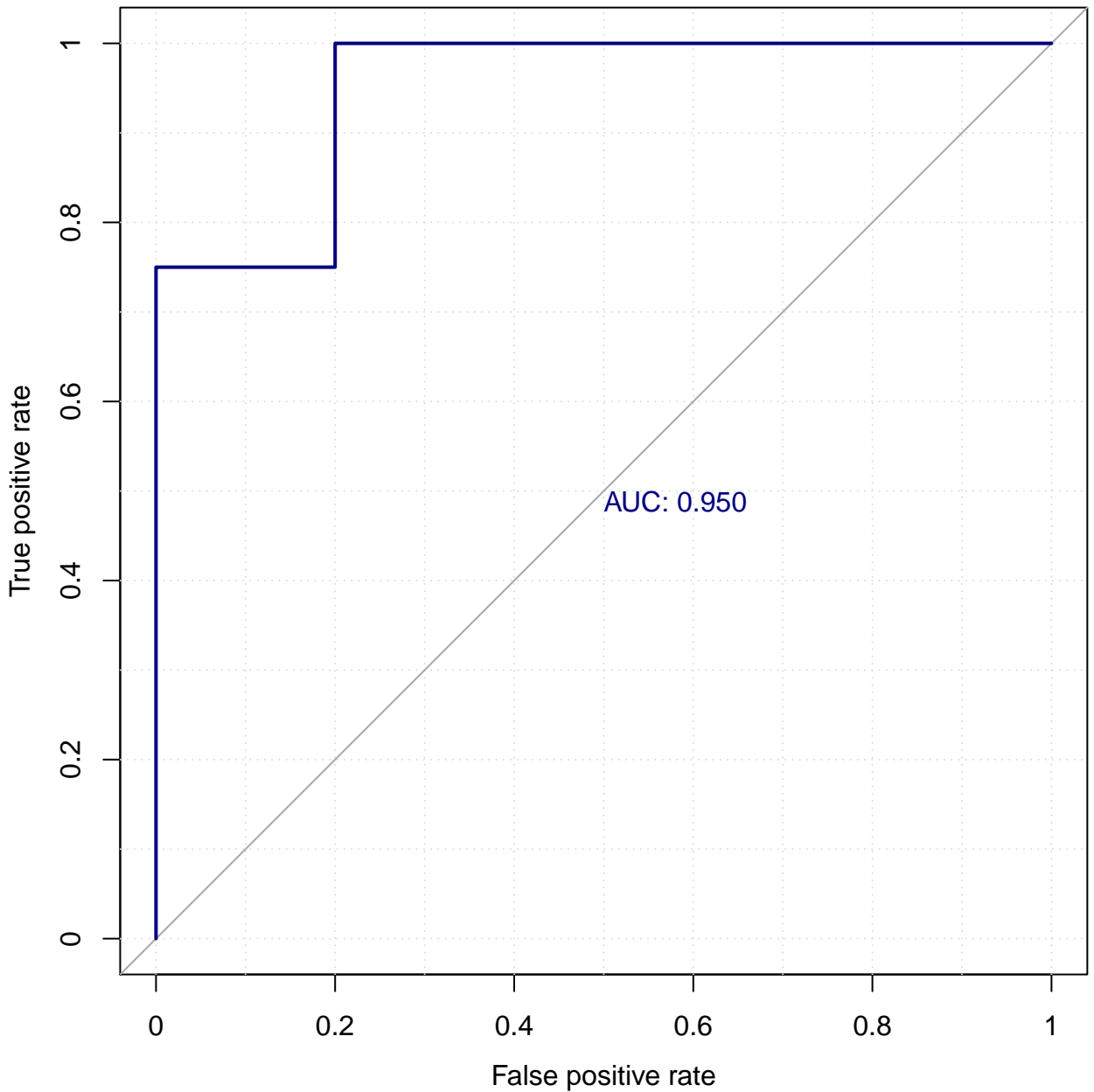

Supplement: Supplementary file 1 [file ijms-27-05895-s001.zip › result/4.MetDiffAnalysis/B_14d.vs.C_14d/ROC_all/Com_1731_pos_ROC.pdf]

B\_14d.vs.C\_14d

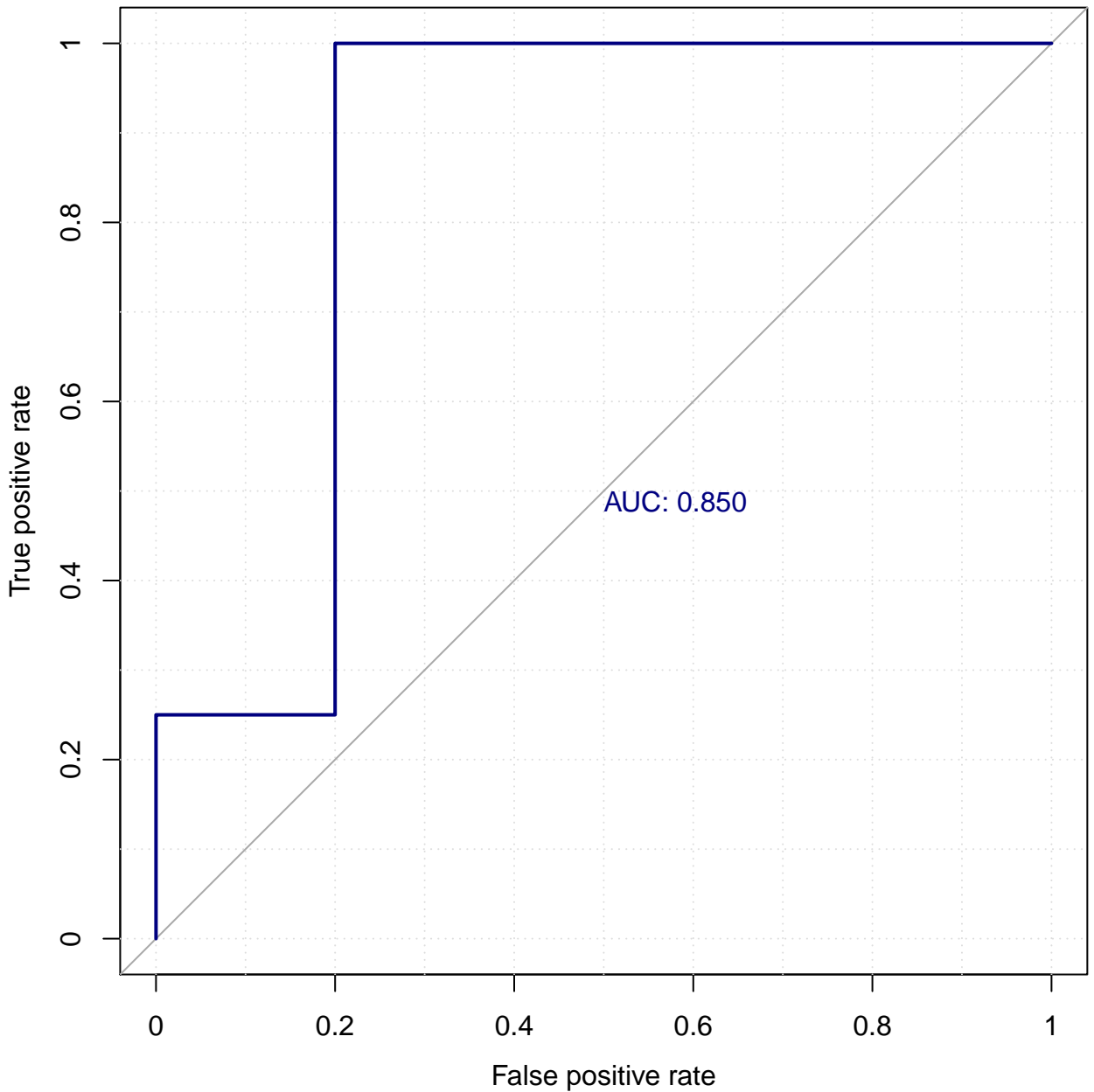

Supplement: Supplementary file 1 [file ijms-27-05895-s001.zip › result/4.MetDiffAnalysis/B_14d.vs.C_14d/ROC_all/Com_186_neg_ROC.pdf]

B\_14d.vs.C\_14d

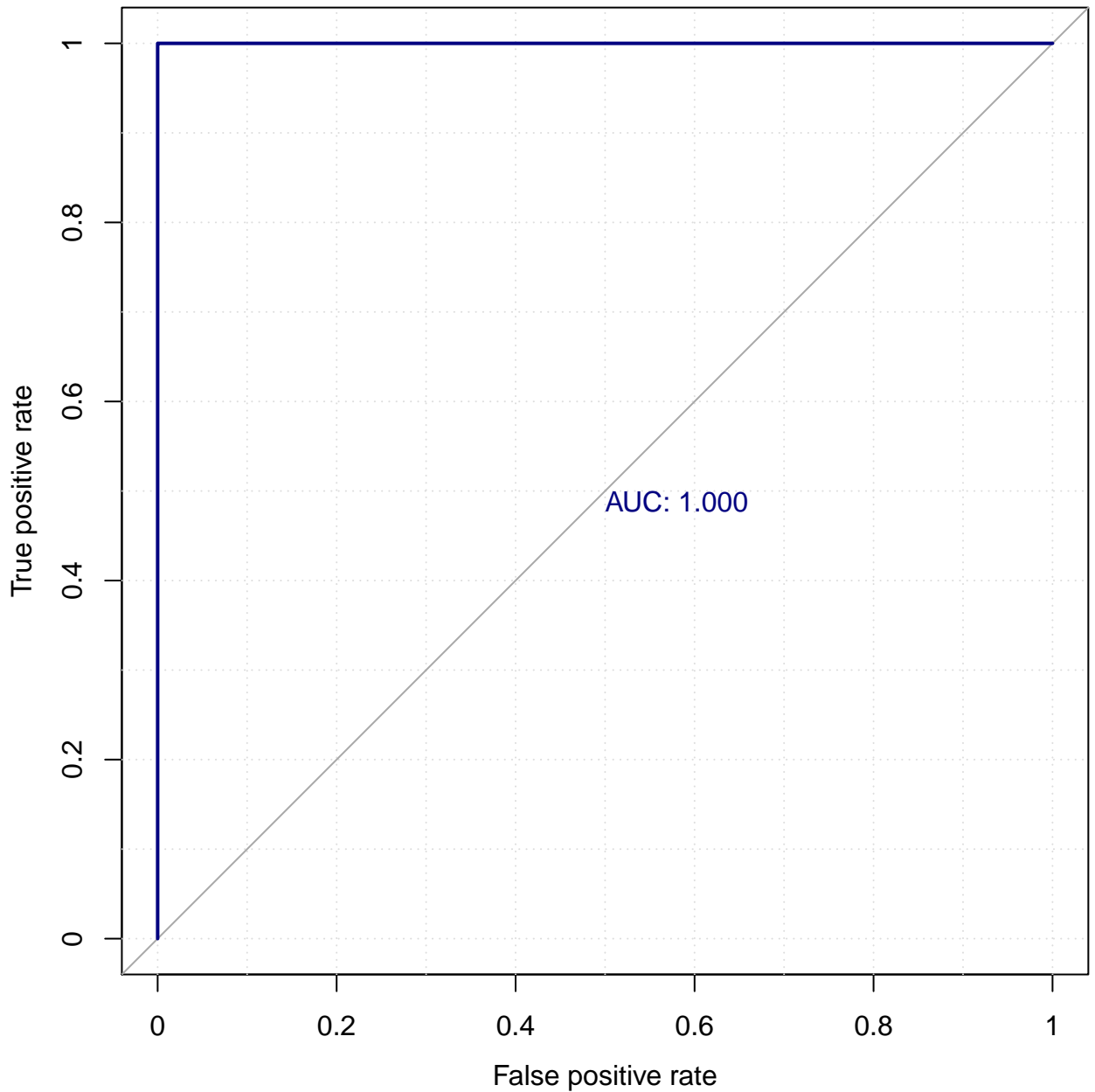

Supplement: Supplementary file 1 [file ijms-27-05895-s001.zip › result/4.MetDiffAnalysis/B_14d.vs.C_14d/ROC_all/Com_1944_pos_ROC.pdf]

B\_14d.vs.C\_14d

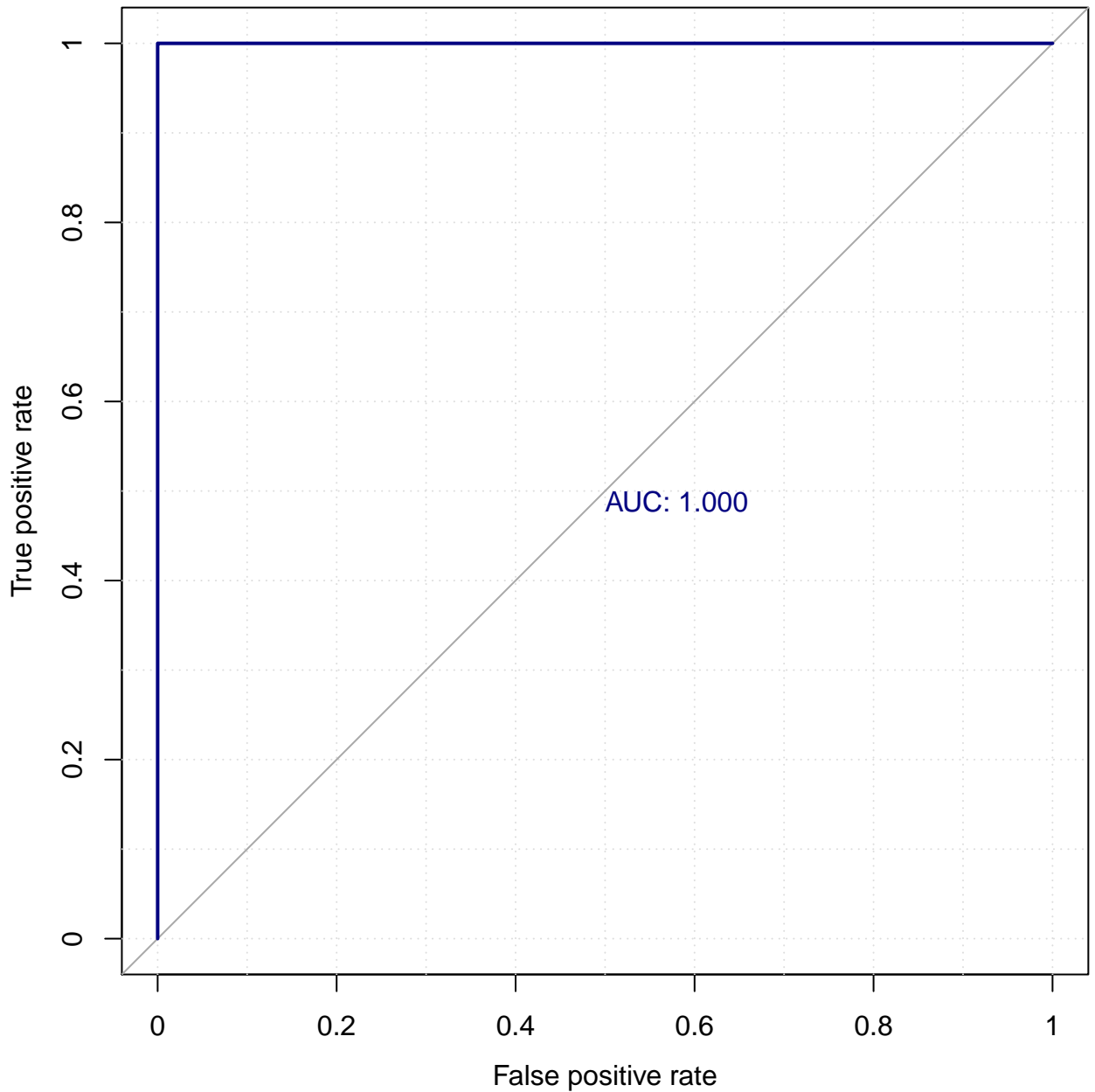

Supplement: Supplementary file 1 [file ijms-27-05895-s001.zip › result/4.MetDiffAnalysis/B_14d.vs.C_14d/ROC_all/Com_206_pos_ROC.pdf]

B\_14d.vs.C\_14d

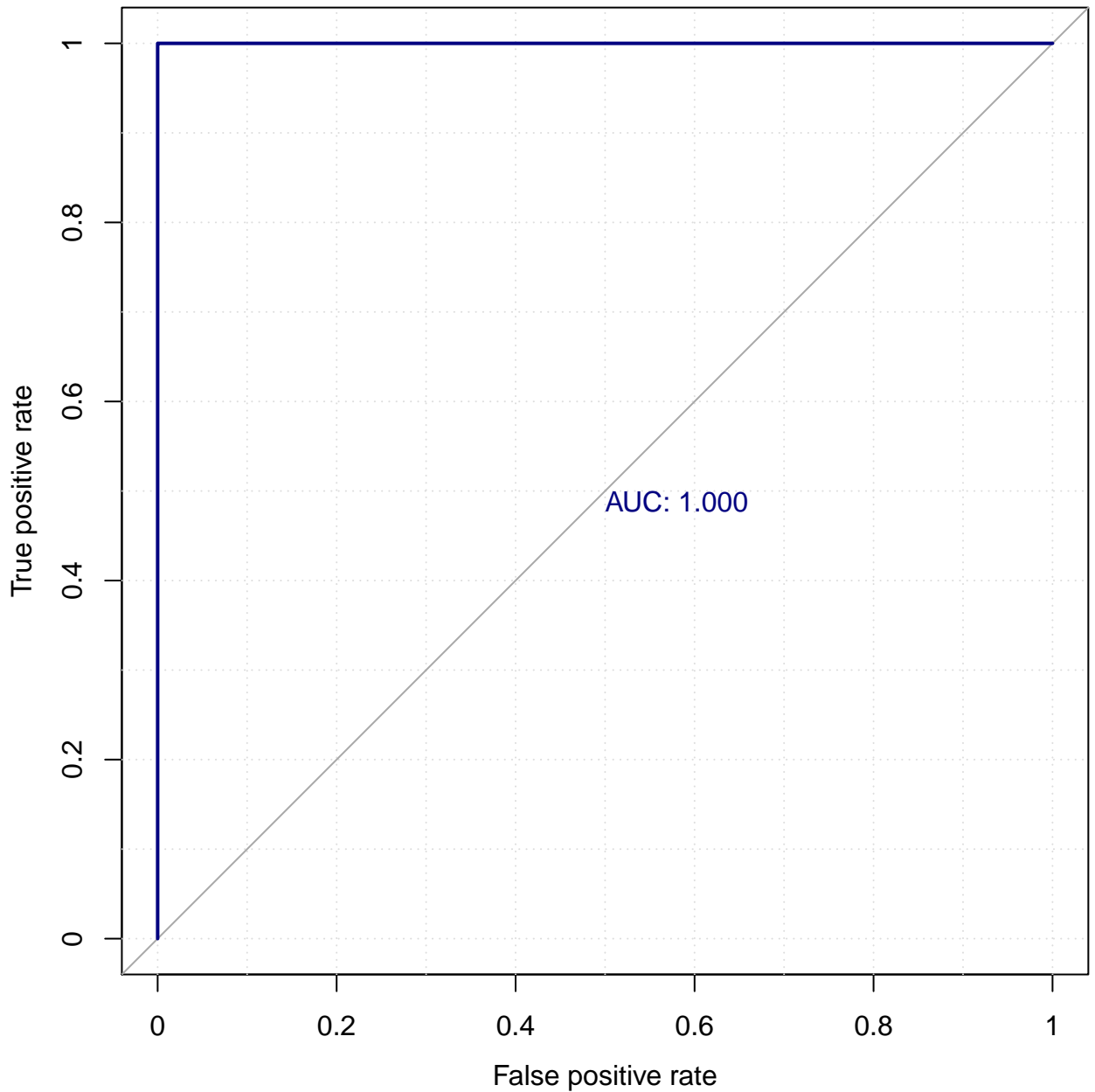

Supplement: Supplementary file 1 [file ijms-27-05895-s001.zip › result/4.MetDiffAnalysis/B_14d.vs.C_14d/ROC_all/Com_2331_pos_ROC.pdf]

B\_14d.vs.C\_14d

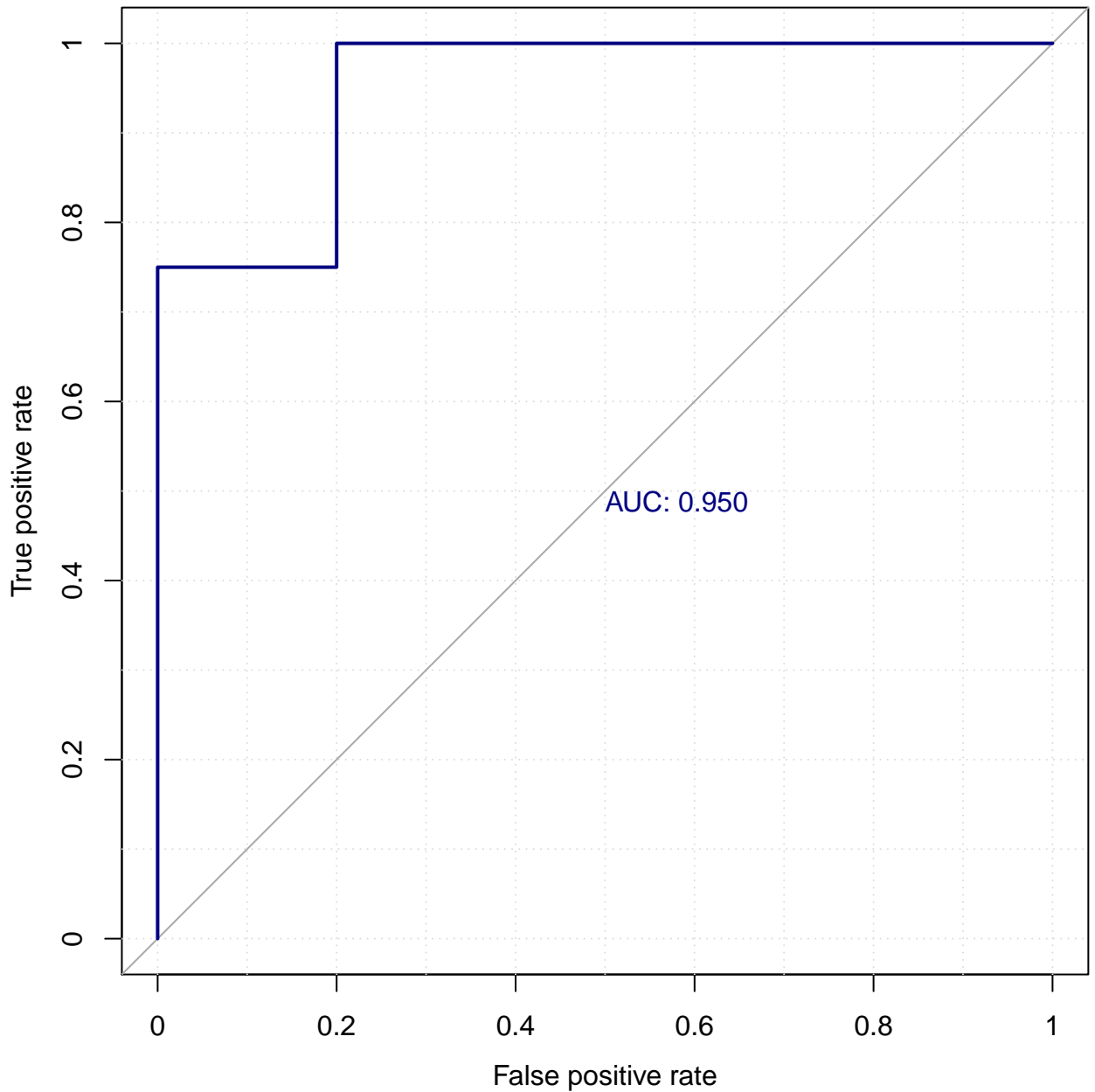

Supplement: Supplementary file 1 [file ijms-27-05895-s001.zip › result/4.MetDiffAnalysis/B_14d.vs.C_14d/ROC_all/Com_2347_pos_ROC.pdf]

B\_14d.vs.C\_14d

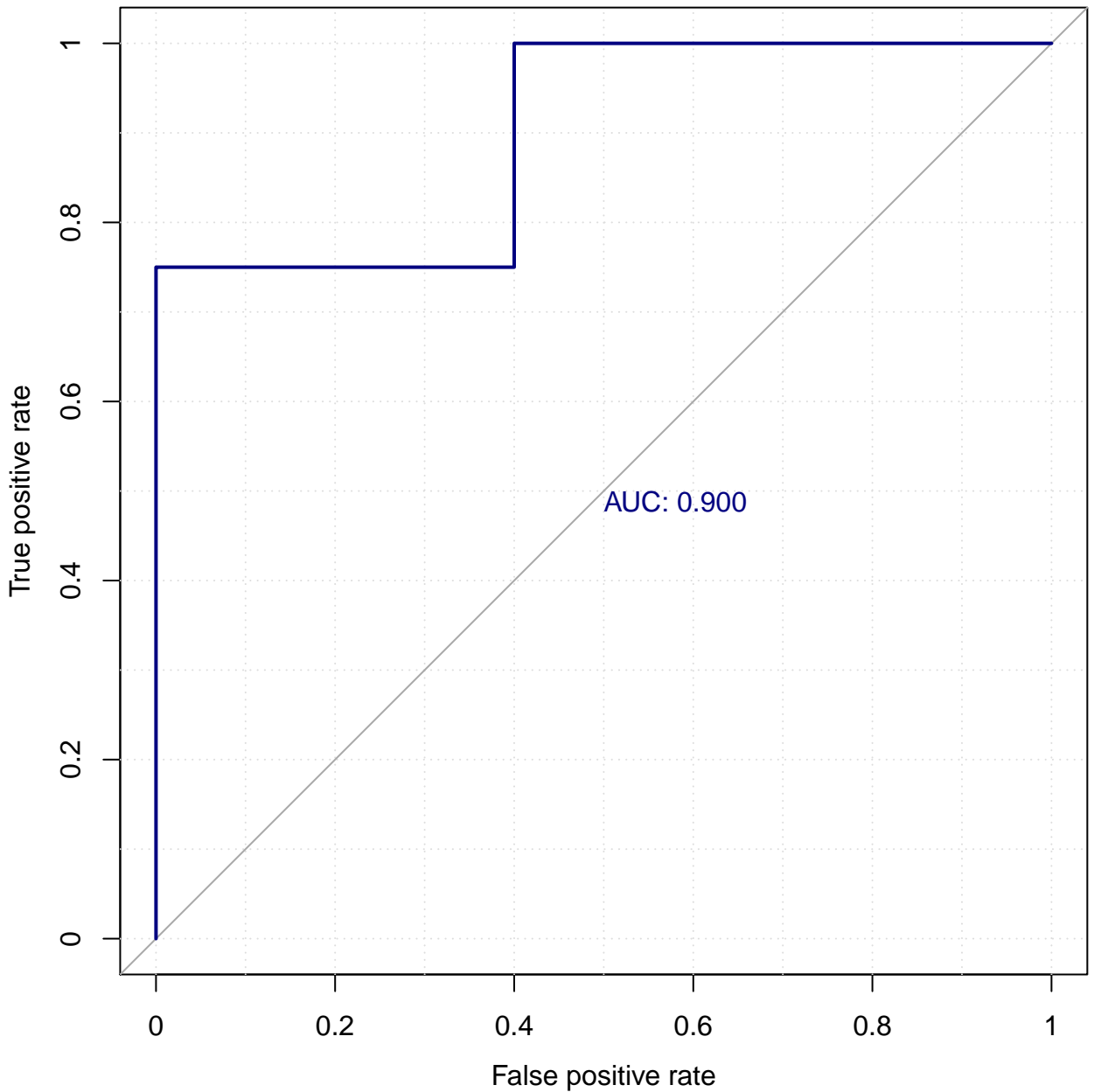

Supplement: Supplementary file 1 [file ijms-27-05895-s001.zip › result/4.MetDiffAnalysis/B_14d.vs.C_14d/ROC_all/Com_2449_pos_ROC.pdf]

B\_14d.vs.C\_14d

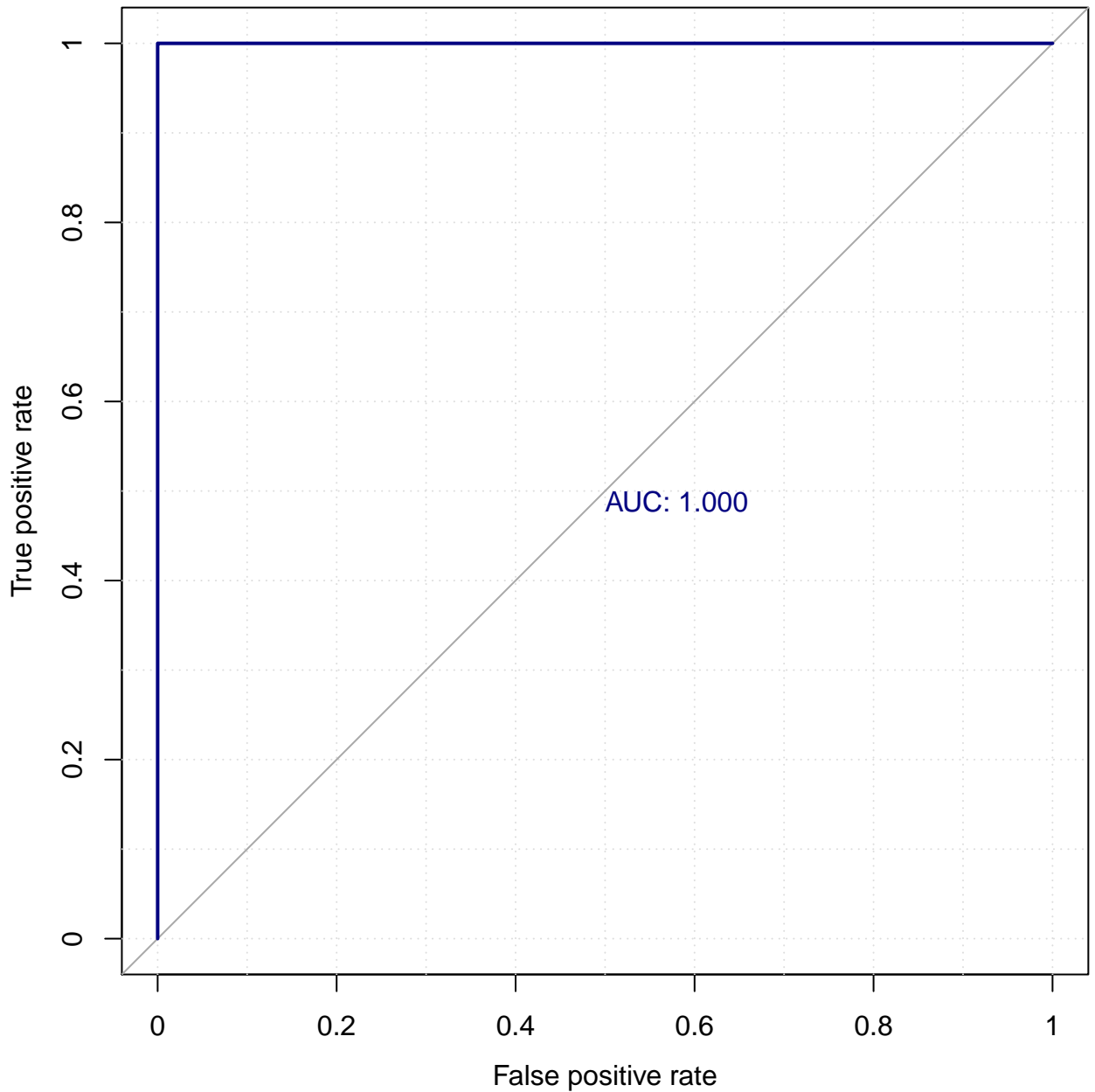

Supplement: Supplementary file 1 [file ijms-27-05895-s001.zip › result/4.MetDiffAnalysis/B_14d.vs.C_14d/ROC_all/Com_2469_pos_ROC.pdf]

B\_14d.vs.C\_14d

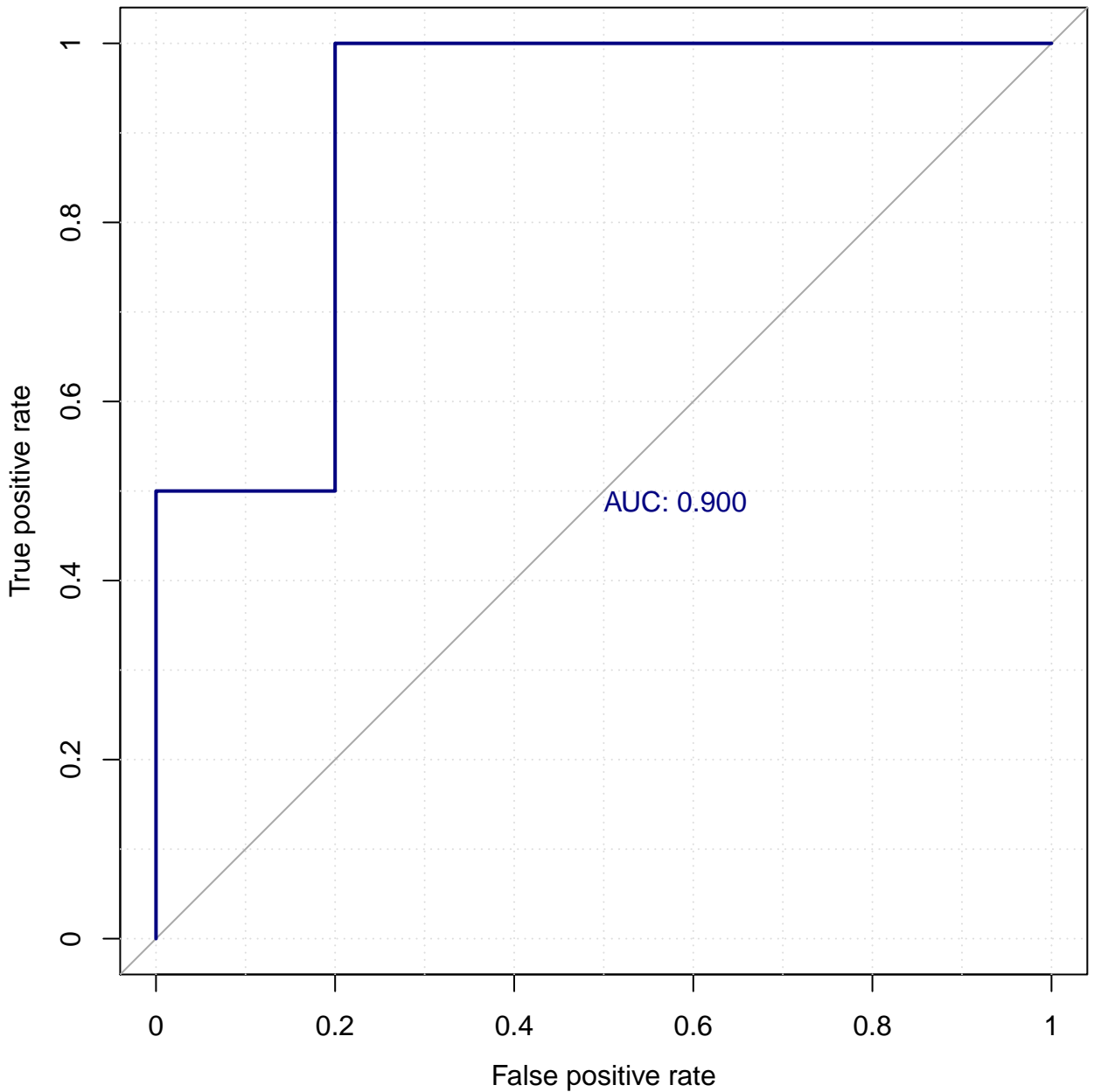

Supplement: Supplementary file 1 [file ijms-27-05895-s001.zip › result/4.MetDiffAnalysis/B_14d.vs.C_14d/ROC_all/Com_2595_pos_ROC.pdf]

B\_14d.vs.C\_14d

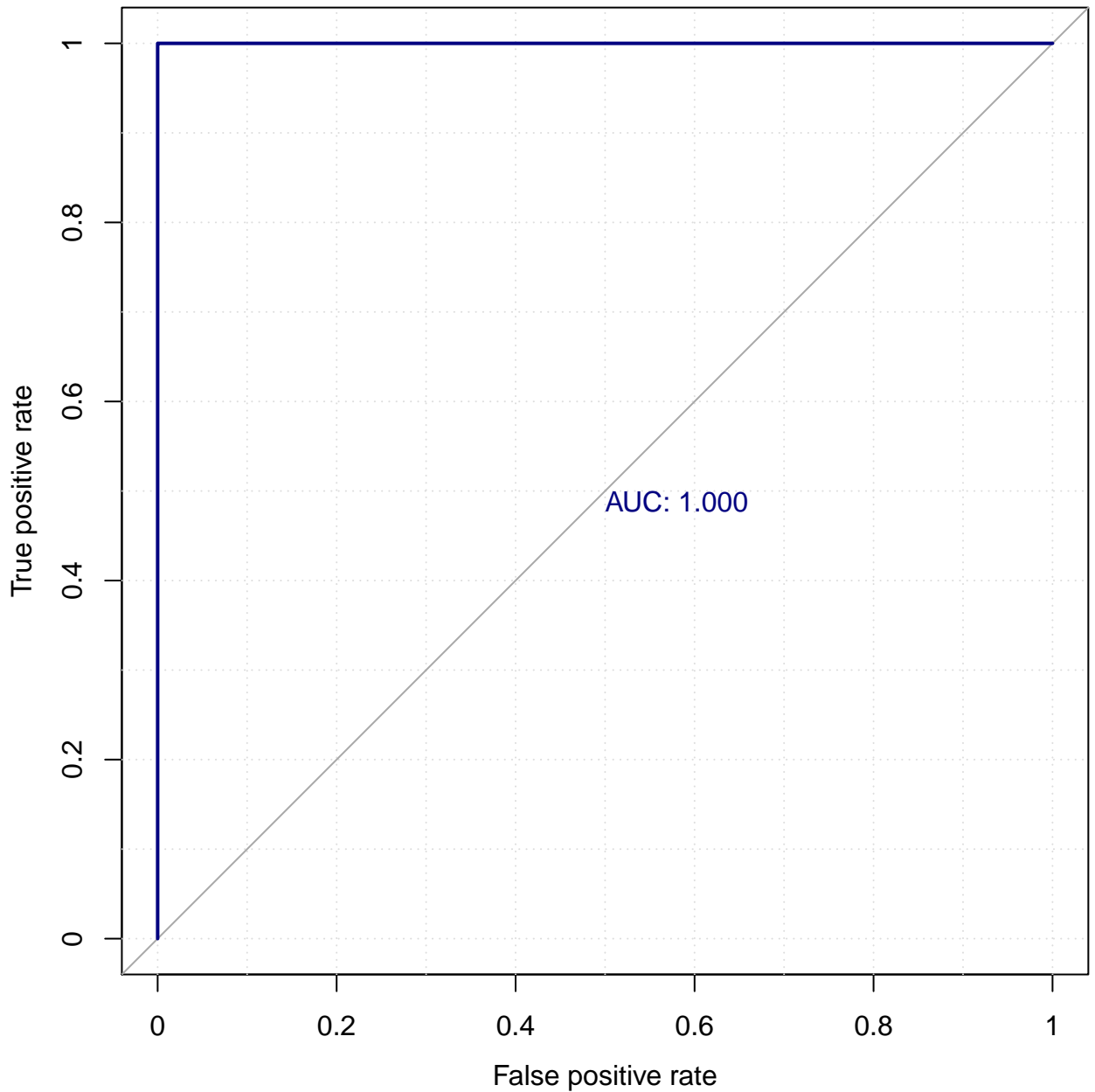

Supplement: Supplementary file 1 [file ijms-27-05895-s001.zip › result/4.MetDiffAnalysis/B_14d.vs.C_14d/ROC_all/Com_2695_pos_ROC.pdf]

B\_14d.vs.C\_14d

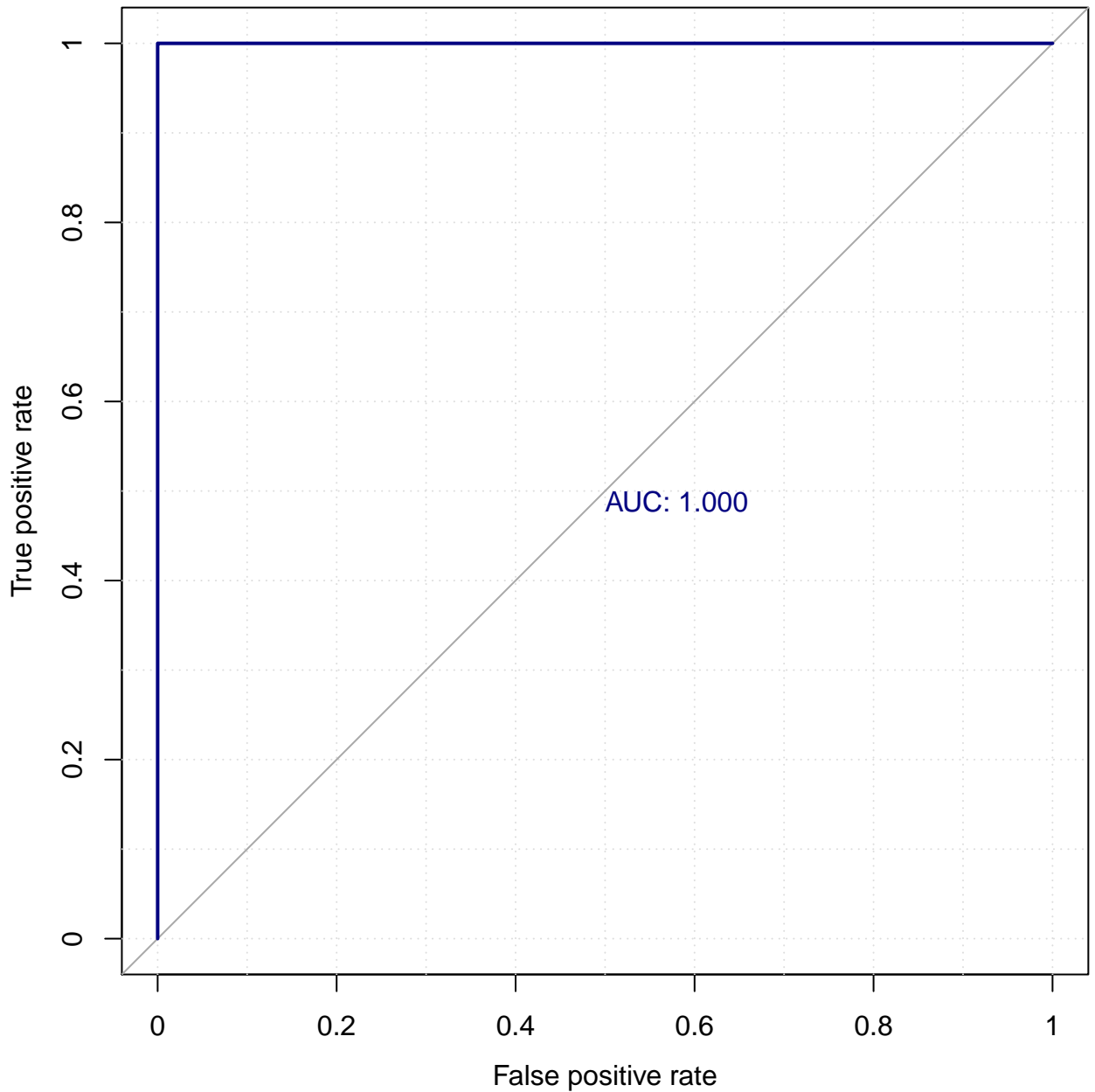

Supplement: Supplementary file 1 [file ijms-27-05895-s001.zip › result/4.MetDiffAnalysis/B_14d.vs.C_14d/ROC_all/Com_2741_pos_ROC.pdf]

B\_14d.vs.C\_14d

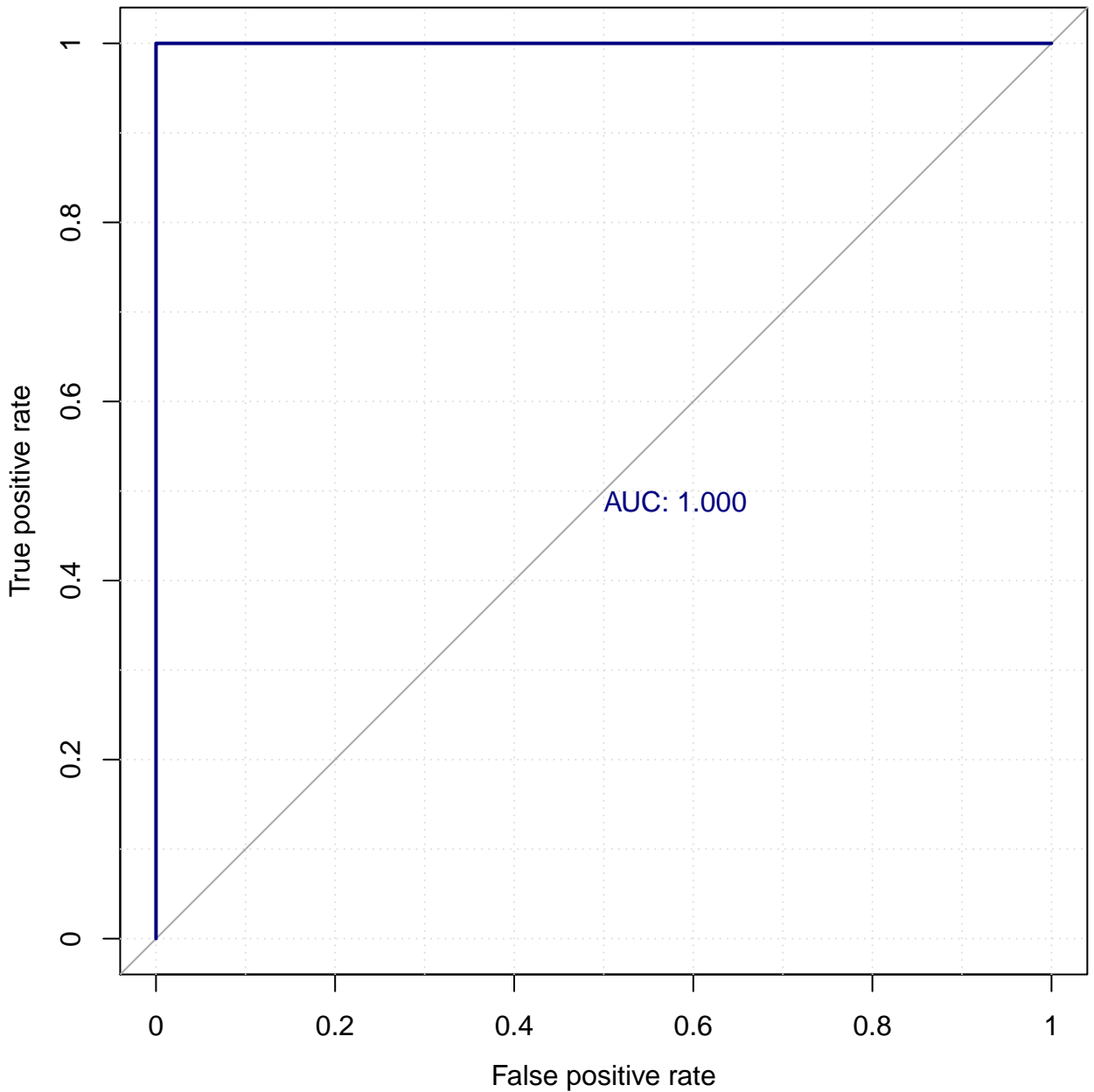

Supplement: Supplementary file 1 [file ijms-27-05895-s001.zip › result/4.MetDiffAnalysis/B_14d.vs.C_14d/ROC_all/Com_2963_pos_ROC.pdf]

B\_14d.vs.C\_14d

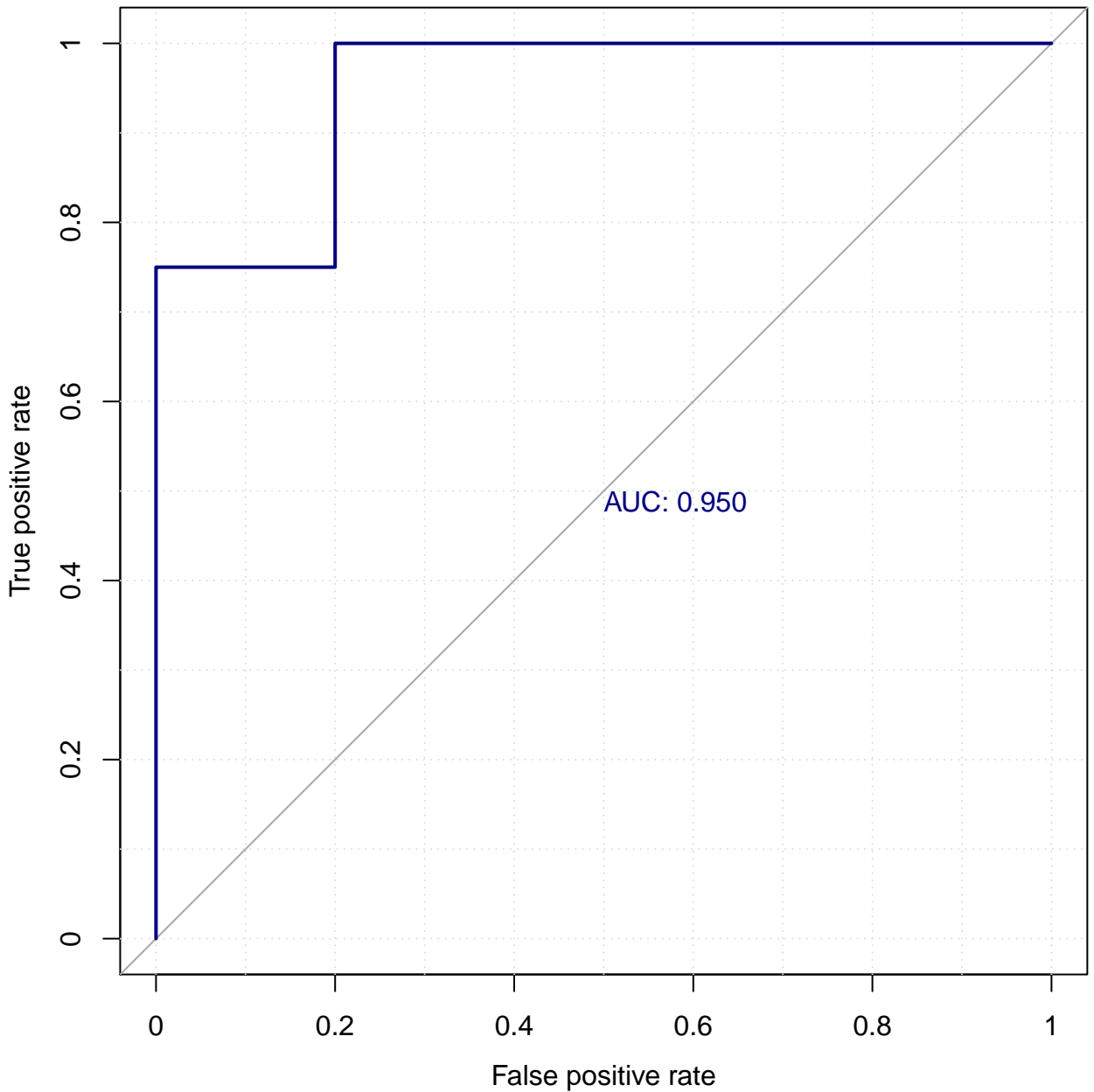

Supplement: Supplementary file 1 [file ijms-27-05895-s001.zip › result/4.MetDiffAnalysis/B_14d.vs.C_14d/ROC_all/Com_3522_pos_ROC.pdf]

B\_14d.vs.C\_14d

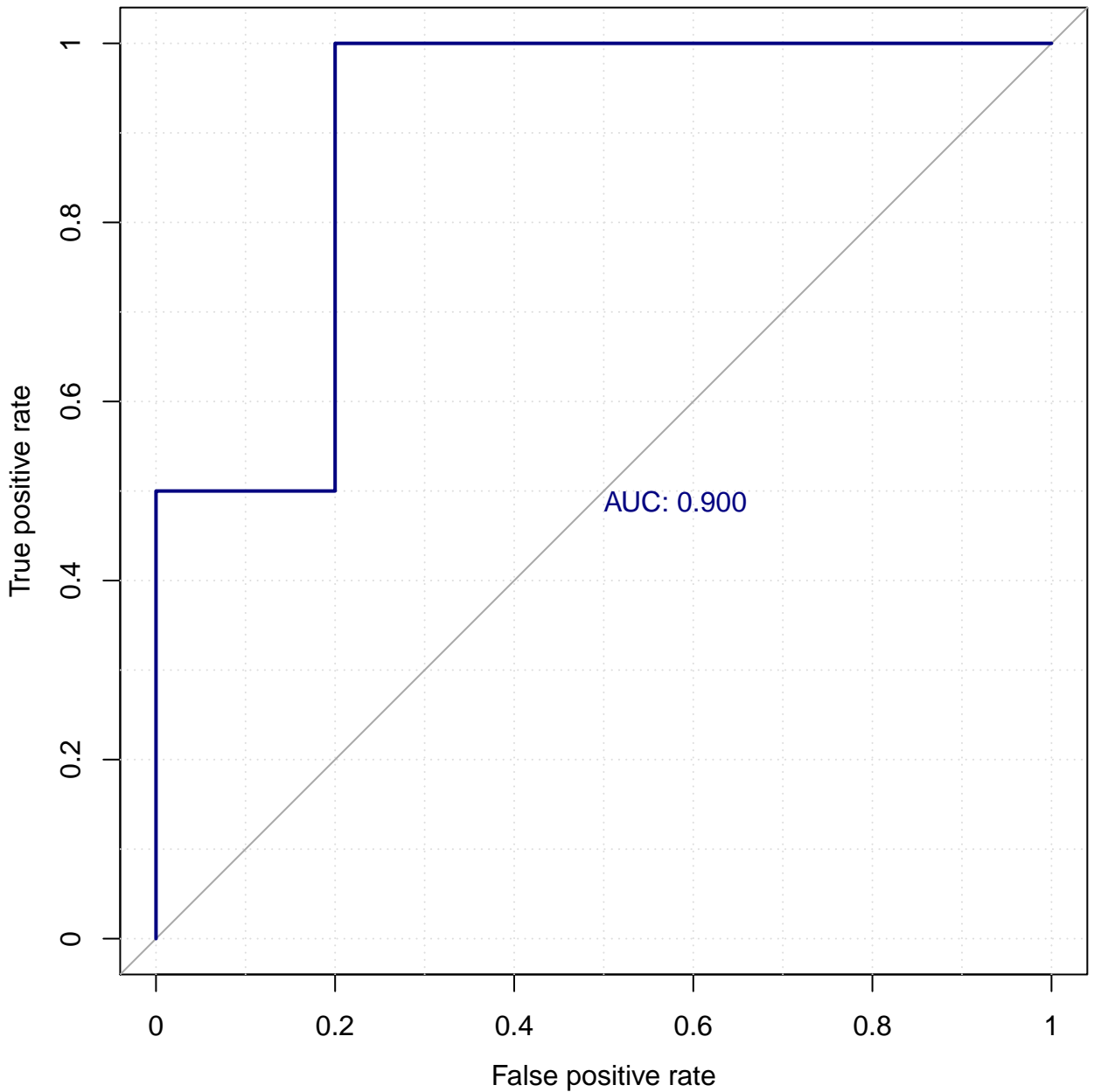

Supplement: Supplementary file 1 [file ijms-27-05895-s001.zip › result/4.MetDiffAnalysis/B_14d.vs.C_14d/ROC_all/Com_361_pos_ROC.pdf]
